# An examination of multiple classes of rare variants in extended families with bipolar disorder

**DOI:** 10.1038/s41398-018-0113-y

**Published:** 2018-03-13

**Authors:** Claudio Toma, Alex D. Shaw, Richard J. N. Allcock, Anna Heath, Kerrie D. Pierce, Philip B. Mitchell, Peter R. Schofield, Janice M. Fullerton

**Affiliations:** 1 Neuroscience Research Australia, Sydney, Australia; 2 School of Medical Sciences, University of New South Wales, Sydney, Australia; 3 School of Pathology and Laboratory Medicine, University of Western Australia, Perth, Australia; 4 School of Psychiatry, University of New South Wales, Sydney, Australia; 5 Black Dog Institute, Prince of Wales Hospital, Sydney, Australia

## Abstract

Bipolar disorder (BD) is a complex psychiatric condition with high heritability, the genetic architecture of which likely comprises both common variants of small effect and rare variants of higher penetrance, the latter of which are largely unknown. Extended families with high density of illness provide an opportunity to map novel risk genes or consolidate evidence for existing candidates, by identifying genes carrying pathogenic rare variants. We performed whole-exome sequencing (WES) in 15 BD families (117 subjects, of whom 72 were affected), augmented with copy number variant (CNV) microarray data, to examine contributions of multiple classes of rare genetic variants within a familial context. Linkage analysis and haplotype reconstruction using WES-derived genotypes enabled exclusion of false-positive single-nucleotide variants (SNVs), CNV inheritance estimation, de novo variant identification and candidate gene prioritization. We found that rare predicted pathogenic variants shared among ≥3 affected relatives were overrepresented in postsynaptic density (PSD) genes (*P* = 0.002), with no enrichment in unaffected relatives. Genome-wide burden of likely gene-disruptive variants was no different in affected vs. unaffected relatives (*P* = 0.24), but correlated significantly with age of onset (*P* = 0.017), suggesting that a high disruptive variant burden may expedite symptom onset. The number of de novo variants was no different in affected vs. unaffected offspring (*P* = 0.89). We observed heterogeneity within and between families, with the most likely genetic model involving alleles of modest effect and reduced penetrance: a possible exception being a truncating X-linked mutation in *IRS4* within a family-specific linkage peak. Genetic approaches combining WES, CNV and linkage analyses in extended families are promising strategies for gene discovery.

## Introduction

Bipolar disorder (BD) is a common, complex psychiatric condition characterized by recurrent fluctuations of mood, often accompanied by psychotic features^1^. Age of onset is typically in the teenage years or early 20s, although episodes may also first appear in mid-life^2^. Despite twin and family studies providing evidence for high heritability (*H*^2^∼80%)^3^, the specific genetic factors that increase risk remain largely elusive. Historically, linkage studies of multiplex families failed to identify replicable linkage signals across families, indicating genetic heterogeneity and a complex underlying genetic architecture. More recently, large-scale genome-wide association studies (GWAS) have implicated common variants in *CACNA1C*, *ANK3* and *ODZ4* as being significantly associated with BD^4–6^. However, the effect sizes of these common disease-associated variants are small, and when the cumulative effect of thousands of common variants are considered under an additive model they explain only one-fourth of the variance in liability^7^, suggesting that heritable factors not captured by GWAS may contribute to disease etiology. BD genetic architecture is thus likely to comprise a combination of common and rare variants, across multiple classes of variation, including single-nucleotide variants (SNVs), copy number variants (CNVs) and de novo variants, although the relative contribution of each class of variants is unclear. The role of rare CNVs in BD risk is still controversial^8–11^, and although large rare CNVs (>100 kb) have been implicated in BD susceptibility, their effects seem marginal when compared with schizophrenia^10,12^.

The rapid development of next-generation sequencing technology has facilitated studies examining the impact of rare SNVs in multiplex or extended BD families, shedding light on pathways and genes that may be implicated in the disease^13–18^. Ament and colleagues employed a candidate gene and pathway approach, using 41 BD families from the NIMH Genomics Initiative (*n* = 200 subjects)^16^, finding rare segregating variants enriched in neuronal ion-channel genes, and discovering rare variant associations with existing candidates *(ANK3* and *CACNA1C)*. Goes and colleagues sequenced eight multiplex BD families (*n* = 36 cases), identifying enrichment of genes harboring damaging SNVs in those genes previously reported to carry de novo mutations in schizophrenia or autism^18^. Georgi and colleagues sequenced 18 parent–child trios from a large single Old Order Amish pedigree of 388 relatives, finding no convergence of risk loci on particular pathways^14^. In a study of 79 trios by Kataoka and colleagues, de novo variants were associated with earlier age of onset, and were enriched in previously associated psychiatric risk genes^19^, consistent with earlier reports of de novo variation playing a role in sporadic cases of autism and schizophrenia^20,21^. No study has thus far simultaneously examined all classes of DNA variation in a familial context.

We therefore performed a comprehensive analysis of multiple classes of rare DNA variants in 15 Australian multiplex and extended families with a high density of illness (≥4 cases per family) using whole-exome sequencing (WES), augmented by CNV and linkage analysis in order to identify genes and pathways that may contribute to the pathophysiology of BD. We considered: (i) predicted pathogenic SNVs and likely gene-disruptive variants shared in ≥3 affected relatives, as compared with those shared in unaffected relatives; (ii) the impact of genome-wide burden of likely gene-disruptive variants on BD; (iii) de novo variants in familial BD; (iv) the role of rare CNVs; and (v) rare variants with potential higher penetrance under top linkage peaks for individual families.

## Materials and methods

### Bipolar family and subject selection

**Fig. 1:**
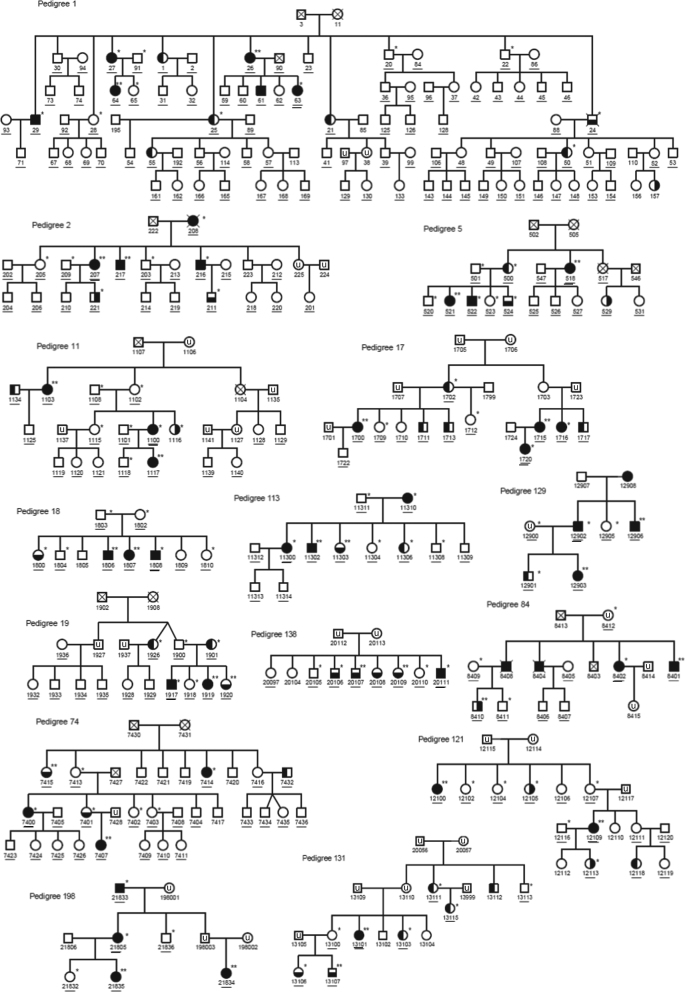
Structure of the 15 pedigrees examined in our study. Males are indicated with squares, females with circles, and psychiatric diagnoses are shown by dark shading (full shading = bipolar disorder type I (BD-I), right shading = bipolar disorder type II (BD-II, left shading = recurrent unipolar depression (RUD), bottom shading = schizoaffective disorder-manic type (SZMA), unshaded = unaffected, u = unknown). Individuals included in the exome study are indicated by single asterisks, and subjects with both WES and CytoScanHD array data are indicated by double asterisks. All subjects with DNA available are underlined, and subjects with chip-based genotype data from which multidimensional scaling analysis and polygenic risk scores were generated are indicated with a second underline

From our collection of 65 multiplex BD families^22^, we selected 15 extended families of European descent with a high density of illness, as defined by ≥4 first- or second-degree relatives with BD-I, BD-II or schizoaffective disorder-manic type (SZMA) (Fig. 1). Individuals were selected for WES based on diagnostic phenotype, availability and quality of DNA, and pedigree structure using ExomePicks (http://genome.sph.umich.edu/wiki/ExomePicks). The sequenced sample included 117 subjects: 72 affected relatives, 28 unaffected relatives and 17 unaffected parents. Details on clinical assessments are provided in Supplementary Information (Section 1). Multidimension scaling (MDS) analysis to derive ethnicity from genotype data, examination of genetic relatedness (identity-by-descent (IBD)) analysis to confirm inheritance models and common polygenic risk estimation (PRS) are provided in Supplementary Information (Section 2).

### Exome capture, sequencing and alignment

Exome enrichment, template sequencing and initial variant calling were performed at the Lotterywest State Biomedical Facility (Perth, Australia). AmpliSeq exome enrichment and Ion Proton sequencing were performed as previously described^23^. Alignment to human reference genome (hg19:GRCh37.p5) and initial variant calling was performed separately for each individual using Torrent Suite V4.2 (ThermoFisher, Waltham, MA, USA), followed by a backfilling procedure within Torrent Variant Caller v4.2.3, with ‘hotspot’ parameter set to force genotype calling at all variant sites across all subjects. The mean read depth was very high at 112×, and on average, 92% of bases captured were covered by >10 reads.

### Rare variant selection and prediction of pathogenicity

Rare variants were defined as having a European minor allele frequency (MAF) <1% in Exome Variant Server (EVS, http://evs.gs.washington.edu/EVS/), dbSNP 138 and 1000 genomes Phase I integrated call set. Intersection with Refseq gene models was assessed using Plink-Seq (http://atgu.mgh.harvard.edu/plinkseq/download.shtml), which indicates the ‘worst’ (most damaging) class of functional change where more than one gene model overlaps the variant of interest. Missense variants were annotated using Polyphen 2 HVAR and SIFT from dbNSFP v2.8^24,25^. Polyphen 2 and SIFT scores were combined into a CAROL score, in order to improve effect prediction for nonsynonymous coding variants^26^, and variants with CAROL ≥0.99 were selected as potentially pathogenic. Likely gene-disruptive variants were defined as nonsense, indels leading to frameshift, canonical splice variants and start-lost.

### WES-based linkage analysis, haplotype reconstruction and validation

Genotypes were called from aligned reads using samtools pileup and filtered to include haplotype-informative markers (HapMap CEU population) using LINKDATAGEN^27^. WES-derived genotypes were used to confirm familial relationships by pair-wise IBD using PLINK^28^, and also to perform haplotype phasing and linkage analysis in each family under both parametric and non-parametric models using Merlin^29^. Parameters for linkage analysis, as well as linkage results and SNV colocalization analyses are provided in Supplementary Material (Section 3).

Reconstructed haplotypes were visualized using HaploPainter^30^, and used to compare against inheritance of all rare potentially pathogenic variants in each family: those inconsistent with underlying haplotype (or where haplotype was uninformative) were validated by PCR and Sanger sequencing.

### CNV analysis

Genome-wide CNV analysis was performed via CytoScan® HD Array (Affymetrix, Santa Clara, CA, USA) in two affected relatives per family (*n* = 30 individuals). All rare CNVs (MAF < 0.05) spanning at least 25 kb (minimum of 25 probes) were considered. We also considered WES-derived genotypes for consecutive Mendelian inconsistencies to identify likely CNV deletions. Additional support of CNVs and pedigree segregation was provided via three complementary methods: (i) analysis of SNP array data (Illumina 660 quad, one patient per family) with PennCNV^31^; (ii) CNVs derived from WES read depth using EXCAVATOR^32^; and (iii) using haplotypes to infer CNV segregation among relatives. All CNVs were validated through quantitative PCR (qPCR). Full details of CNV study methods are provided in Supplementary Information in Section 4.

### De novo variant analysis

We selected all nuclear families from within the 15 extended families for whom we had WES for both parents and their offspring, regardless of diagnosis. This yielded 32 offspring individuals from 9 of the 15 families for de novo assessment, 22 of which were affected offspring and 10 unaffected offspring (represented in Supplemental Figure S7). All variants with potential Mendelian error from WES genotypes were screened for adequate read depth (AD > 20), and manual checking of reads in both parents and offspring, plus any additional relatives, using IGV v2.3.34 to confirm each putative de novo variant. All putative de novo variants then underwent validation by Sanger sequencing. Full details of de novo study methods are provided in Supplementary Information in Section 6.

### Statistical and bioinformatic analyses

Enrichment of genes carrying potentially pathogenic shared rare variants were tested against all gene ontology (GO) categories, and were corrected for multiple testing. In a second analysis, we performed an enrichment analysis against gene sets potentially related to disease pathophysiology, namely: genes encoding postsynaptic density (PSD) proteins^33^, FMRP interactor targets^34^, de novo variants in autism, schizophrenia and intellectual disability^20^, *N*-methyl-d-aspartate (NMDA) receptors (NMDARs)^20^, activity-regulated cytoskeleton-associated protein (ARC) complex^20^, and nuclear-encoded mitochondrial proteins^35^. Both analyses were performed by: (i) matching genes carrying potentially etiologic variants to genes with sufficient sequence coverage (>10×) randomly drawn from the genome, after approximate matching on coding-sequence length and genic constraint missense Z-score (http://exac.broadinstitute.org)^36^; and (ii) calculating an empirical *p*-value for observed data for each functional category, using a null distribution of overlap counts from 10,000 randomly drawn gene sets. Empirical *p*-values that exceed Bonferroni correction for multiple testing for were considered significant.

Non-parametric and mixed-model tests were performed to assess differences in the number and distribution of likely gene-disruptive variants between BD patients and their unaffected relatives. Spearman’s correlation was performed to assess the relationship between likely gene-disruptive variants and age of onset (*n* = 58 subjects).

Genes expressed in the brain were determined from RNA-seq data downloaded from the developmental transcriptomics data, generated across 13 developmental stages in 8–16 brain structures (http://www.brainspan.org/rnaseq/search/index.html). Normalized expression values were examined, using reads per kilobase per million mapped reads (RPKM), and those genes with an average RKPM > 1 across all samples (either across all ages and brain tissues, or limited to only prenatal or adult expression) were considered brain expressed.

Evidence of gene-level association with BD and schizophrenia was derived from summary statistics from Psychiatric Genomics Consortium GWAS^6,37^, with VEGAS2 (https://vegas2.qimrberghofer.edu.au/). Additional methods for enrichment and linear regression analyses is provided in Supplementary Material in Section 5.

## Results

**Fig. 2:**
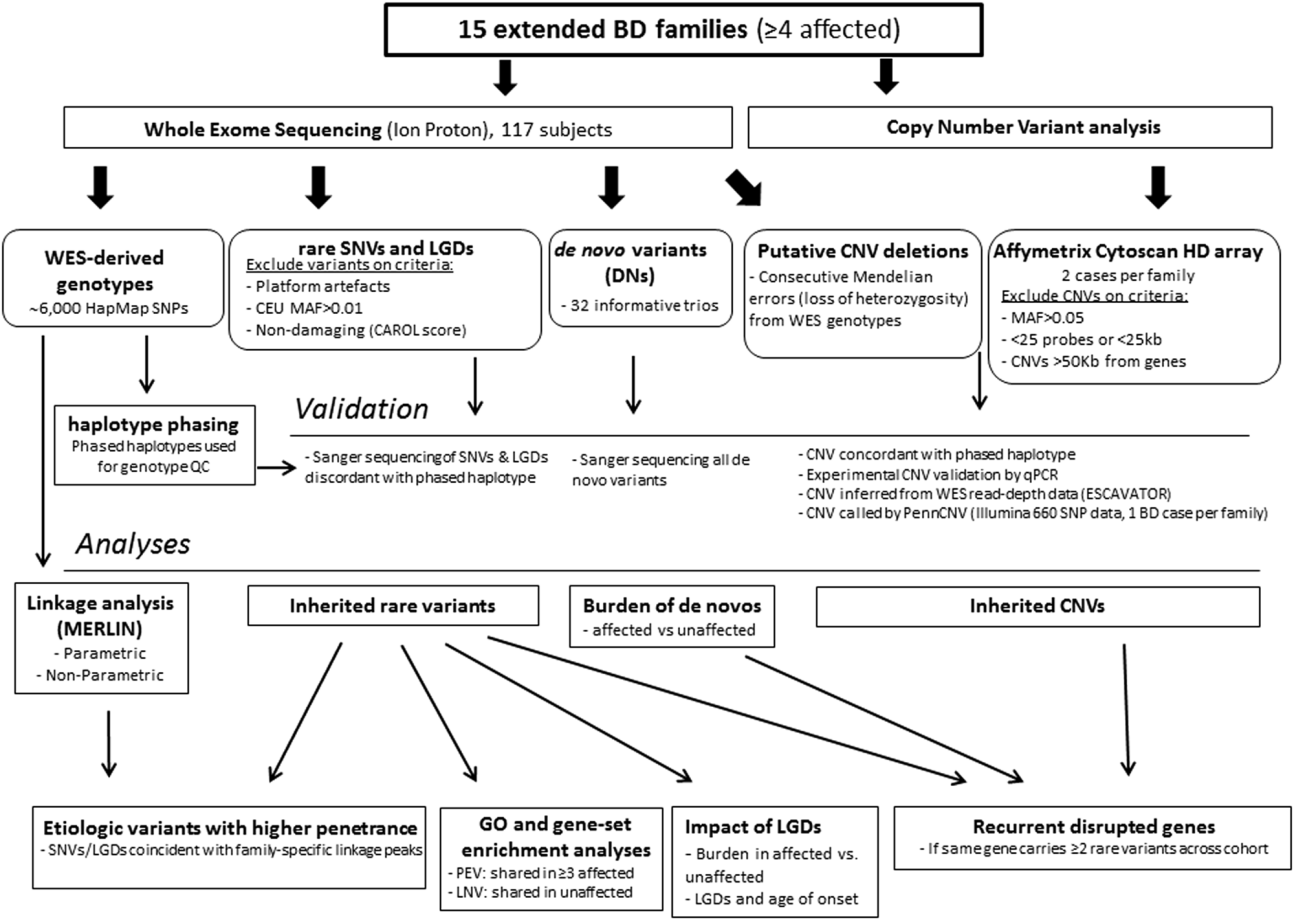
Workflow overview for the current study. Exclusion and inclusion filters and prioritization criteria for selection of the final pool of rare single-nucleotide variants (SNVs), copy number variants (CNVs) and de novo (DN) variants are described. WES whole-exome sequencing, NPL non-parametric linkage, QC quality control, LGDs likely gene-disruptive variants, GO gene ontology, PEV potentially etiologic variant, LNV likely neutral variant

WES was performed on 117 individuals from 15 Australian multiplex and extended BD families of European origin with a high density of illness, as defined by ≥4 relatives with BD-I, BD-II or SZMA (Fig. 1). Familial relationships between sequenced subjects were all confirmed at the sequence level using WES-derived genotypes and performing genome-wide IBD analysis (Supplementary Figure S1). Caucasian ethnicity was confirmed by multidimensional scaling analysis of genotype data, as shown in Figure S8. We used a comprehensive analysis approach, examining a range of rare variant classes for their potential role in familial BD: (i) selecting rare (MAF < 0.01) SNVs and likely gene-disruptive variants segregating with BD; (ii) identifying de novo variants; and (iii) examining CNVs (MAF < 0.05, within 50 kb from any coding gene) using combined analyses of CNV microarray and WES-based loss-of-heterozygosity and read depth data plus familial segregation inference. The workflow is summarized in Fig. 2.

### Selection of inherited rare SNVs and enrichment analysis

As penetrance of rare alleles and degree of polygenicity is heterogeneous for psychiatric disease, ranging from an oligogenic model comprising a small number of highly penetrant variants to a fully polygenic model comprising a larger number of less penetrant genes, we considered different degrees of rare variant sharing to identify potentially etiologic variants under both models. For each family, we considered two categories of rare variants: (1) SNVs or likely gene-disruptive variants shared in a minimum of three affected relatives and a maximum of one unaffected relative, which are potentially etiologic variants; and (2) SNVs shared in up to three unaffected relatives and a maximum of one affected relative, which are randomly shared, likely non-pathogenic variants, henceforth described as likely neutral variants. Under these models, 532 potentially etiologic variants (85% missense, 6.5% indel-frameshift, 5% nonsense, 3% splice-site and 0.5% start-lost) and 541 likely neutral variants (86% missense, 5% indel-frameshift, 4.5% nonsense, 4% splice-site and 0.5% start-lost) were identified (Supplementary Table S1).

To assess whether the pool of potentially etiologic variants were overrepresented in genes potentially involved in BD pathogenesis, we performed an enrichment analysis with gene sets previously implicated in studies of schizophrenia and BD^18,20,38^. The same analysis was performed with likely neutral variants as a control gene pool. Genes containing potentially etiologic variants were found to be enriched in the PSD^33^ (empirical *P* = 0.002, Bonferroni corrected *P* = 0.024), which was the only gene set showing significant enrichment after correction for gene length, genic tolerance and sequence coverage (Table 1). As expected, likely neutral variants were not enriched for any gene sets. GO analysis of potentially etiologic variants genes found interesting functions in the top enriched categories after correction for gene length, genic tolerance and sequence coverage (e.g., ligand-gated sodium channel activity, uncorrected empirical *P* = 0.0092), but none were significant after multiple testing correction (data available upon request).

**Table 1:** Enrichment analysis of predicted pathogenic rare variants shared in affected or unaffected relatives, after combining data across 15 Australian BD families

	PEVs (498 genes)		LNVs (495 genes)	
Gene set (*n *genes)	O (E)	*P*-val (corr *P*-val)	O (E)	*P*-value (corr *P-*val)
PSD (1445)	59 (41.5)	**0.002 (0.024)**	40 (38.5)	0.423 (1)
FMRP targets (837)	44 (39.6)	0.218 (1)	44 (38.2)	0.143 (1)
De novo PSY (1627)	71 (66.2)	0.254 (1)	67 (68.7)	0.627 (1)
NMDARs (61)	2 (1.7)	0.527 (1)	3 (2)	0.321 (1)
ARC (27)	0 (0.5)	1 (1)	1 (0.7)	0.541 (1)
Mitochondrial (1124)	22 (24.3)	0.727 (1)	21 (22.6)	0.679 (1)

Hypergeometic *p*-value, examining hits per category unadjusted for gene length, genic intolerance or sequence coverage of reference genes; AdjP-value, examining hits per category after adjustment for gene length, genic intolerance and sequence coverage of reference genes; AdjP-values that exceed Bonferroni correction for 12 independent tests are indicated in bold

*PEVs* potentially etiologic variants, which were shared in ≥3 affected relatives ± one unaffected relative (Supplementary Table S1), *LNVs* likely neutral variants were shared in 1 to 3 unaffected ± an affected relative (Supplementary Table S1), *O* number of genes observed in this category, *E* number of genes expected in this category, *PSD* genes expressed in the postsynaptic density^33^, De novo *PSYCH* de novo variants found in autism, schizophrenia and intellectual disability^20^, *NMDARs* *N*-methyl-d-aspartate (NMDA) receptor gene set^20^, *ARC* activity-regulated cytoskeleton-associated protein gene set^20^, *Mitochondrial* autosomal genes encoding mitochondrial localized proteins^35^

Although recessive rare variants were not expected (or observed) in these non-consanguineous families, we did observe compound heterozygous variants in two genes: two missense SNVs (rs200385024 and rs199673743) were observed in *HYDIN* gene in a single BD-I patient (PED_74-7400); and two frameshift-indels leading to a knock-out of *DNAH14* were observed in two affected relatives (PED_19-1917 and PED_19-1920, BD-I and SZMA, respectively) (Supplementary Figure S2).

### Impact of likely gene-disruptive SNVs on BD diagnosis and age of onset

To understand whether genome-wide burden of truncating variants plays a role in BD, we selected all likely gene-disruptive variants for each individual, independently of their familial segregation (*n* = 546 variants). Using a non-parametric test, we examined differences in the genome-wide burden of likely gene-disruptive variants between affected and unaffected relatives (Supplementary Figure S3). We found no significant difference in the total number of likely gene-disruptive variants between the two groups (mean ± SD = 13.1 ± 3.21 and 13.8 ± 3.17, respectively; Mann–Whitney *U-*test = 1361, two-tailed *P* = 0.24), nor when we restricted to those variants in brain-expressed genes (*P* = 0.17). Mixed-model analyses showed similar results (total, *P* = 0.33; brain expressed, *P* = 0.26). Furthermore, although underpowered for statistical analysis, of the 15 subjects with full GWAS data for whom polygenic risk scores (PRSs) could be calculated using previously identified common BD risk variants (most of which are noncoding), we observed no obvious relationship between the number of likely gene-disruptive variants and BD polygenic risk score (PRS in top 50% vs. bottom 50%: mean ± SD = 13.42 ± 4.61 vs. 13.12 ± 2.79, respectively; quintiles: mean ± SD = 14.75 ± 5.85 vs. 12.33 ± 3.06).

**Fig. 3:**
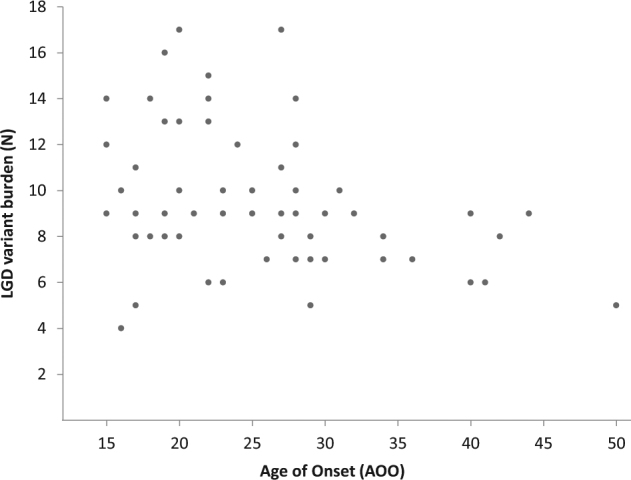
Higher burden of disruptive variants in brain-expressed genes relates to earlier age of onset. Correlation analysis was performed examining relationship between age of onset (AOO, *x *axis) and genome-wide burden of likely gene-disruptive (LGD) variants in brain-expressed genes (*y* axis). A total of 58 individuals affected with BD-I (*n* = 36), SZMA (*n* = 12), BD-II (*n* = 4) or RUD (*n* = 6), with typical age of onset (15–50 years) were considered

We next investigated whether earlier manifestation of symptoms correlated with an accumulation of likely gene-disruptive variants in brain-expressed genes. A Spearman’s correlation analysis was performed to examine the relationship between the number of likely gene-disruptive variants and age of onset. In 58 cases with broadly defined BD and typical onset (15–50 years)^2^, we found age of onset was negatively correlated with the number of brain-expressed likely gene-disruptive variants (*r*_s_(58) = –0.312; *P* = 0.017), with a higher burden of variants in patients with earlier symptom onset (Fig. 3). Similar findings were obtained when limiting analysis to patients with more severe forms of illness (BD-I/SZMA, *n* = 48; *r*_s_ = –0.325; *P* = 0.024).

### Role of inherited CNVs in familial BD

To assess whether structural variants contribute to risk of BD, we performed high-density array-based CNV analysis in two relatives per family, augmented with deletions from WES-derived genotype data. We then inferred familial segregation using phased haplotypes, and validated CNVs with SNP array data, read depth information and finally qPCR. We found 17 rare inherited CNVs in 15 loci across the genome (Supplementary Table S2). Three families harbored CNVs affecting the protocadherin alpha (PCDHA) gene cluster (Supplementary Figure S4), and two families with CNVs affecting the contiguous *PRODH* and *DGCR5* genes. Although not segregating closely with BD, we identified CNVs affecting intronic regions of candidate genes previously implicated in psychiatric disorders (i.e., *NLGN1*, *CNTNAP2*, *KCNB2* and *CNTN5)*, each in different families. These data provide additional corroborating evidence of their potential role in disease.

### Potentially etiologic SNVs and CNVs co-incident with linkage peaks

Linkage analysis using WES-derived genotypes may refine genomic intervals to identify putative highly penetrant etiologic variants. Thus, we performed parametric and non-parametric linkage for each family (Supplementary Information), and examined potentially etiological variants and CNVs, which coincide with family-specific linkage peaks to identify candidate genes for BD.

We found CNV duplications segregating with BD under the highest family-specific linkage peak in two families: one spanning *PDZD2* and *GOLPH3* (PED_5, hg19/chr5:32109541-32170613bp); the other spanning *AMICA1*, *MPZL3*, *MPZl2* and *CD3E* (PED_121, hg19/chr11:118081344-118195313bp) (Supplementary Table S2). The evidence of family-specific linkage at each SNV site is described in detail in Supplementary Material Section 3. In summary, the most promising etiological variants co-incident with family-specific linkage peaks (logarithm of the odds of linkage (LOD) ≥ 1.5) were: (1) a missense variant (rs61746994) in *TNS1* (PED_2, parametric LOD = 1.79); (2) an indel-frameshift variant (rs577236284) in the penultimate exon of *DLEC1* (PED_5, non-parametric ExLOD = 1.78); (3) a missense variant (rs9332239) in *CYP2C9* (PED_5, non-parametric ExLOD = 1.79); and finally, (4) a novel nonsense mutation (p.891 R > X, hg19/chrX:107976904 bp) in *IRS4* (PED_138, parametric LOD = 1.5) (Supplementary Figure S5A). The *IRS4* protein-truncating variant was genotyped by Sanger sequencing in all available family members, and found in all five affected siblings, as well as the youngest of three unaffected siblings (Supplementary Figure S5). Using immortalized lymphocyte cell lines from this family, we found that nonsense-mediated mRNA decay did not degrade the mutant transcript (Supplementary Figure S6).

### De novo variant load in familial BD

De novo variants may explain some of the missing heritability in sporadic cases^19^, but their impact in the context of familial BD is largely unexplored. Thus, we examined de novo variant load in 32 sequenced parent–child trios, which included 22 BD and 10 unaffected offspring (Supplementary Figure S7). We found 63 putative de novo variants, of which 45 (73%) validated by Sanger sequencing, including 31 coding variants (Supplementary Table S3) and 14 noncoding variants of unknown functional impact. We found no difference in the coding of de novo variant load between affected and unaffected offspring (*n* = 20/22 vs 11/10 coding variants; Mann–Whitney *U*-test *P* = 0.89, average of 0.97 observed per individual). Interestingly, we identified non-conservative de novo variants in a number of genes, including the *PC* gene (nuclear-encoded mitochondrial pyruvate carboxylase) (p.384Ala > Thr) and *MAP4* (microtubule-associated protein 4) (p.425Ile > Met), in a BD-I and SZMA patient respectively. We also identified variants in BD offspring, which included a nonsense de novo mutation in *BMP1*, and a missense predicted pathogenic variant in transcription factor *PHTF1*.

### Gene convergence across all variant classes

To identify potential candidate genes accumulating recurrent rare mutations across different variant classes, we examined genes harboring ≥2 putative damaging rare variants, which were shared in ≥3 affected relatives. These data were integrated with additional evidence for involvement in BD pathogenesis, including brain expression and gene-based evidence of common variant association with BD^6^ or schizophrenia^37^ as calculated using VEGAS2 (Table 2). Genes accumulating more evidence of putative involvement in BD were members of the protocadherin alpha cluster. Two genes, which are expressed in the PSD, also had recurrent mutations: the nuclear mitochondrial proline dehydrogenase gene *PRODH* and the extracellular matrix protein gene *TNC*.

**Table 2:** Recurrent SNV, CNV, de novo and likely gene-disruptive variations, found at least twice per gene

Gene	ExAC (z-score)	PED (*N*)	*N* variants (type)	***N*** aff (unaff) relatives	PGC1-BD VEGAS *P*	PGC2-SCZ VEGAS *P*
*ANGPTL5* ^*NB*^	−1.85	2	1 SNV (mis)	6 (2)	**0.045**	0.863
*DGCR5*	ND	2	2 CNVs (dup, del)	8 (2)	0.611	0.125
*DNAH14* ^*NB*^	−0.68	1	2 SNVs (fs)	4 (1)	0.201	0.338
*FAT2*	−1.01	2	2 SNVs (mis)	6 (2)	0.791	0.22
*HECTD4*	6.77	2	2 SNVs (mis)	7 (1)	0.367	0.146
*HYDIN*	ND	3	2 SNVs (mis)	9 (2)	0.651	**0.009**
*KIAA1731*	−0.18	2	2 SNVs (mis)	8 (1)	0.654	0.955
***MAP4***	−0.94	3	1 SNV (mis); 1 DN (mis)	5 (1)	0.61	**0.032**
*MIA3*	−1.43	2	2 SNVs (mis)	6 (0)	0.111	0.027
*MUC5B* ^*NB*^	6.3	5	6 SNVs (mis)	10 (8)	0.254	0.08
*NEB* ^*NB*^	−3.74	3	4 SNVs (mis)	9 (3)	0.098	**0.033**
*NRG1*	0.62	2	2 SNVs (mis)	6 (1)	0.232	0.47
*OBSCN*	−1.17	2	2 SNVs (mis)	6 (2)	0.947	0.289
*PCDHA8*	2.24	3	3 CNVs (del)	9 (1)	**0.022**	**6.10E-05**
*PCDHA9*	2.28	3	3 CNVs (del)	9 (1)	**0.02**	**7.20E-05**
*PCDHA10*	1.2	2	2 CNVs (del)	7 (1)	**0.023**	**8.30E-05**
*PCDH15*	−2.88	3	3 SNVs (mis)	9 (2)	0.101	**0.042**
***PRODH***	0.77	2	1 SNV; 2 CNVs (dup, del)	9 (3)	0.934	0.451
*PRUNE2*	−1.48	2	2 SNVs (mis)	7 (0)	0.427	0.728
*SCN10A* ^*NB*^	−1.8	2	4 SNVs (mis)	7 (4)	0.291	0.089
*SETX*	−1.62	2	2 SNVs (mis)	8 (1)	0.085	0.479
*SLC5A10*	0.23	1	2 SNVs (mis)	3 (1)	0.061	**0.002**
*SOGA1*	2.17	2	2 SNVs (mis)	7 (1)	0.358	0.089
***TNC***	−0.12	3	3 SNVs (mis)	8 (3)	0.367	0.055
*TTN* ^*NB*^	−4.93	7	13 SNVs (mis)	19 (7)	**0.014**	0.16
*VMAC*	0.81	2	2 SNVs (mis)	7 (1)	**0.002**	0.32
*XDH* ^*NB*^	−2.01	2	2 SNVs (mis)	6 (0)	0.466	0.434
*ZNF506*	0.09	2	2 SNVs (mis, fs)	6 (1)	0.43	0.221
*ZNF812* ^*NB*^	−4.76	2	2 SNVs (mis, fs)	7 (1)	0.851	0.639

For each gene, a measure of functional constraint in the form of ExAC missense z-score^36^ is provided. The number of pedigrees (PED *N*) with converging evidence for each gene is given, along with the number and type of variant identified (*SNV *single-nucleotide variant, *CNV *copy number variant, *DN* de novo variant, *mis *missense, *fs *frameshift, *dup *duplication, *del *deletion). The total number of affected relatives (*N* aff) and total number of unaffected relatives (*N* unaff) who carry a variant in the gene are indicated. Evidence of gene-level association with BD and schizophrenia was derived from summary statistics from Psychiatric Genomics Consortium GWAS^6,37^ with VEGAS2, where *p*-values < 0.05 are indicated in bold text. Postsynaptic density (PSD) gene names^33^ are indicated in bold. ^*NB*^, genes with negligible expression in the brain (RPKM < 1 in developmental transcriptomics RNA-seq data; http://www.brainspan.org/rnaseq/search/index.html). Further information on variants described above is reported in Supplementary Table S1, S2 and S3

## Discussion

Sequencing studies of extended BD families have recently suggested that rare variants contribute to the genetic architecture of this complex heritable condition^13–18^. To further elucidate novel genes and pathways important in disease risk, we performed WES in 15 extended and multiplex families with BD, selected for highly penetrant forms of illness. We extensively assessed the impact of inherited SNVs, likely gene-disruptive variants and CNVs, in conjunction with family-specific linkage, as well as examining the role of de novo variants in familial BD.

The pool of genes containing inherited single-nucleotide potentially etiologic variants, shared in three or more affected relatives, was enriched among those expressed in the PSD^33^, a structure with biological significance for BD. This enrichment was absent in the comparator likely neutral variant gene pool, potentially indicating specificity of enrichment to BD diagnosis. PSD regulates synaptic plasticity and crosstalk in glutamate-dopamine neurotransmission^39,40^. The PSD genes are extremely sequence conserved, and comprise scaffolding proteins, such as *SHANK3* and *NRXN1* genes, which are recurrently described with mutations in autism spectrum disorder and schizophrenia^41–43^. Another PSD gene, *ANK3*, is one of the most replicated associations for BD^6,44^. Furthermore, pathway analysis of common variants from GWAS data have implicated PSD genes as having a prominent role in schizophrenia and BD^45,46^. Therefore, our data add to growing evidence that cumulative pathogenic rare variants in several PSD genes may impair synaptic homeostasis, with effects across multiple psychiatric conditions.

Consistent with early linkage studies, we did not observe clear examples of variants of large effect that co-segregate precisely with BD. Taking the findings from the current and previous studies^13–18^, the most plausible genetic model would implicate the additive effect of both common and rare variants from a large number of genes, with risk alleles not fully penetrant and not perfectly segregating with disease. Futhermore, we see little relationship between polygenic risk score and the numbers of putative rare variants identified, although our analysis was limited to PRS from a single member of each family and thus only allowed for descriptive assessment of this relationship. As expected in non-consanguineous families, we found little evidence of rare recessive variants, but did observe interesting compound heterozygous likely gene-disruptive variants in *DNAH14*, although this gene is not highly brain expressed. Although recessive rare genotypes should not be discarded, they are unlikely to play a significant role in BD, as suggested from previous studies in schizophrenia^47–49^.

Recent sequencing studies in autism spectrum disorder showed a higher burden of de novo or inherited likely gene-disruptive variants both in trios or multiplex families^50–53^, and overall burden of severe gene-disruptive mutations has been correlated with disease severity^52^. The impact of likely gene-disruptive variant burden in severity of BD is relatively unexplored, but a study of 79 sporadic parent–child trios showed that increased gene-disruptive de novo variant load was associated with greater symptom severity and earlier age of onset of BD^19^. Although we found no difference in the total number of de novo variants between BD affected vs. unaffected offspring, a tendency toward a higher rate for predicted pathogenic de novo variants in affected offspring suggests that further studies are needed to understand the implications for de novo variants in BD, both in apparently sporadic and familial contexts. Our observed de novo rate (∼0.97) was in line with rates reported previously (∼0.89–0.94)^19,20^. Our very high sequence converge (120×) may have resulted in our de novo rate lying in the upper range of those previously reported values. We did not attempt to replicate the analysis of Kataoka et al.^19^ in regards to de novo variant load and symptom severity in BD, due to the low power of our sample (22 cases).

Interestingly, we observed convergence of evidence of involvement of *MAP4*, a PSD gene, which is also an FMRP target, for which de novo missense variant has previously been reported in ASD^20^. In *MAP4*, we observed both a missense de novo variant and an inherited pathogenic missense variant in patients with schizoaffective disorder. Another de novo variant was found in the *PC* gene, which maps to 1 of the 30 loci with significant BD association from the second Psychiatric Genomics Consortium GWAS (rs7122539, *p* = 3.80E-08)^54^, and produces a protein, which catalyzes the carboxylation of pyruvate to oxaloacetate, and is involved in insulin secretion and synthesis of the neurotransmitter glutamate.

Early age of onset is one of the clinical features often used to measure disease severity in BD and schizophrenia. Recent studies suggested that early-onset BD cases may be significantly influenced by genetics: increased risk of BD in offspring of parents with early onset^55^, and an overall heritability of 33% for age of onset in schizophrenia^56^. Intriguingly, we find that the burden of likely gene-disruptive variants in brain-expressed genes may exert age of onset effects, with higher numbers of disrupted genes among those with earlier onset. This finding is consistent with previous studies that correlated higher rates of CNVs, another class of gene-disruptive variants, with early age of onset in BD^8,9,11^. It is unclear whether brain-expressed likely gene-disruptive variants represent bona fide etiologic variants for BD, or whether a high burden of this variant class may reduce resilience (e.g., negatively impacting cognitive capacity or plasticity)^57^, resulting in anticipation.

CNVs represent another class of highly disrupting genomic variants considered herein. We did not examine de novo CNVs in our study, but our data do not support a model involving highly penetrant inherited CNVs with perfect disease segregation in any family, although we do find examples of potentially pathogenic CNVs in affected individuals, which may add to the genomic landscape of risk in BD.

When rare variants of all classes are combined with linkage evidence for each family, the most intriguing result was a protein-truncating mutation in the X-linked gene insulin receptor substrate 4 (*IRS4*) gene, found in PED_138. The *IRS4* mutation was present in all five siblings affected with BP-I or SZMA, as well as the youngest of three unaffected siblings. This may reflect reduced penetrance, although it should be noted that this unaffected *IRS4* p.R891X mutation carrier was aged 2 years younger than the average age of onset in this family at the time of her clinical assessment. Nonsense-mediated RNA decay prevents dominant-negative effects by degrading abnormal transcripts with premature stop mutations^58^, although single exon genes, like *IRS4*, are expected to escape nonsense-mediated decay^59^—which we experimentally confirmed. Although there is little evidence that common variants on the X chromosome are strongly associated with BD^60^, the contribution of rare X-linked variants has been poorly studied. Several lines of evidence suggest that *IRS4* may be a novel candidate for BD: (i)* IRS4* is highly conserved, and truncating mutations extremely rare (freq = 4.6E-05; ExAC); (ii)* IRS4* expression in the brain is sexually dimorphic^61^, and largely restricted to the amygdala and ventral hypothalamus^62,63^, regions responsible for emotional regulation, fear response and nurturing behaviors; (iii) IRS4 stimulates phosphatidylinositol 3 kinase/AKT pathway signaling and interacts with ErbB2^64^, a gene significantly associated with BD^60^; and (iv) insulin-related signaling regulating postsynaptic spine formation has been implicated in obsessive-compulsive disorder^65^, a psychiatric condition with shared genetic contributors to BD and schizophrenia^66^. When knocked out, female *IRS4*^-/-^ mice show reduced nurturing behavior^61,67^, although extensive behavioral, neuroanatomical and pharmacological response assessments are yet to be made.

Finally, we observed several rare recurrent variations in two interesting loci relevant to BD: the protocadherin alpha (*PCDHA*) gene cluster, and proline dehydrogenase 1 (*PRODH)*. The *PCDHA* cluster, expressed in serotonergic neurons^68^, lies in a 200 kb LD block, which was significantly associated with schizophrenia in the Psychiatric Genomics Consortium GWAS of 36,989 cases and 113,075 controls (max SNP-based *p* = 4.85E-08)^37^. Futhermore, several PCDHA members are regulated by miR-1908, a microRNA that was independently associated with BD^69^, and may suggest functional convergence. *PRODH* encodes a nuclear-encoded mitochondrial protein, involved in the catabolism of proline to l-glutamate, which acts as neuromodulator of dopaminergic synaptic transmission. CNVs spanning *PRODH* are significantly associated with schizophrenia^12^, and microdeletions including *PRODH* at 22q11.2 are associated with DiGeorge syndrome^70^, in which ~30% of patients develop psychotic symptoms and 25% develop schizophrenia^71^. Furthermore, common functional variants in *PRODH* have been associated with alterations in prefrontal–striatal brain circuits affecting working memory and cognitive gating^72^. Therefore, our study provides corroborating evidence of putative involvement of *PRODH* in BD.

In conclusion, genetic approaches that combine WES-derived SNV, CNV and linkage analyses in extended families are effective in pinpointing genes and pathways that may contribute to the pathophysiology of BD. Our results show rare putatively functional variants in genes previously implicated by GWAS, as well as novel genes whose role in BD is yet to be fully elucidated.

## Electronic supplementary material

Supplementary Material
