# Supplementary material for: An examination of multiple classes of rare variants in extended families with bipolar disorder
Source: Transl Psychiatry. 2018 Mar 13;8:65. doi: 10.1038/s41398-018-0113-y (PMC5847564; doi:10.1038/s41398-018-0113-y)
Supplement: Supplementary file 1 — Supplementary Material [file 41398_2018_113_MOESM1_ESM.docx]

**Supplementary information:**

**An Examination of Multiple Classes of Rare Variants in Extended Families with Bipolar Disorder**

Claudio Toma, Alex D. Shaw, Richard J. N. Allcock, Anna Heath, Kerrie D. Pierce, Philip B. Mitchell, Peter R. Schofield, Janice M. Fullerton

**This file includes:**

1. **Clinical assessments**
2. **Multidimensional Scaling, genetic relatedness (pairwise-IBD) and polygenic risk score (PRS) analyses**
3. **Linkage analysis and SNV co-localisation for each BD family**
4. **CNV studies**
5. **Statistical methods and procedures**
6. **De novo variant study**
7. **Supplementary References**
8. **Supplementary Tables (1 to 3)**
9. **Supplementary Figures (1 to 8)**
10. **CLINICAL ASSESSMENTS**

Medium to large multiplex and extended multigenerational pedigrees were collected through the Mood Disorders Unit and Black Dog Institute at the Prince of Wales Hospital, and the School of Psychiatry, University of New South Wales, Sydney, Australia.^1-5^ All families were ascertained after initial consultation with a proband with bipolar disorder type I (BD-I), using the Family Interview for Genetic Studies (FIGS).^6^ Each pedigree member provided peripheral blood samples for DNA extraction by standard laboratory methods. All individuals included for sequencing were assessed using the Diagnostic Interview for Genetic Studies (DIGS),^7^ with interviews being undertaken by experienced medical practitioners, psychologists and psychiatric nurses trained in this instrument. Information obtained from the FIGS, DIGS and medical records was used to generate best-estimate Research Diagnostic Criteria (RDC) for DSM-IV^8^ bipolar disorder type I (BD-I), bipolar disorder type II (BD-II), schizoaffective disorder-manic type (SZMA) or recurrent unipolar depression (RUD).

Age of onset (AOO) is considered as age of first presentation of a clean episode of any mood disorder (manic or depressive). For unaffected subjects who were sequenced, a broad liability class is given reflecting their age at clinical assessment. Based on typical age of onset for BD, unaffected subjects who were more than 40 years of age at diagnostic interview are likely to remain unaffected. For a small number of subjects who were unaffected, diagnostic interview was conducted at 20-39 years, thus may be a less reliable phenotype (PED_11-1118, PED_18-1810, PED_84-8411, PED_138-20105, PED_138-20110, PED_138-20097, PED_138-20104; PED_5-523, PED_19-1918, PED_113-11308, PED_198-21832)

1. **Multidimentional Scaling (MDS), GENETIC RELATEDNESS (pairwise IBD) AND polygenic risk score (PRS) ANALYSES**

The ethnicity for each family was determined to be of European descent, based on: i) ancestral self-report from demographic information supplied at clinical interview; and ii) genotype-derived ethnicity from genome-wide SNP data for at least one individual for each family. Multidimensional scaling (MDS) analysis was performed in PLINK^9^ using genotypes from Illumina 660quad chip array,^10-12^ following the ENIGMA2 protocols (http://enigma.ini.usc.edu/). In brief, post-QC genotype files (n=348 case subjects) were merged with genotypes from 1000 genomes samples (Human Mapping set 3), using SNPs which are polymorphic in European populations. SNPs with minor allele frequency (MAF)<0.01, genotype call rate <95%, strand ambiguity, and those with HWE p<1e-06 were excluded prior to MDS analysis, and the values from first two principle components were used to ascribe genotype-derived ethnicity. Principle components values for HAPMAP Caucasian European (CEU) subjects ranged between -0.0511 to -0.0574 for PCA-C1, and -0.0545 to -0.0463 for PCA-C2. Plots showing the relative position of each family member relative to HAPMAP reference individuals is given in Supplementary Figure 8.

Polygenic risk scores (PRS) were determined using common SNPs (MAF>0.05) with nominal disease association from the Psychiatric Genomics Consortium GWAS of 7,481 BD cases and 9,250 controls^12^. Risk scores were calculated in PLINK^9^ for SNPs which exceeded a p-value threshold (P_T_)<0.2, and scores were weighted by the logarithm of the odds ratio for disease association. The percent rank of PRS score for individuals from each of the 15 sequenced families were determined by comparison against the PRS score for all other Australian case subjects from the Moods cohort^10^. Probands from families with ‘high’ PRS were individuals PED_17-1720, PED_113-11300 and PED_129-12902, which fell in top 15-20% of PRS at P_T=0.2_, while individual PED_198-21805 fell in top 30%. Probands from families with ‘moderate-average’ PRS were individuals, PED_5-518, PED_19-1917, PED_74-7400 and PED_138-20111, whose PRS fell in top 50% of PRS at P_T=0.2_. Probands from families with ‘low-average’ PRS were individuals PED_2-207, PED_11-1100, PED_131-13101 and PED_121-12109, whose PRS fell in bottom 50% of PRS at P_T=0.2_, while individuals PED_84-8402 and PED_1-63 fell in the bottom 20%, and individual PED_18-1808 fell in bottom 5% of PRS at P_T=0.2_.

Familial relationships between sequenced subjects were confirmed using WES-derived genotypes and performing genome-wide Identity By Descent (IBD) analysis in PLINK^9^ (Supplementary Figure 1).

**Ethics approval**

The study was approved by the Human Research Ethics Committee of the University of New South Wales, and written informed consent was obtained from all participating individuals.

1. **LINKAGE ANALYSIS**

Linkage studies were performed under two parametric and two non-parametric models using approximately 6,000 SNPs for each family. Parametric LOD scores (HLOD) were calculated under a dominant model considering fully penetrant (penetrance 1) and a partially penetrant (penetrance 0.6) risk allele, modelling a risk allele frequency of 1% and phenocopy rate of 0.1%. Non-parametric linkage (NPL) analyses were performed using the “all” statistic implemented in Merlin, under the Kong and Cox linear (LOD) and exponential (ExLOD) model. Linkage analysis results are plotted for each of the 15 BD families below. For each family, the pedigree structure (A) and linkage plots (B) are shown, where NPL linear model is in black; NPL exponential model is in red; parametric linkage under 100% and 60% penetrance dominant models are in green and blue respectively. Symbols in the pedigree figures below follow the legend in Figure 1.

Patients with recurrent unipolar disorder (RUD) with no affected descendants (n=8) were considered as phenotypically unknown, due to the high frequency of depression in the general population. The ID of these individuals are PED_1-50 and PED_1-25, PED_19-1926, PED_129-12901, PED_113-11306, PED_131-13103, PED_131-13111 and PED_131-13115. In PED_1, the biggest pedigree from our collection, we considered unaffected those individuals with unaffected offspring only (individuals PED_1-20, PED_1-22 and PED_1-28). Subject PED_1-65, who had no offspring, was considered unknown. In each family below, we list those predicted pathogenic variants which were shared in at least 3 affected relatives, and which coincide with family-specific linkage peaks (LOD>0.5, under any linkage model considered in our study). Further detailed information on each of these variants are provided in Supplementary Table 1.

**BIPOLAR_FAMILY­_ID: PED_1**

**A
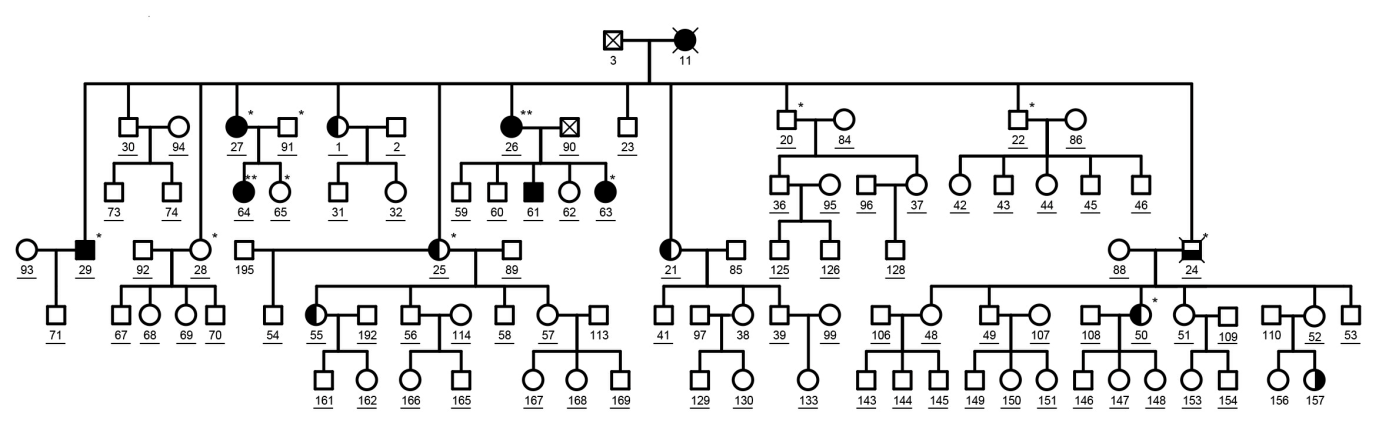
**

**B**

**
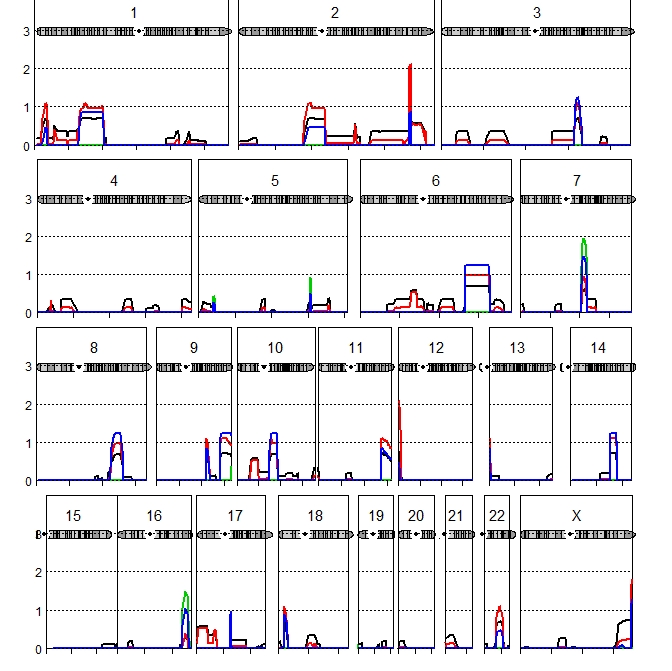
**

| **Chr:position** | **LOD** | **ExLOD** | **LOD_dom** | **LOD_0.6** | **Ref/Alt** | **Gene** | **AA change** | **dbSNP_ID** |
| --- | --- | --- | --- | --- | --- | --- | --- | --- |
| 8:105502966 | 0.599 | 0.834 | -3.3033 | 1.1052 | C/T | LRP12 | p.820D>N | rs150116835 |
|  |  |  |  |  |  |  | p.839D>N |  |
| 8:113649070 | 0.687 | 0.971 | -9.5104 | 1.2445 | T/C | CSMD3 | p.1127S>G | rs765216700 |
|  |  |  |  |  |  |  | p.1231S>G |  |
|  |  |  |  |  |  |  | p.1191S>G |  |
| 17:41243948 | 0.373 | 0.365 | -2.8861 | 0.6109 | C/A | BRCA1 | p.1200Q>H | rs56214134 |
|  |  |  |  |  |  |  | p.1153Q>H |  |

**BIPOLAR_FAMILY­_ID: PED_2**

**
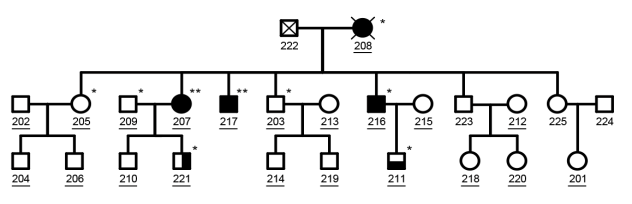

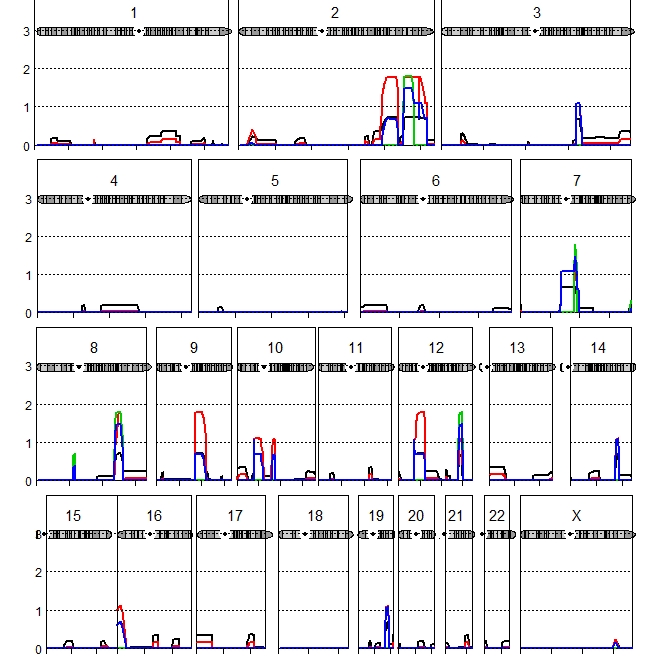
**

**A B**

| **Chr:position** | **LOD** | **ExLOD** | **LOD_dom** | **LOD_0.6** | **Ref/Alt** | **Gene** | **AA change** | **dbSNP_ID** |
| --- | --- | --- | --- | --- | --- | --- | --- | --- |
| 2:218712290 | 0.716 | 1.777 | 1.7957 | 1.476 | G/A | TNS1 | p.859R>W | rs61746994 |
| 2:226378176 | 0.716 | 1.778 | 1.7957 | 1.476 | A/T | NYAP2 | p.104D>V | rs755957714 |
| 9:89771533 | 0.716 | 1.777 | -8.9236 | 0.6886 | C/T | C9orf170 | p.72Q>* | / |

**BIPOLAR_FAMILY­_ID: PED_5**

**
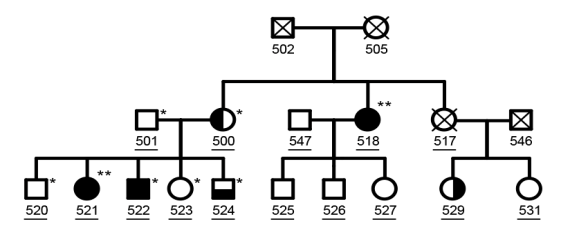

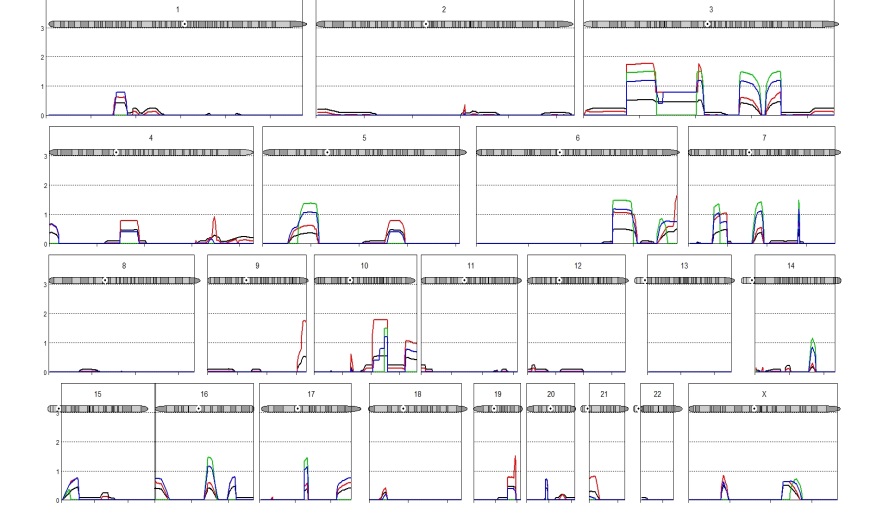
**

**A B**

| **Chr:position** | **LOD** | **ExLOD** | **LOD_dom** | **LOD_0.6** | **Ref/Alt** | **Gene** | **AA change** | **dbSNP_ID** |
| --- | --- | --- | --- | --- | --- | --- | --- | --- |
| 3:38163606-7 | 0.535 | 1.78 | 1.4968 | 1.1906 | -/CAAA | DLEC1 | indel, fs | rs577236284 |
| 6:130166999 | 0.491 | 1.068 | 1.4826 | 1.1761 | T/C | TMEM244 | indel, fs | rs146932263 |
| 9:139731827 | 0.521 | 1.748 | -6.2997 | 0.7769 | G/A | RABL6 | p.281R>H | rs200704265 |
|  |  |  |  |  |  |  | p.280R>H |  |
| 9:140120188 | 0.52 | 1.745 | -11.2271 | 0.7761 | T/A | C9orf169 | p.39S>T | rs182088466 |
| 10:96748777 | 0.535 | 1.78 | -11.5065 | 0.7951 | C/T | CYP2C9 | p.489P>S | rs9332239 |
| 10:134017305 | 0.422 | 0.972 | -10.3876 | 0.6807 | G/A | DPYSL4 | p.501G>R | rs61865802 |
| 16:2259410 | 0.391 | 0.57 | -3.321 | 0.7371 | G/A | MLST8 | p.214P>S | rs111384111 |
| 17:80016270 | 0.405 | 0.588 | -2.3364 | 0.7553 | A/T | DUS1L | p.410L>Q | / |

**BIPOLAR_FAMILY­_ID: PED_11
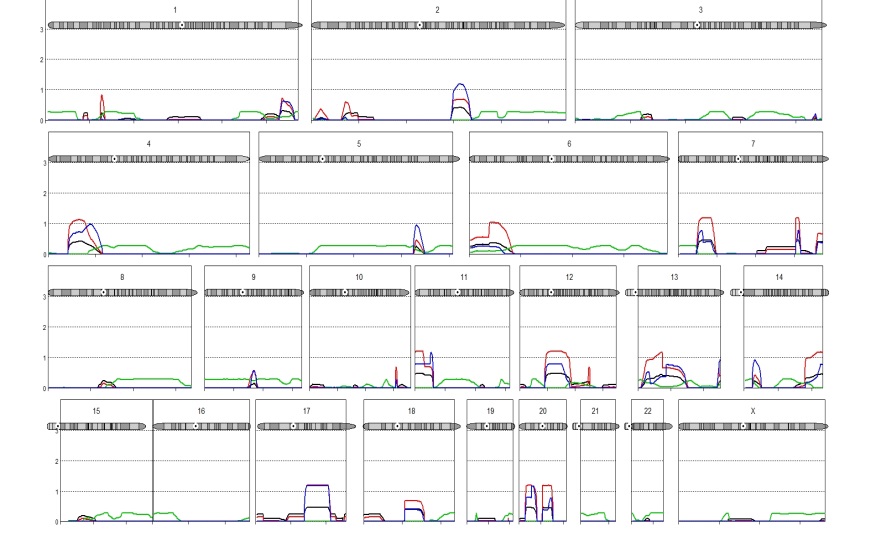
**

**
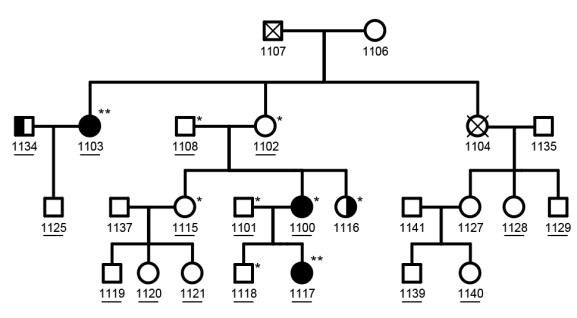
**

**A B**

| **Chr:position** | **LOD** | **ExLOD** | **LOD_dom** | **LOD_0.6** | **Ref/Alt** | **Gene** | **AA change** | **dbSNP_ID** |
| --- | --- | --- | --- | --- | --- | --- | --- | --- |
| 12:48597058-9 | 0.462 | 1.193 | -1.0363 | 0.7864 | -/CTGC | OR10AD1 | indel, fs | rs754837553 |
| 12:52681822 | 0.462 | 1.193 | -1.0362 | 0.7864 | A/T | KRT81 | p.282Y>* | rs138597671 |

**BIPOLAR_FAMILY­_ID: PED_17**

**
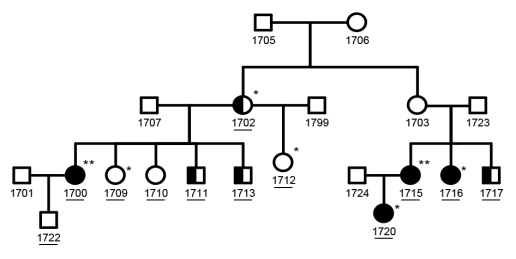

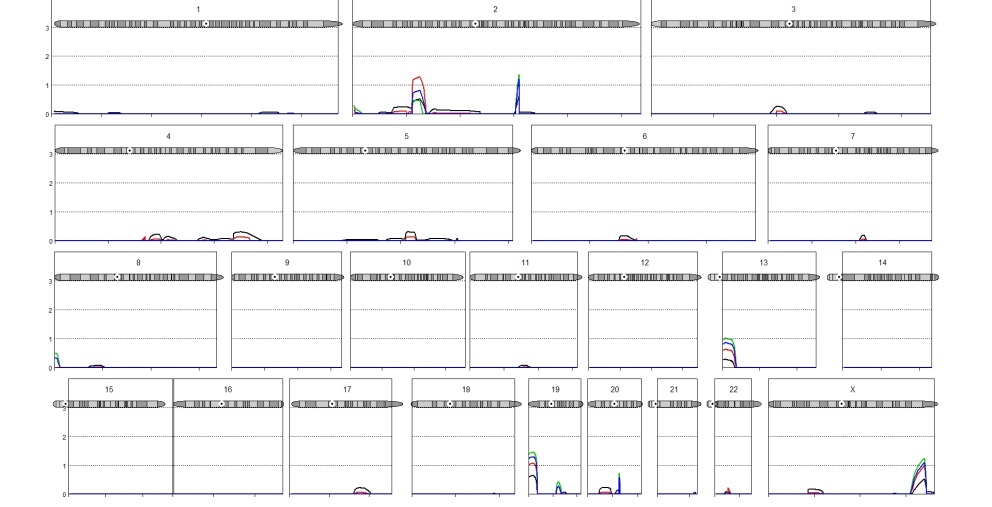
**

**A B**

**BIPOLAR_FAMILY­_ID: PED_18**

**
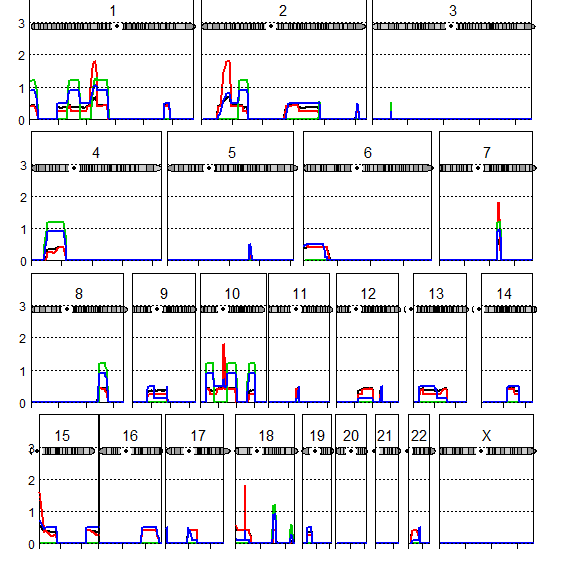
**

**
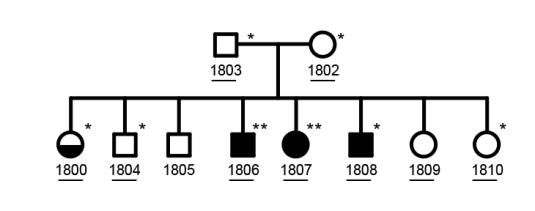
A B**

| **Chr:position** | **LOD** | **ExLOD** | **LOD_dom** | **LOD_0.6** | **Ref/Alt** | **Gene** | **AA change** | **dbSNP_ID** |
| --- | --- | --- | --- | --- | --- | --- | --- | --- |
| 1:3551827 | 0.432 | 0.417 | 1.2015 | 0.9028 | C/T | WRAP73 | p.212R>Q | rs776582305 |
| 1:36935300 | 0.356 | 0.248 | -2.9452 | 0.5068 | G/C | CSF3R | p.476T>S | rs374610386 |
| 1:47571862 | 0.356 | 0.248 | 1.2019 | 0.9028 | C/G | CYP4Z1 | p.377A>G | rs138601503 |
| 2:27308854 | 0.432 | 0.417 | -2.6658 | 0.5074 | T/C | EMILIN1 | p.1008Y>H | / |
| 10:24833063 | 0.366 | 0.27 | -3.0942 | 0.5068 | C/T | KIAA1217 | p.1622R>C | / |
| 10:52005095 | 0.432 | 0.418 | 1.0061 | 0.797 | G/A | ASAH2 | p.83Q>* | rs116049719 |
| 10:55581787 | 0.431 | 0.418 | 1.2013 | 0.9028 | T/A | PCDH15 | p.1907Y>F | / |
|  |  |  |  |  |  |  | p.1902Y>F |  |
|  |  |  |  |  |  |  | p.1831Y>F |  |
|  |  |  |  |  |  |  | p.1897Y>F |  |
|  |  |  |  |  |  |  | p.1860Y>F |  |
|  |  |  |  |  |  |  | p.1880Y>F |  |
|  |  |  |  |  |  |  | p.1877Y>F |  |
|  |  |  |  |  |  |  | p.1900Y>F |  |
| 14:68040543 | 0.432 | 0.418 | -2.8068 | 0.5075 | G/T | PLEKHH1 | p.622G>V | / |
| 16:81078202 | 0.432 | 0.417 | -3.0124 | 0.5075 | T/C | ATMIN | p.700F>S | / |

**BIPOLAR_FAMILY­_ID: PED_19**

**
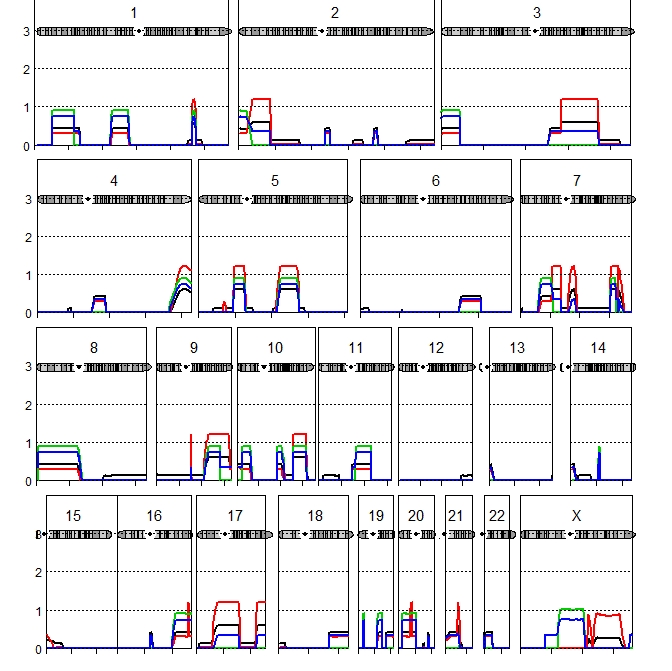
**

**A B**

**
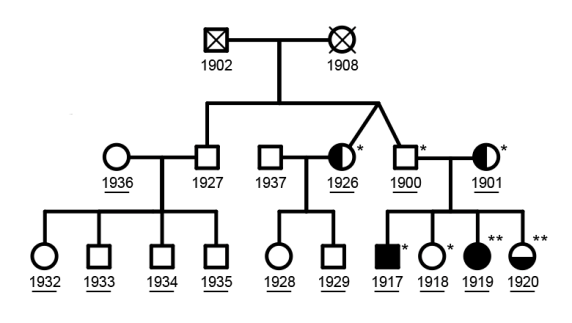
**

| **Chr:position** | **LOD** | **ExLOD** | **LOD_dom** | **LOD_0.6** | **Ref/Alt** | **Gene** | **AA change** | **dbSNP_ID** |
| --- | --- | --- | --- | --- | --- | --- | --- | --- |
| 1:13910346 | 0.426 | 0.301 | 0.9016 | 0.7394 | C/T | PDPN | p.16R>W | rs113350533 |
| 1:18961618 | 0.426 | 0.301 | 0.9017 | 0.7396 | C/T | PAX7 | p.112P>L | rs142754204 |
| 1:92649625 | 0.426 | 0.301 | 0.9014 | 0.7392 | C/T | KIAA1107 | p.1268S>F | / |
| 1:109734349 | 0.426 | 0.302 | 0.9015 | 0.7394 | G/A | KIAA1324 | p.516G>D | rs41279690 |
| 3:142285014 | 0.602 | 1.204 | -3.2163 | 0.3477 | G/A | ATR | p.81L>F | rs147353060 |
| 3:151171483 | 0.602 | 1.203 | -2.8872 | 0.3477 | T/G | IGSF10 | p.135H>P | rs143943953 |
| 3:169099213 | 0.602 | 1.204 | -3.0225 | 0.3507 | G/A | MECOM | p.46P>L | rs376185183 |
| 4:186545234 | 0.602 | 1.204 | 0.9017 | 0.7403 | G/A | SORBS2 | p.350A>V | rs773054747 |
|  |  |  |  |  |  |  | p.446A>V |  |
| 5:54558510 | 0.328 | 0.678 | 0.3765 | 0.2264 | G/A | DHX29 | p.1259S>F | rs147947389 |
| 7:36338668 | 0.602 | 1.204 | -8.0572 | 0.3508 | C/G | EEPD1 | p.521N>K | rs143061882 |
| 7:65444841 | 0.275 | 0.518 | -2.4932 | -0.2909 | C/T | GUSB | p.152D>N | rs149606212 |
| 7:142880936 | 0.602 | 1.204 | 0.9018 | 0.7406 | T/C | TAS2R39 | p.142F>S | rs200343853 |
| 8:16012590 | 0.425 | 0.301 | 0.9003 | 0.7383 | C/T | MSR1 | p.294G>E | rs41440349 |
| 9:108337380 | 0.419 | 0.888 | 0.5835 | 0.4286 | T/C | FKTN | p.23F>L | / |
| 9:111624712 | 0.507 | 1.052 | 0.7491 | 0.5909 | C/T | ACTL7A | p.37P>L | rs149755351 |
| 9:117788933 | 0.602 | 1.204 | 0.9018 | 0.7406 | C/T | TNC | p.2071E>K | rs140573419 |
| 9:135202325 | 0.602 | 1.204 | -3.7874 | 0.3507 | A/C | SETX | p.1554C>G | rs112089123 |
| 14:58838672 | 0.284 | 0.179 | 0.7588 | 0.5981 | C/G | ARID4A | p.1247P>A | rs750070120 |
| 16:88498350 | 0.424 | 0.3 | 0.8997 | 0.7378 | C/T | ZNF469 | p.1463T>M | rs375045076 |
| 17:18862551 | 0.602 | 1.204 | -2.9748 | 0.3477 | A/T | SLC5A10 | p.96N>I | rs148178887 |
| 17:18881091 | 0.602 | 1.204 | -3.1375 | 0.3477 | G/A | SLC5A10 | p.630R>W | rs201046878 |
| 17:39316732 | 0.602 | 1.204 | -8.0572 | 0.3478 | C/A | KRTAP4-4 | p.71C>F | / |
| 17:76993649 | 0.601 | 1.201 | -1.9631 | 0.3469 | C/T | CANT1 | p.19R>Q | rs144060377 |
| 19:45667499 | 0.426 | 0.301 | 0.9018 | 0.7396 | A/T | TRAPPC6A | p.107Y>N | rs142501705 |
| 20:35414944 | 0.426 | 0.301 | 0.9018 | 0.7396 | G/A | SOGA1 | p.1644H>Y | rs199819154 |

**BIPOLAR_FAMILY­_ID: PED_74**

**
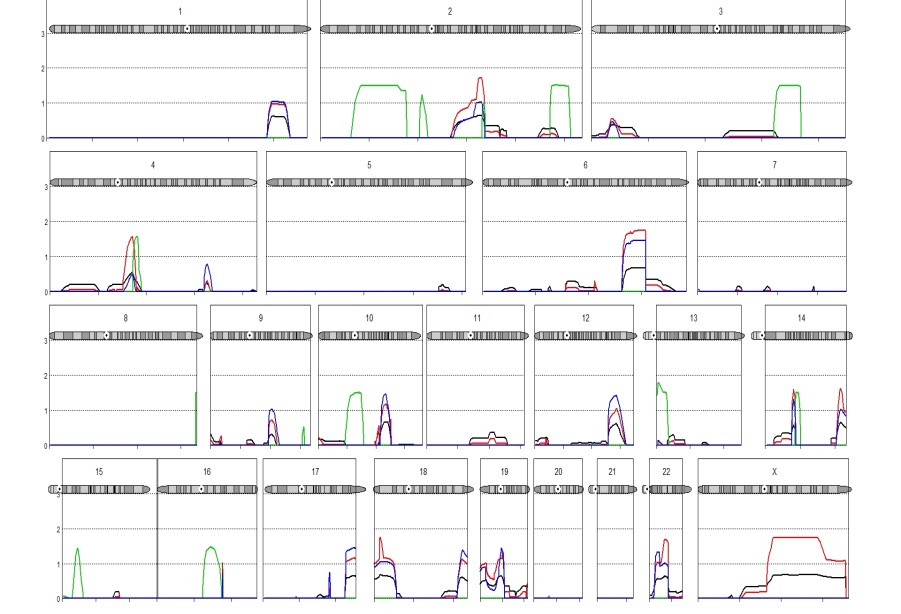
**

**
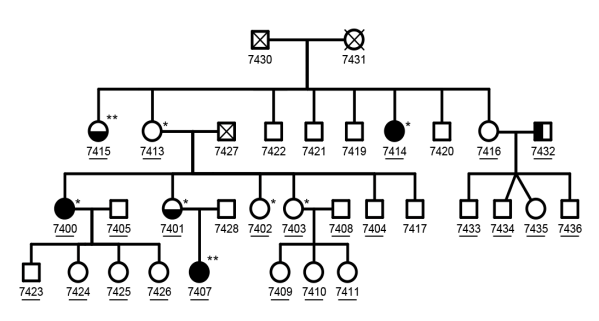
A B**

| **Chr:position** | **LOD** | **ExLOD** | **LOD_dom** | **LOD_0.6** | **Ref/Alt** | **Gene** | **AA change** | **dbSNP_ID** |
| --- | --- | --- | --- | --- | --- | --- | --- | --- |
| 12:121448918 | 0.601 | 1.038 | -4.1497 | 1.3939 | T/A | C12orf43 | p.59Q>H | rs112728225 |
| 19:21991987-8 | 0.643 | 1.146 | -6.6013 | 1.3473 | TT/- | ZNF43 | indel, fs | rs761612770 |
| 19:23542632 | 0.644 | 1.148 | -6.2275 | 1.4399 | T/C | ZNF91 | p.1050Y>C | rs202182741 |
| 2:31605868 | -0.053 | -0.08 | 1.499 | -0.1516 | G/A | XDH | p.346A>V | rs142388231 |
| 2:152382725 | 0.365 | 0.427 | 0.8052 | 0.0526 | G/A | NEB | p.7333P>S | / |
| 2:232393323 | -0.006 | -0.001 | 1.4988 | -0.1502 | G/A | NMUR1 | p.137R>C | rs146282412 |
| 3:155547508 | -0.006 | -0.001 | 1.4982 | -0.1507 | T/G | SLC33A1 | p.484N>T | rs144015992 |
| 3:167083673 | -0.006 | -0.001 | 1.4987 | -0.1503 | C/G | ZBBX | splicing | rs184708397 |
| 16:70954909 | 0.138 | 1.028 | -0.8484 | 0.7364 | A/G | HYDIN | p.2456F>S | rs200385024 |
| 16:71101211 | 0.064 | 0.87 | -0.6917 | 0.5804 | G/A | HYDIN | p.713A>V | rs199673743 |
|  |  |  |  |  |  |  | p.703A>V |  |
|  |  |  |  |  |  |  | p.686A>V |  |

**BIPOLAR_FAMILY­_ID: PED_84**

**
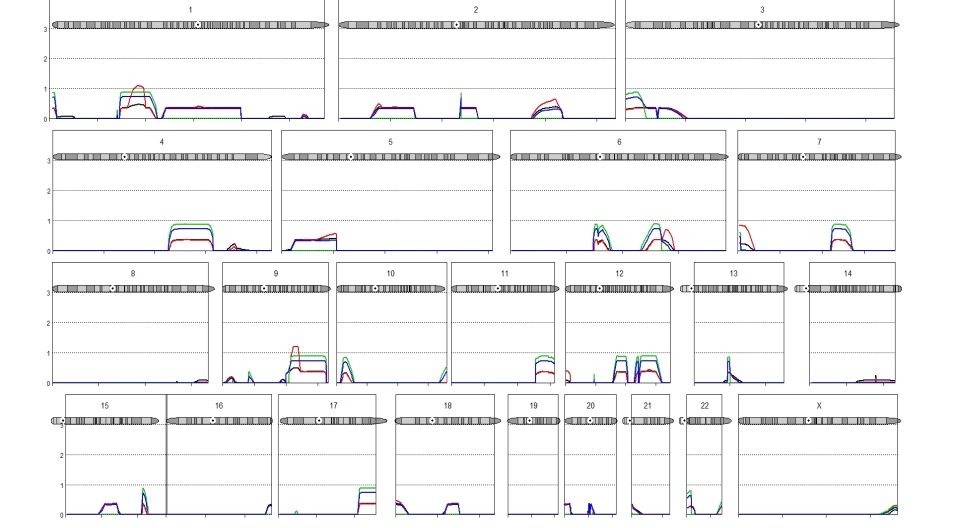
**

**
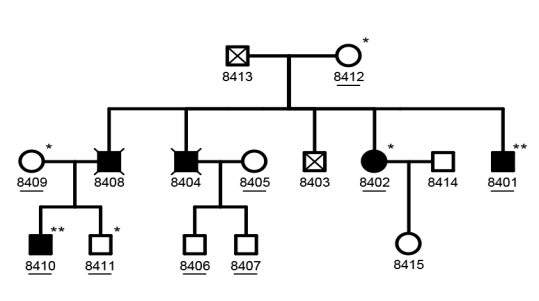
A B**

| **Chr:position** | **LOD** | **ExLOD** | **LOD_dom** | **LOD_0.6** | **Ref/Alt** | **Gene** | **AA change** | **dbSNP_ID** |
| --- | --- | --- | --- | --- | --- | --- | --- | --- |
| 10:5791299 | 0.314 | 0.3 | 0.8242 | 0.6718 | A/G | FAM208B | p.1972Q>R | rs200658639 |
| 17:73089805 | 0.368 | 0.356 | 0.8806 | 0.7282 | G/A | SLC16A5 | p.25G>D | / |

**BIPOLAR_FAMILY­_ID: PED_113**

**
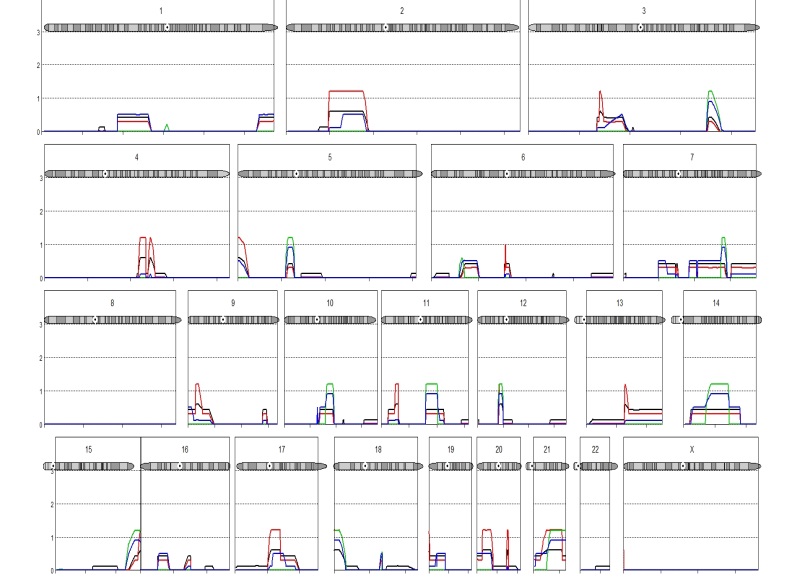
**

**A B**

**
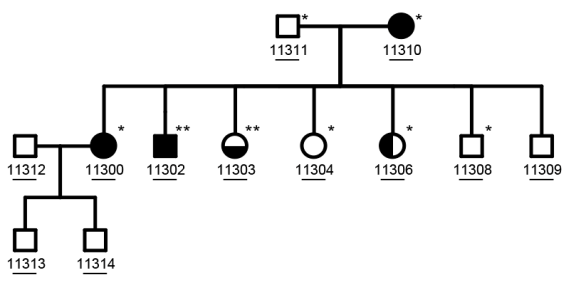
**

| **Chr:position** | **LOD** | **ExLOD** | **LOD_dom** | **LOD_0.6** | **Ref/Alt** | **Gene** | **AA change** | **dbSNP_ID** |
| --- | --- | --- | --- | --- | --- | --- | --- | --- |
| 1:89414776 | 0.426 | 0.301 | -2.2221 | 0.5055 | A/T | CCBL2 | p.380L>Q | rs144984854 |
|  |  |  |  |  |  |  | p.346L>Q |  |
| 2:53955867 | 0.602 | 1.204 | -7.8998 | 0.5061 | T/C | ASB3 | p.234S>G | rs75729621 |
|  |  |  |  |  |  |  | p.123S>G |  |
|  |  |  |  |  |  |  | p.196S>G |  |
| 2:54093975 | 0.602 | 1.204 | -7.8998 | 0.5061 | C/T | PSME4 | p.1769C>Y | / |
| 2:58388714 | 0.601 | 1.202 | -7.8998 | 0.5061 | A/T | FANCL | p.326D>E | rs140088149 |
|  |  |  |  |  |  |  | p.321D>E |  |
| 5:637730 | 0.597 | 1.197 | -1.0593 | 0.5013 | C/T | CEP72 | p.335R>* | / |
| 10:50681043 | 0.426 | 0.301 | -4.1168 | 0.5058 | G/A | ERCC6 | p.914T>M | rs142580756 |
| 11:93463070 | 0.426 | 0.301 | 1.2008 | 0.902 | A/T | KIAA1731 | p.2534K>N | rs183120281 |
| 14:74522351 | 0.426 | 0.301 | 1.2012 | 0.9016 | C/T | C14orf45 | p.451R>* | rs372260972 |
| 17:37900378 | 0.602 | 1.204 | -7.8998 | 0.5075 | G/T | GRB7 | p.240G>V | rs145986585 |
|  |  |  |  |  |  |  | p.263G>V |  |
| 17:39037017 | 0.602 | 1.204 | -7.7016 | 0.5075 | T/C | KRT20 | p.160E>G | rs148091209 |
| 19:467664 | 0.556 | 1.097 | -2.0519 | 0.1126 | G/A | ODF3L2 | p.112R>W | rs79399119 |
| 19:10748382 | 0.426 | 0.301 | -7.0098 | 0.5058 | G/C | SLC44A2 | p.550E>Q | rs142741358 |
|  |  |  |  |  |  |  | p.552E>Q |  |
| 20:2597716 | 0.426 | 0.301 | -7.8998 | 0.5053 | A/T | TMC2 | splicing | rs140053393 |

**BIPOLAR_FAMILY­_ID: PED_121**

**
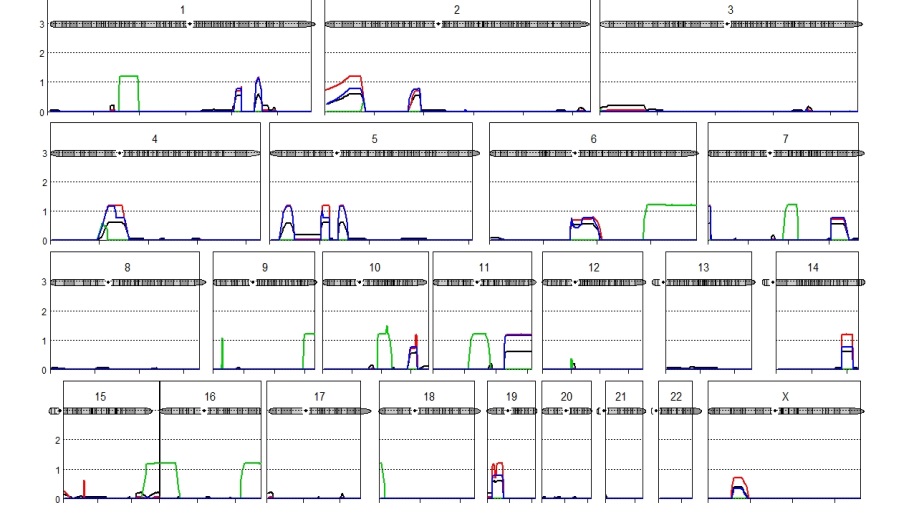
**

**
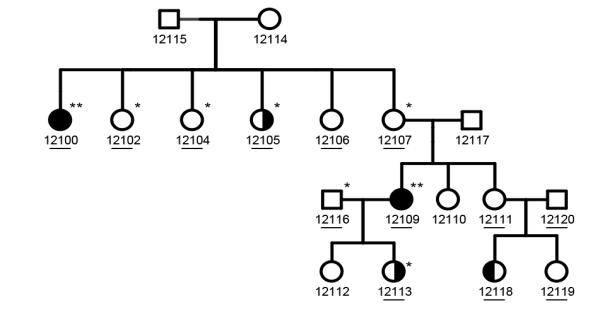
A B**

| **Chr:position** | **LOD** | **ExLOD** | **LOD_dom** | **LOD_0.6** | **Ref/Alt** | **Gene** | **AA change** | **dbSNP_ID** |
| --- | --- | --- | --- | --- | --- | --- | --- | --- |
| 5:65105886 | 0.602 | 1.194 | -1.7578 | 1.1676 | T/G | NLN | p.579I>M | rs145543140 |
| 11:117105034 | 0.602 | 1.194 | -5.1411 | 1.1676 | C/T | RNF214 | p.16P>S | rs139503238 |
| 19:6418853 | 0.552 | 0.71 | -4.5557 | 0.7725 | G/A | KHSRP | p.214R>W | / |
| 19:10088141 | 0.602 | 1.194 | -1.9278 | 0.7728 | C/T | COL5A3 | p.1045R>H | rs150379898 |

**BIPOLAR_FAMILY­_ID: PED_129**

**
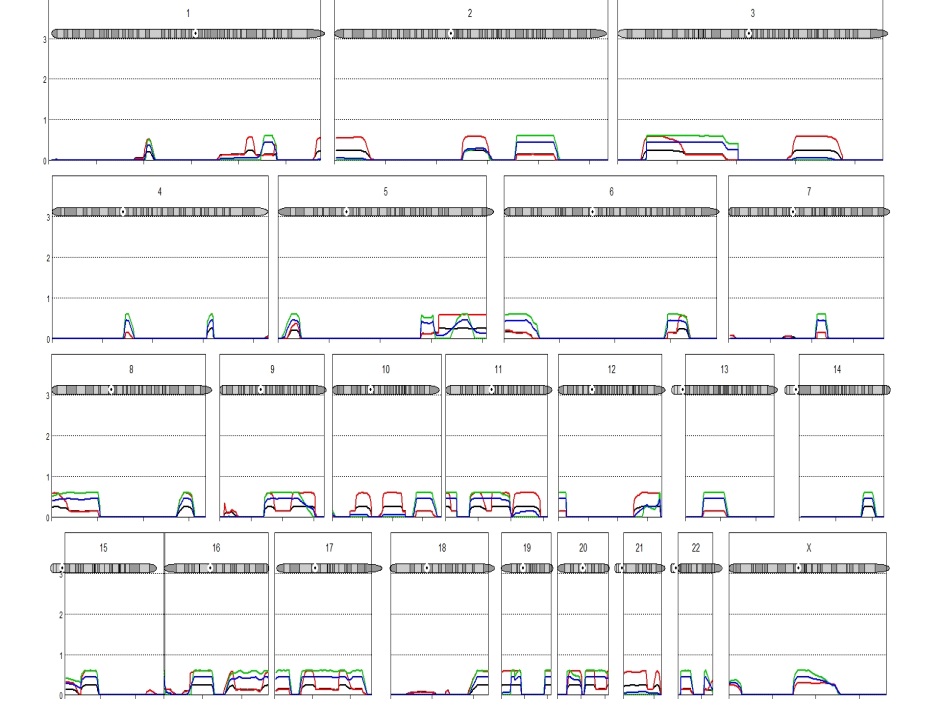
**

**
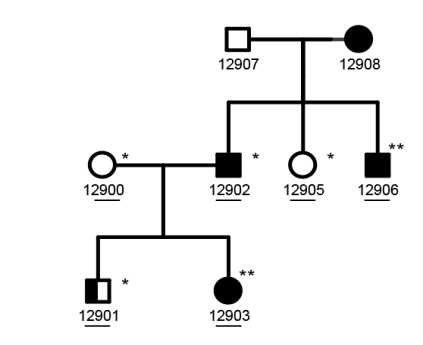
A B**

| **Chr:position** | **LOD** | **ExLOD** | **LOD_dom** | **LOD_0.6** | **Ref/Alt** | **Gene** | **AA change** | **dbSNP_ID** |
| --- | --- | --- | --- | --- | --- | --- | --- | --- |
| 3:48666132 | 0.146 | 0.138 | 0.6012 | 0.4461 | C/G | SLC26A6 | p.518G>R | rs184187143 |
|  |  |  |  |  |  |  | p.539G>R |  |
| 5:140772573 | 0.128 | 0.117 | 0.5477 | 0.4043 | C/T | PCDHGA8 | p.65R>C | rs182052960 |
| 7:99161496 | 0.146 | 0.138 | 0.6012 | 0.4461 | CCGCCT  TCCCA/C | ZNF655 | indel, fs | / |
| 9:127230383 | 0.248 | 0.591 | 0.311 | 0.2999 | C/T | GPR144 | p.690R>* | / |
| 10:70332294 | 0.248 | 0.591 | -2.9915 | 0.0556 | C/T | TET1 | p.67P>S | / |
| 10:78651443 | 0.248 | 0.591 | -2.5008 | 0.0555 | C/T | KCNMA1 | p.1007R>Q | / |
|  |  |  |  |  |  |  | p.1061R>Q |  |
|  |  |  |  |  |  |  | p.1044R>Q |  |
|  |  |  |  |  |  |  | p.1003R>Q |  |
| 11:102033237 | 0.236 | 0.561 | -0.67 | 0.0776 | C/T | YAP1 | p.208S>F | / |
|  |  |  |  |  |  |  | p.30S>F |  |
| 11:121414325 | 0.248 | 0.591 | -0.2148 | 0.1449 | G/A | SORL1 | p.585G>D | / |
| 16:21726475 | 0.248 | 0.591 | 0.6009 | 0.4469 | T/A | OTOA | splicing | / |
| 16:58011929 | -0.02 | 0.056 | -0.2524 | -0.2304 | C/A | TEPP | p.125T>N | / |
| 17:7850976 | 0.146 | 0.138 | 0.6012 | 0.4461 | A/G | CNTROB | p.694E>G | rs139292572 |
| 17:46804309 | 0.146 | 0.138 | 0.6009 | 0.4459 | C/A | HOXB13 | p.233R>L | / |
| 19:3807211 | 0.248 | 0.591 | -2.7386 | 0.0559 | T/C | ZFR2 | p.868M>V | / |
| 19:7706729 | 0.248 | 0.59 | 0.601 | 0.4472 | C/T | STXBP2 | p.187R>C | rs370053399 |
|  |  |  |  |  |  |  | p.190R>C |  |
| 19:15996828 | 0.248 | 0.592 | 0.6016 | 0.4485 | G/C | CYP4F2 | p.341L>V | rs145174239 |
| 19:58232781 | 0.248 | 0.59 | 0.6016 | 0.4485 | C/G | ZNF671 | p.225G>R | rs149668420 |
| 20:48156236 | 0.248 | 0.592 | 0.6016 | 0.4485 | C/T | PTGIS | p.182G>R | rs148768155 |

**BIPOLAR_FAMILY­_ID: PED_131**

**
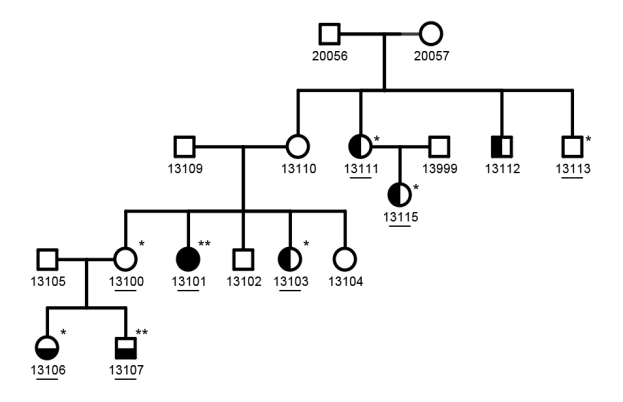

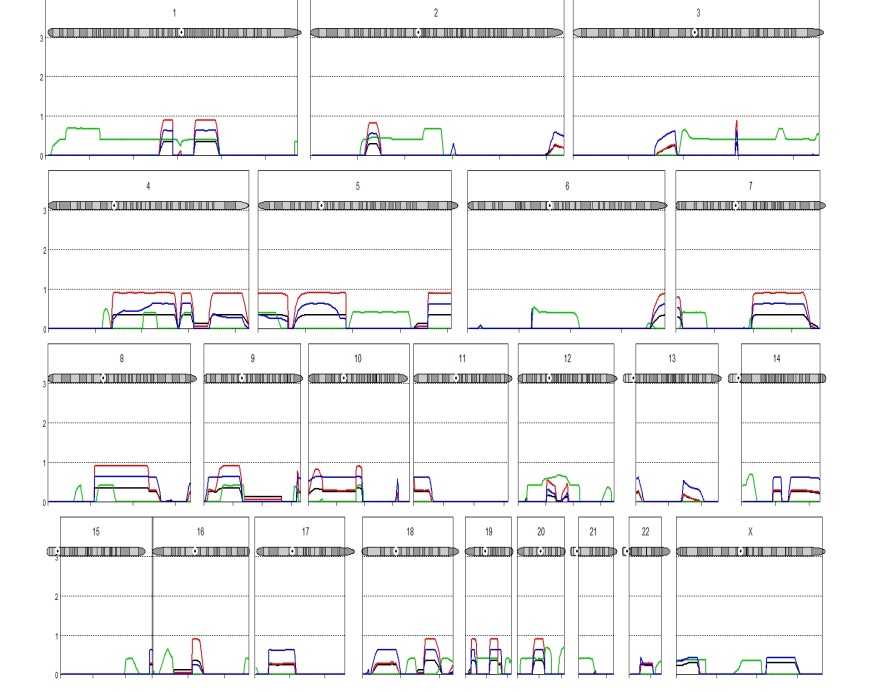
**

**A B**

| **Chr:position** | **LOD** | **ExLOD** | **LOD_dom** | **LOD_0.6** | **Ref/Alt** | **Gene** | **AA change** | **dbSNP_ID** |
| --- | --- | --- | --- | --- | --- | --- | --- | --- |
| 5:10227711 | 0.345 | 0.9 | 0.216 | 0.2551 | G/A | FAM173B | p.182R>* | rs200658616 |
| 5:41909884 | 0.347 | 0.902 | -0.6341 | 0.6257 | C/A | C5orf51 | p.82L>I | rs142045369 |
| 5:176861035 | 0.345 | 0.9 | -0.6107 | 0.6267 | C/T | GRK6 | p.287R>W | / |
| 7:92161828 | 0.345 | 0.899 | -0.6159 | 0.6274 | G/A | RBM48 | p.138R>Q | rs372855385 |
| 8:41550195 | 0.346 | 0.901 | 0.4061 | 0.6342 | C/T | ANK1 | p.1277V>M | rs148942046 |
|  |  |  |  |  |  |  | p.1318V>M |  |
| 9:37746714 | 0.313 | 0.844 | 0.4096 | 0.5807 | C/T | FRMPD1 | p.1562T>M | rs138292555 |
| 10:67829108 | 0.346 | 0.901 | 0.406 | 0.6347 | T/C | CTNNA3 | p.706K>R | / |
| 11:1267115 | 0.297 | 0.585 | -0.8046 | 0.6233 | C/A | MUC5B | p.3002T>K | rs200554635 |
| 11:2909582 | 0.249 | 0.279 | -2.504 | 0.6218 | G/T | SLC22A18AS | p.197A>E | rs187086795 |
| 11:5699642 | 0.248 | 0.278 | -2.5154 | 0.6213 | G/C | TRIM5 | p.179T>S | / |
| 12:53509433 | 0.109 | 0.375 | 0.6189 | 0.1767 | G/C | SOAT2 | p.235E>Q | rs138678118 |
| 14:55864124 | 0.249 | 0.279 | -2.4589 | 0.62 | G/A | ATG14 | p.84R>* | / |
| 14:105959012 | 0.197 | 0.223 | -1.3044 | 0.5576 | C/T | C14orf80 | p.176R>W | / |
|  |  |  |  |  |  |  | p.135R>W |  |
|  |  |  |  |  |  |  | p.137R>W |  |
| 19:37677213 | 0.347 | 0.903 | 0.4055 | 0.6353 | G/C | ZNF585B | p.409S>W | rs368332359 |
| 20:25277094 | 0.346 | 0.902 | 0.4067 | 0.6346 | G/C | PYGB | p.823R>P | / |
| 20:31606585 | 0.346 | 0.902 | 0.4067 | 0.6347 | T/C | BPIFB2 | p.271L>P | rs367964762 |
| 20:35695185 | 0.346 | 0.902 | 0.396 | 0.6345 | G/C | RBL1 | p.263A>G | rs66494730 |

**BIPOLAR_FAMILY­_ID: PED_138**

**
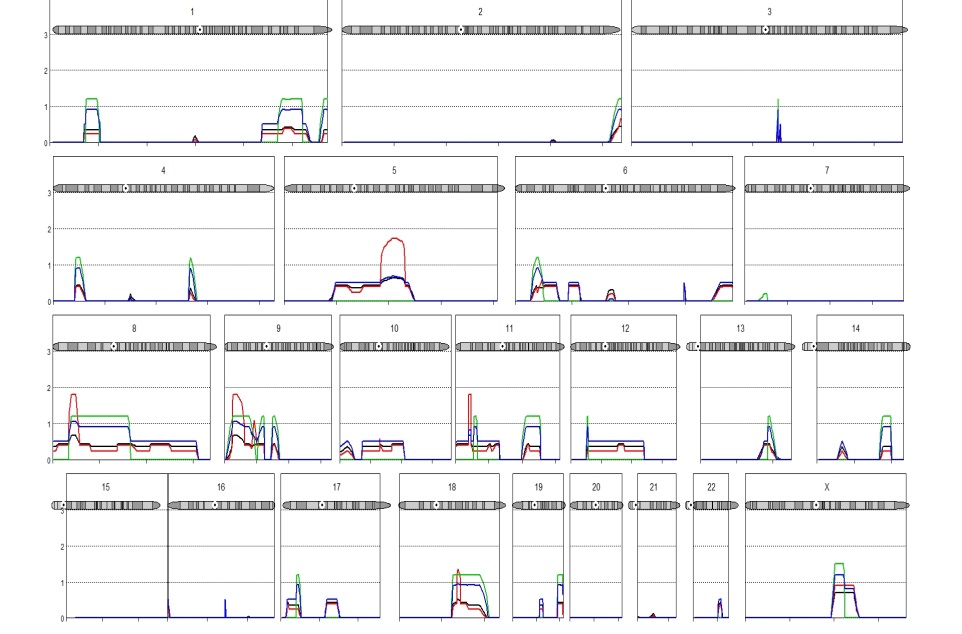
**

**
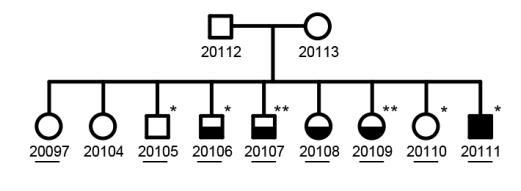
A B**

| **Chr:position** | **LOD** | **ExLOD** | **LOD_dom** | **LOD_0.6** | **Ref/Alt** | **Gene** | **AA change** | **dbSNP_ID** |
| --- | --- | --- | --- | --- | --- | --- | --- | --- |
| 6:12124136 | 0.432 | 0.396 | -1.7997 | 0.5137 | C/G | HIVEP1 | p.1370Q>E | rs751637189 |
| 6:31122368 | 0.43 | 0.394 | -2.0991 | 0.5112 | G/A | CCHCR1 | p.200R>C | rs375819943 |
|  |  |  |  |  |  |  | p.236R>C |  |
|  |  |  |  |  |  |  | p.147R>C |  |
| 6:31601179 | 0.43 | 0.393 | -1.941 | 0.5106 | C/T | PRRC2A | p.1448P>L | rs151200532 |
| 6:31922334 | 0.43 | 0.393 | -1.941 | 0.5106 | C/T | RDBP | p.247R>H | rs373302915 |
| 9:33466115 | 0.431 | 0.395 | 1.1597 | 0.8869 | T/G | NOL6 | p.773H>P | / |
| 9:79320991 | 0.432 | 0.396 | 1.2 | 0.9112 | C/T | PRUNE2 | p.2067A>T | rs114221706 |
| 10:33196047 | 0.356 | 0.24 | -2.6546 | 0.5133 | G/C | ITGB1 | p.786P>A | rs112423286 |
| 10:55566719 | 0.356 | 0.24 | -2.0473 | 0.5128 | G/A | PCDH15 | p.1557R>C | rs570828018 |
|  |  |  |  |  |  |  | p.1552R>C |  |
| 12:54902264 | 0.356 | 0.24 | -3.613 | 0.5135 | G/A | NCKAP1L | p.102R>Q | rs149360088 |
|  |  |  |  |  |  |  | p.152R>Q |  |
| 17:3195207 | 0.418 | 0.368 | -4.9073 | 0.5138 | C/T | OR3A1 | p.224V>M | rs142082644 |
| 17:48774378 | 0.431 | 0.396 | -7.764 | 0.513 | C/T | ANKRD40 | p.295V>M | rs144124178 |
| 19:39001390 | 0.356 | 0.24 | -8.3509 | 0.5135 | G/A | RYR1 | p.3031A>T | rs756311466 |
| 19:55685027 | 0.432 | 0.396 | 1.2003 | 0.9114 | A/C | SYT5 | p.329L>R | rs754474231 |
| X:107976904 | 0.705 | 0.903 | 1.5027 | 1.2001 | G/A | IRS4 | p.891R>* | / |

**BIPOLAR_FAMILY­_ID: PED_198**

**
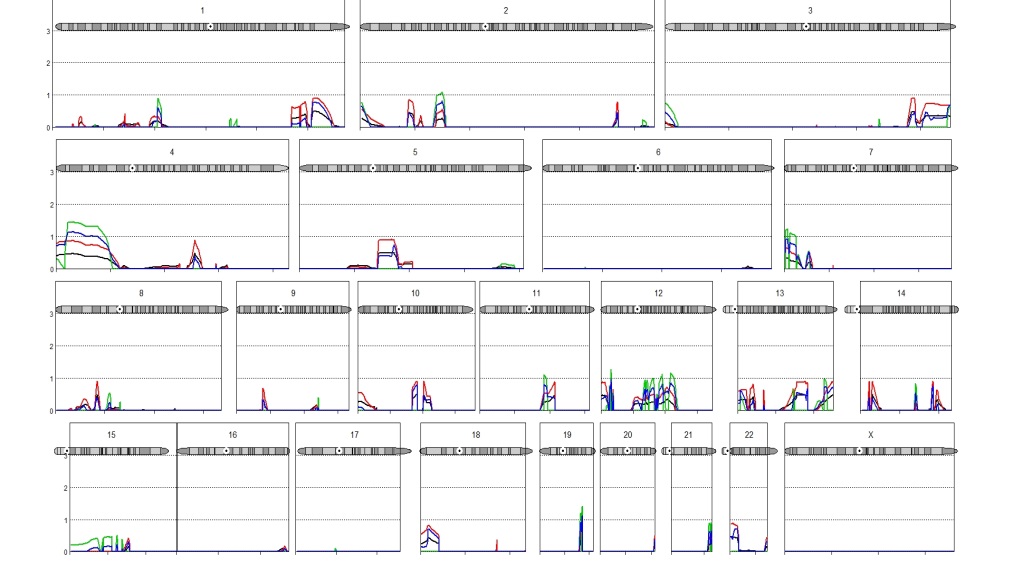
**

**A B
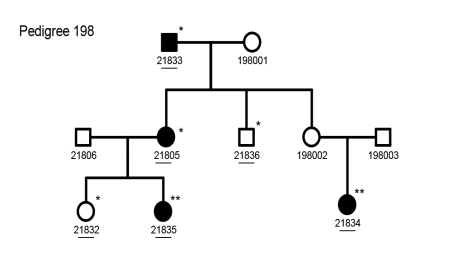
**

| **Chr:position** | **LOD** | **ExLOD** | **LOD_dom** | **LOD_0.6** | **Ref/Alt** | **Gene** | **AA change** | **dbSNP_ID** |
| --- | --- | --- | --- | --- | --- | --- | --- | --- |
| 1:1216667 | 0.352 | 0.706 | -1.7269 | 0.5906 | G/A | SCNN1D | p.23G>S | rs751882083 |
| 1:248813732 | 0.479 | 0.888 | 1.4651 | 1.1584 | C/A | OR2T27 | p.152G>W | / |
| 17:39884458 | 0.49 | 0.902 | -0.1296 | 0.7947 | G/A | HAP1 | p.399R>W | rs573288812 |
|  |  |  |  |  |  |  | p.407R>W |  |
| 19:56369683 | 0.485 | 0.895 | 1.4737 | 1.1665 | AGT/A | NLRP4 | indel, fs | rs773160721 |

1. **CNV STUDIES**

Two patients per family (30 patients total), were analysed for copy number variants (CNVs) using the CytoScan® HD Array, which includes 2.6 million copy number and 750,000 SNP probes, in a dense array to detect small structural variants. Data were analysed with the Affymetrix Chromosome Analysis Suite (ChAS) software (ThermoFisher, Waltham, MA, USA) considering at least 25 altered probes spanning a minimum of 25 kb for either deletions or duplications. The first analysis generated 977 putative CNVs in all 30 individuals. Subsequent filtering was performed using the following criteria: 1) exclusion of CNVs with a frequency >0.05 in general population in order to exclude polymorphic structural variants;^13^ 2) exclusion of all intergenic CNVs more than 50 kb away from any coding gene; 3) exclusion of deletions that were not consistent with WES-derived loss of heterozygosity pattern and ChAS calls from CytoScan data. A total of 27 CNVs were included for experimental validation via a SYBR Green-based quantitative PCR (qPCR) method, using two independent amplicon probes for each candidate deletion/duplication, each compared with two different reference amplicon probes in the *FOXP2* and *RNF20* genes. Each individual experiment was performed in quadruplicate. Experimental details are available upon request.

For validation purposes we also examined CNV calls from Illumina 660quad SNP array data from one affected individual in each family, which were estimated using the PennCNV algorithm,^14^ as well as CNVs inferred from read depth information from the WES data using ESCAVATOR.^15^ Illumina Human 660quad chip data was available for 15 subjects (PED_1-63, PED_2-207, PED_5-518, PED_11-1100, PED_17-1720, PED_18-1808, PED_19-1917, PED_74-7400, PED_84-8402, PED_113-11300, PED_121-12109, PED_129-12902, PED_131-13101, PED_138-20111, PED_198-21805), but CNV calls were made across a cohort of 400 unrelated bipolar patients. All CNVs detected by CytoScan and validated by an alternative method, were finally assessed for consistency with underlying haplotype structure in each family.

1. **STATISTICAL AND METHOD PROCEDURES**

**Spearman’s correlation**

A non-parametric correlation analysis was used to assess the relationship between age of onset (AOO) and genome-wide burden of truncating variants. The age of onset was considered as the age of first experience of a major mood episode, either depression or mania. We included in our study, all subjects with AOO ranging from 15-52 years (excluding n=5 with AOO≤14, and n=3 with AOO≥55), a range fitting the first standard deviation of three overlapping Gaussian distributions of AOO observed in European patients.^16-18^ Our analysis included 58 individuals affected with BD-I (n=36), SZMA (n=12), BD-II (n=4) or RUD (n=6). We considered likely gene-disruptive variants as nonsense mutations, indels leading to frameshifts, splice variants and start-lost variants, regardless of sharing in other family members. All variants were checked for consistency with haplotypes in each family, read coverage and their frequency in ExAC database (exac.broadinstitute.org). Brain expressed genes were those identified from the BrainSpan developmental transcriptome RNA-seq dataset, with a Reads Per Kilobase per Million mapped reads (RPKM) value of greater than 1, considering all brain regions at any developmental age (brainspan.org).

**Enrichment analyses**

To test enrichment of potential etiologic variants in functional categories, we calculated an empirical P-value for each functional category using a null distribution of overlap counts seen in randomly drawn sets of genes. Each set of randomly drawn genes was of equal size to the genes containing potential etiologic variants, and matched on a per-gene basis on two potentially confounding metrics: *i)* the length of coding sequence for each gene, measured in bp of callable coding sequence; and *ii)* a functional constraint score for each gene. The callable coding sequence was defined as the number of bp covered to at least 10×, and the median value across all samples was used as the matching value for each gene. The functional constraint score used was the 'non-psych' (individuals with psychiatric diagnoses removed) missense Z-score for each gene, obtained from the ExAC database. The empirical P-value was calculated by comparing the overlap seen in potential etiologic variants to the overlaps seen in 10,000 randomly drawn matching gene sets described.

1. ***DE NOVO* VARIANT STUDY**

For the *de novo* variant (DN) study we selected all nuclear families from within the 15 extended families for whom we had WES for both parents and their offspring, regardless of diagnosis. The total number of individuals available for this study included 32 individuals, 22 of which were affected offspring and 10 unaffected offspring, and are represented in Supplemental Figure S7.The bioinformatic analysis included the selection of all variants with potential Mendelian error, resulting in a list of 18,495 variants. A threshold in the read depth of the alternative allele (AD>20) was used to reduce the number of false positives, yielding 2,649 putative *de novo* variants. These underwent additional QC process, via manual checking of reads at each putative variant in IGV v2.3.34 in all family members, resulting in exclusion of 2,587 false positive variants and the identification of 62 putative *de novo* variants for validation by Sanger sequencing. A total of 45 *de novo* variants were validated (73%) by targeted Sanger sequencing, which included 31 coding variants and 14 intronic variants of unknown functional impact. Normality of DN distribution was tested for BD patients (*P*=0.006) and unaffected relatives (*P*=0.016), then a non-parametric test (Mann-Whitney U) was performed to assess any difference between BD cases and unaffected. The rate of coding DN was slightly higher in unaffected (1.10=11/10) compared to the affected individuals (0.909=20/22), but this was not significantly different (*P*=0.889). This difference remains non-significant when excluding silent DN variants (n=13 vs 7 missense, splicing or truncating variants; Mann-Whitney-U *P*=0.62). We note that a single unaffected subject (PED_11-1115) had an unusually large number of DN, with 5 coding and events detected (Table S3). There were eight subjects (6 BD patients and 2 unaffected relatives) for whom we did not detect any *de novo* variant (coding or intronic). When possible we derived the parental chromosome on which the DN originated, where another polymorphism was present in the same reads to enable parent of origin to be determined (Table S3).

1. **SUPPLEMENTARY REFERENCES**

1. Adams LJ, Mitchell PB, Fielder SL, Rosso A, Donald JA, Schofield PR. A susceptibility locus for bipolar affective disorder on chromosome 4q35. *Am J Hum Genet* 1998; **62**(5)**:** 1084-1091.

2. Badenhop RF, Moses MJ, Scimone A, Adams LJ, Kwok JB, Jones AM*, et al*. Genetic refinement and physical mapping of a 2.3 Mb probable disease region associated with a bipolar affective disorder susceptibility locus on chromosome 4q35. *Am J Med Genet B Neuropsychiatr Genet* 2003; **117B**(1)**:** 23-32.

3. Fullerton JM, Donald JA, Mitchell PB, Schofield PR. Two-dimensional genome scan identifies multiple genetic interactions in bipolar affective disorder. *Biol Psychiatry* 2010; **67**(5)**:** 478-486.

4. Fullerton JM, Liu Z, Badenhop RF, Scimone A, Blair IP, Van Herten M*, et al*. Genome screen of 15 Australian bipolar affective disorder pedigrees supports previously identified loci for bipolar susceptibility genes. *Psychiatr Genet* 2008; **18**(4)**:** 156-161.

5. McAuley EZ, Blair IP, Liu Z, Fullerton JM, Scimone A, Van Herten M*, et al*. A genome screen of 35 bipolar affective disorder pedigrees provides significant evidence for a susceptibility locus on chromosome 15q25-26. *Mol Psychiatry* 2009; **14**(5)**:** 492-500.

6. National Institute of Mental Health. *NIMH Genetics Initiative: Family Interview for Genetic Studies (FIGS)*: Rockville, MD, 1992.

7. Nurnberger JI, Jr., Blehar MC, Kaufmann CA, York-Cooler C, Simpson SG, Harkavy-Friedman J*, et al*. Diagnostic interview for genetic studies. Rationale, unique features, and training. NIMH Genetics Initiative. *Arch Gen Psychiatry* 1994; **51**(11)**:** 849-859; discussion 863-844.

8. American Psychiatric Association. *Diagnostic and Statistical Manual of Mental Disorders Fourth Edn (DSM-IV)* Washington, DC, 1994.

9. Purcell S, Neale B, Todd-Brown K, Thomas L, Ferreira MA, Bender D*, et al*. PLINK: a tool set for whole-genome association and population-based linkage analyses. *Am J Hum Genet* 2007; **81**(3)**:** 559-575.

10. Cichon S, Muhleisen TW, Degenhardt FA, Mattheisen M, Miro X, Strohmaier J*, et al*. Genome-wide association study identifies genetic variation in neurocan as a susceptibility factor for bipolar disorder. *Am J Hum Genet* 2011; **88**(3)**:** 372-381.

11. Muhleisen TW, Leber M, Schulze TG, Strohmaier J, Degenhardt F, Treutlein J*, et al*. Genome-wide association study reveals two new risk loci for bipolar disorder. *Nat Commun* 2014; **5:** 3339.

12. Psychiatric Genomics Consortium Bipolar Disorder Working Group. Large-scale genome-wide association analysis of bipolar disorder identifies a new susceptibility locus near ODZ4. *Nat Genet* 2011; **43**(10)**:** 977-983.

13. Conrad DF, Pinto D, Redon R, Feuk L, Gokcumen O, Zhang Y*, et al*. Origins and functional impact of copy number variation in the human genome. *Nature* 2010; **464**(7289)**:** 704-712.

14. Wang K, Li M, Hadley D, Liu R, Glessner J, Grant SF*, et al*. PennCNV: an integrated hidden Markov model designed for high-resolution copy number variation detection in whole-genome SNP genotyping data. *Genome research* 2007; **17**(11)**:** 1665-1674.

15. Magi A, Tattini L, Cifola I, D'Aurizio R, Benelli M, Mangano E*, et al*. EXCAVATOR: detecting copy number variants from whole-exome sequencing data. *Genome biology* 2013; **14**(10)**:** R120.

16. Bellivier F, Etain B, Malafosse A, Henry C, Kahn JP, Elgrabli-Wajsbrot O*, et al*. Age at onset in bipolar I affective disorder in the USA and Europe. *World J Biol Psychiatry* 2014; **15**(5)**:** 369-376.

17. Azorin JM, Bellivier F, Kaladjian A, Adida M, Belzeaux R, Fakra E*, et al*. Characteristics and profiles of bipolar I patients according to age-at-onset: findings from an admixture analysis. *J Affect Disord* 2013; **150**(3)**:** 993-1000.

18. Nowrouzi B, McIntyre RS, MacQueen G, Kennedy SH, Kennedy JL, Ravindran A*, et al*. Admixture analysis of age at onset in first episode bipolar disorder. *J Affect Disord* 2016; **201:** 88-94.

1. **SUPPLEMENTARY TABLES**

**Supplementary Table S1. Rare variants identified after quality control and validation.** The list consists of 532 variants shared in at least 3 affected (and maximum one unaffected sibling) called potentially-etiologic-variants (PEV) and 541 shared in 1 to 3 unaffected (and maximum one affected) likely-neutral-variants (LNV). Abbreviations: mis, missense; fs, frameshift; spl, splice variant; start, start-lost variant.

| **PED** | **Chr:position** | **Category** | **Ref/Alt** | **N cases** | **N unaffected** | **Gene** | **AA change** | **CAROL score** | **Annotation** | **EVSEURMAF** | **dbSNP 147** |
| --- | --- | --- | --- | --- | --- | --- | --- | --- | --- | --- | --- |
| 1 | 1:151665435 | PEV | G/C | 4 | 1 | SNX27 | p.480D>H | 1.000 | mis | 0.000349 | rs145045028 |
| 1 | 2:10930853 | PEV | G/C | 4 | 1 | PDIA6 | p.231R>G | 0.999 | mis | . | . |
| 1 | 2:152342389 | PEV | T/C | 3 | 1 | NEB | p.8488K>R | 0.999 | mis | 0.000714 | rs201714437 |
| 1 | 2:153463871 | PEV | C/T | 3 | 1 | FMNL2 | p.299R>C | 1.000 | mis | 0.000244 | rs201721916 |
| 1 | 2:167289012 | PEV | T/A | 4 | 1 | SCN7A | p.803D>V | 0.990 | mis | 0.003927 | rs148715564 |
| 1 | 2:190426750 | PEV | C/T | 5 | 1 | SLC40A1 | p.524V>I | 0.999 | mis | 0.000233 | rs142456282 |
| 1 | 2:196728886 | PEV | A/G | 5 | 1 | DNAH7 | p.2498I>T | 1.000 | mis | 0.004366 | rs200303203 |
| 1 | 3:74383929 | PEV | G/T | 4 | 1 | CNTN3 | p.542A>E | 0.999 | mis | . | . |
| 1 | 3:124281792 | PEV | C/T | 5 | 1 | KALRN | p.1678R>W | 1.000 | mis | 0.000119 | rs368179672 |
| 1 | 4:41615111 | PEV | T/TG | 4 | 0 | LIMCH1 | . | . | fs | . | rs751130840 |
| 1 | 5:140563965 | PEV | G/A | 4 | 1 | PCDHB16 | p.611E>K | 1.000 | mis | 0.000133 | rs200413005 |
| 1 | 5:141233971 | PEV | G/A | 4 | 1 | PCDH1 | p.1117T>I | 1.000 | mis | 0.001984 | rs75207818 |
| 1 | 5:172513516 | PEV | G/A | 4 | 1 | CREBRF | p.8G>R | 1.000 | mis | . | . |
| 1 | 7:24849498 | PEV | G/A | 3 | 1 | OSBPL3 | p.749R>W | 0.999 | mis | 0.000233 | rs371721619 |
| 1 | 7:73183979 | PEV | G/A | 4 | 1 | CLDN3 | p.134P>L | 1.000 | mis | 0.006744 | rs139191328 |
| 1 | 8:105502966 | PEV | C/T | 6 | 1 | LRP12 | p.820D>N | 0.997 | mis | 0.004651 | rs150116835 |
| 1 | 8:113649070 | PEV | T/C | 6 | 1 | CSMD3 | p.1127S>G | 1.000 | mis | . | rs765216700 |
| 1 | 10:46968674 | PEV | AATCTC/A | 3 | 1 | SYT15 | . | . | fs | 0.007254 | rs557452410 |
| 1 | 11:93400950 | PEV | G/A | 3 | 1 | KIAA1731 | p.96G>R | 1.000 | mis | . | . |
| 1 | 12:50471039 | PEV | G/A | 4 | 1 | ASIC1 | p.201R>Q | 0.999 | mis | . | rs775952643 |
| 1 | 12:54405034 | PEV | C/T | 3 | 0 | HOXC8 | p.200R>* | . | stop | . | . |
| 1 | 12:113612847 | PEV | G/A | 4 | 1 | DDX54 | p.290R>W | 1.000 | mis | . | rs201173369 |
| 1 | 12:122676056 | PEV | A/G | 3 | 0 | LRRC43 | p.344Y>C | 0.998 | mis | 0.003404 | rs185427201 |
| 1 | 14:20711179 | PEV | G/C | 3 | 0 | OR11H4 | p.77A>P | 0.999 | mis | 0.002791 | rs147781090 |
| 1 | 14:32562222 | PEV | T/A | 3 | 1 | ARHGAP5 | p.783C>S | 0.999 | mis | 0.000465 | rs200248752 |
| 1 | 14:75330435 | PEV | C/T | 5 | 0 | PROX2 | p.35D>N | 0.999 | mis | 0.002043 | rs180960227 |
| 1 | 16:597925 | PEV | T/C | 4 | 1 | SOLH | p.363C>R | 1.000 | mis | 0.00107 | rs144052786 |
| 1 | 16:3255148 | PEV | T/C | 3 | 0 | OR1F1 | p.301L>P | 1.000 | mis | . | . |
| 1 | 16:4390400 | PEV | G/A | 3 | 0 | PAM16 | p.1023R>C | 1.000 | mis | . | rs558716310 |
| 1 | 16:28617472 | PEV | G/A | 3 | 0 | SULT1A1 | p.227T>M | 1.000 | mis | 0.004073 | rs150459557 |
| 1 | 16:70954909 | PEV | A/G | 3 | 0 | HYDIN | p.2456F>S | 0.999 | mis | 0.001114 | rs200385024 |
| 1 | 17:18022538 | PEV | C/G | 3 | 1 | MYO15A | p.142L>V | 0.991 | mis | . | . |
| 1 | 17:26731862 | PEV | G/A | 3 | 1 | SLC46A1 | p.285Q>* | . | stop | . | . |
| 1 | 17:27958088 | PEV | T/C | 3 | 1 | SSH2 | p.1348K>R | 0.995 | mis | 0.000116 | rs374938013 |
| 1 | 17:41243948 | PEV | C/A | 3 | 0 | BRCA1 | p.1200Q>H | 0.998 | mis | 0 | rs56214134 |
| 1 | 17:48247606 | PEV | C/G | 5 | 1 | SGCA | p.284R>G | 1.000 | mis | 0.000116 | rs137852623 |
| 1 | 19:6707129 | PEV | G/A | 3 | 1 | C3 | p.735R>W | 1.000 | mis | 0.003732 | rs117793540 |
| 1 | 19:7585606 | PEV | C/A | 3 | 1 | ZNF358 | p.493S>Y | 0.999 | mis | 0.000581 | rs147948386 |
| 1 | 19:33409218 | PEV | CA/C | 3 | 1 | CEP89 | . | . | fs | 0.000121 | rs748645203 |
| 1 | 19:54666442 | PEV | ACAGT/A | 4 | 0 | TMC4 | . | . | fs | 0.000485 | rs758351399 |
| 1 | 19:55593646 | PEV | C/G | 4 | 0 | EPS8L1 | . | 1.000 | mis | 0.000467 | rs142793216 |
| 1 | 22:46930013 | PEV | C/A | 3 | 1 | CELSR1 | p.1019A>S | 1.000 | mis | 0.002558 | rs61737811 |
| 2 | 1:3418428 | PEV | G/A | 3 | 1 | MEGF6 | p.749A>V | 0.998 | mis | 0.000969 | rs200472001 |
| 2 | 1:20021023 | PEV | GAA/G | 5 | 1 | TMCO4 | . | . | fs | 0.002302 | rs376795192 |
| 2 | 1:53542882 | PEV | A/G | 3 | 0 | PODN | p.230N>S | 1.000 | mis | . | . |
| 2 | 1:89299123 | PEV | T/C | 3 | 1 | PKN2 | p.983W>R | 1.000 | mis | 0.00036 | rs184754507 |
| 2 | 1:98205983 | PEV | C/G | 4 | 1 | DPYD | p.96D>H | 1.000 | mis | . | rs773159364 |
| 2 | 1:152286458 | PEV | CCCTGT/C | 3 | 1 | FLG | . | . | fs | . | . |
| 2 | 2:31572670 | PEV | C/T | 3 | 0 | XDH | p.951G>R | 0.999 | mis | 0.000233 | rs142675390 |
| 2 | 2:179431633 | PEV | C/T | 5 | 1 | TTN | p.24768R>H | 1.000 | mis | 0.001585 | rs72648206 |
| 2 | 2:218712290 | PEV | G/A | 6 | 0 | TNS1 | p.859R>W | 0.996 | mis | 0.007442 | rs61746994 |
| 2 | 2:226378176 | PEV | A/T | 3 | 0 | NYAP2 | p.104D>V | 1.000 | mis | . | rs755957714 |
| 2 | 3:36527665 | PEV | G/A | 4 | 1 | STAC | p.204R>H | 0.992 | mis | . | rs754667535 |
| 2 | 3:134670233 | PEV | C/G | 3 | 1 | EPHB1 | p.48Y>* | . | stop | 0.000117 | rs371726436 |
| 2 | 4:4207798 | PEV | C/A | 4 | 0 | OTOP1 | . | . | spl | 0.000698 | rs143383113 |
| 2 | 4:36163089 | PEV | C/G | 3 | 1 | ARAP2 | . | 0.999 | mis | 0.003372 | rs35400920 |
| 2 | 4:74477479 | PEV | TC/T | 5 | 1 | RASSF6 | . | . | fs | . | . |
| 2 | 5:89990447 | PEV | G/A | 3 | 1 | GPR98 | p.2625R>H | 0.999 | mis | 0.003115 | rs201214794 |
| 2 | 5:140076960 | PEV | G/A | 3 | 1 | HARS2 | p.389R>Q | 1.000 | mis | . | . |
| 2 | 5:150943061 | PEV | G/T | 3 | 1 | FAT2 | p.1133N>K | 1.000 | mis | 0.003605 | rs138913417 |
| 2 | 6:26285612 | PEV | G/A | 4 | 0 | HIST1H4H | p.39A>V | 0.990 | mis | . | . |
| 2 | 6:72960085 | PEV | A/G | 3 | 0 | RIMS1 | p.239Q>R | 0.993 | mis | 0.001097 | rs199629596 |
| 2 | 7:82579610 | PEV | C/T | 5 | 1 | PCLO | p.3432D>N | 0.999 | mis | 0.000486 | rs202185916 |
| 2 | 7:100371479 | PEV | G/C | 5 | 1 | ZAN | p.1924G>R | 0.993 | mis | . | rs71555302 |
| 2 | 7:113558573 | PEV | C/T | 4 | 1 | PPP1R3A | p.160R>K | 1.000 | mis | . | . |
| 2 | 7:138863037 | PEV | A/C | 4 | 1 | TTC26 | p.383N>H | 0.999 | mis | . | rs769849962 |
| 2 | 8:19250891 | PEV | C/A | 3 | 0 | SH2D4A | p.371L>I | 1.000 | mis | 0.000465 | rs187247814 |
| 2 | 9:33037072 | PEV | C/T | 4 | 1 | DNAJA1 | p.312R>C | 0.999 | mis | . | . |
| 2 | 9:79318983 | PEV | TC/AA | 3 | 0 | PRUNE2 | p.2516E>L | . | mis | 0.000977 | . |
| 2 | 9:89771533 | PEV | C/T | 3 | 1 | C9orf170 | p.72Q>* | . | stop | . | . |
| 2 | 9:135218103 | PEV | A/C | 4 | 0 | SETX | p.158L>V | 1.000 | mis | 0.005465 | rs145438764 |
| 2 | 10:103908614 | PEV | G/A | 4 | 1 | PPRC1 | p.1553R>H | 0.996 | mis | . | rs763766248 |
| 2 | 10:135440226 | PEV | G/C | 3 | 0 | FRG2B | p.7D>E | 0.996 | mis | 0.003488 | rs201470110 |
| 2 | 11:6622602 | PEV | CAT/C | 3 | 1 | RRP8 | . | . | fs | 0.00109 | rs778595582 |
| 2 | 11:17553067 | PEV | C/T | 5 | 1 | USH1C | p.43V>M | 1.000 | mis | 0.000116 | rs145500807 |
| 2 | 11:27389562 | PEV | G/A | 3 | 0 | LGR4 | p.903S>L | 1.000 | mis | . | rs759405820 |
| 2 | 11:55944467 | PEV | C/T | 4 | 1 | OR5J2 | p.125A>V | 0.999 | mis | 0.00966 | rs149623931 |
| 2 | 11:64521350 | PEV | C/T | 4 | 1 | PYGM | . | . | spl | . | rs759657964 |
| 2 | 11:67265636 | PEV | G/A | 4 | 1 | PITPNM1 | p.548R>C | 0.999 | mis | 0.005007 | rs143726971 |
| 2 | 12:2973617 | PEV | C/G | 3 | 1 | FOXM1 | p.364V>L | 0.999 | mis | 0.000233 | rs141320223 |
| 2 | 12:7521535 | PEV | A/G | 3 | 1 | CD163L1 | p.1356S>P | 0.999 | mis | 0.002907 | rs145256685 |
| 2 | 12:8610545 | PEV | T/A | 3 | 1 | CLEC6A | p.28I>N | 0.999 | mis | 0.00093 | rs75947861 |
| 2 | 12:11461804 | PEV | C/T | 4 | 1 | PRB4 | p.38G>E | 0.999 | mis | 0.001165 | rs150367358 |
| 2 | 12:110457073 | PEV | G/A | 4 | 1 | ANKRD13A | p.225R>Q | 0.999 | mis | . | rs745337377 |
| 2 | 12:112681251 | PEV | C/T | 4 | 1 | HECTD4 | p.1488V>M | 1.000 | mis | 0.003875 | rs61941332 |
| 2 | 15:42149488 | PEV | C/T | 4 | 1 | SPTBN5 | p.2822G>S | 0.999 | mis | 0.00083 | rs182480347 |
| 2 | 15:51829044 | PEV | G/A | 4 | 1 | DMXL2 | p.545R>W | 1.000 | mis | . | rs558303192 |
| 2 | 16:84353055 | PEV | C/T | 3 | 1 | WFDC1 | p.147T>M | 1.000 | mis | 0.000233 | rs141305670 |
| 2 | 17:37825998 | PEV | C/T | 4 | 1 | PNMT | p.107R>C | 1.000 | mis | 0.005233 | rs72554035 |
| 2 | 17:40948188 | PEV | T/C | 4 | 1 | WNK4 | p.1160I>T | 1.000 | mis | . | rs747683477 |
| 2 | 19:1057343 | PEV | G/A | 4 | 1 | ABCA7 | p.1599V>M | 1.000 | mis | 0.003605 | rs117187003 |
| 2 | 19:2917589 | PEV | C/T | 4 | 1 | ZNF57 | p.324R>* | . | stop | . | . |
| 2 | 19:4491797 | PEV | C/T | 4 | 1 | HDGFRP2 | p.215P>S | 0.995 | mis | 0.001095 | rs201430193 |
| 2 | 19:8592358 | PEV | G/C | 3 | 0 | MYO1F | p.780R>G | 0.994 | mis | 0.00036 | rs201198914 |
| 2 | 20:2777921 | PEV | C/T | 3 | 1 | CPXM1 | p.250R>H | 1.000 | mis | 0.001283 | rs41301840 |
| 2 | 20:25457361 | PEV | G/A | 3 | 1 | NINL | p.856R>W | 0.999 | mis | . | rs751019836 |
| 2 | 20:34785938 | PEV | A/G | 3 | 1 | EPB41L1 | p.548K>R | 0.999 | mis | 0.002558 | rs144426436 |
| 2 | 20:52775622 | PEV | C/T | 5 | 1 | CYP24A1 | p.344R>H | 0.994 | mis | 0 | rs116548533 |
| 2 | 22:18905934 | PEV | A/G | 4 | 1 | PRODH | p.333L>P | 1.000 | mis | 0.006744 | rs2904551 |
| 2 | 22:41742139 | PEV | G/A | 4 | 1 | ZC3H7B | p.531G>D | 0.999 | mis | 0.002907 | rs113935023 |
| 2 | X:57475022 | PEV | G/T | 3 | 1 | FAAH2 | p.432E>* | . | stop | 0.002675 | rs144419744 |
| 2 | X:153030991 | PEV | A/G | 4 | 0 | PLXNB3 | . | . | start | 0.000447 | rs148960424 |
| 5 | 1:3700673 | PEV | A/C | 3 | 1 | LRRC47 | . | 0.997 | mis | . | rs750920435 |
| 5 | 1:15832495 | PEV | T/G | 3 | 1 | CASP9 | p.237H>P | 1.000 | mis | 0.002209 | rs146054764 |
| 5 | 1:24663277 | PEV | G/A | 3 | 1 | GRHL3 | p.145R>H | 0.992 | mis | 0.000116 | rs150060454 |
| 5 | 1:27690008 | PEV | G/A | 3 | 1 | MAP3K6 | p.299T>M | 1.000 | mis | . | rs747018492 |
| 5 | 1:112305627 | PEV | C/T | 3 | 1 | DDX20 | . | 1.000 | mis | 0.000465 | rs199513956 |
| 5 | 1:115217452 | PEV | C/T | 4 | 1 | AMPD1 | p.607G>E | 1.000 | mis | 0.00093 | rs150645738 |
| 5 | 1:144864277 | PEV | G/A | 4 | 1 | PDE4DIP | p.1834R>* | . | stop | 0.007794 | rs139257887 |
| 5 | 1:182569627 | PEV | TG/CA | 3 | 0 | RGS16 | p.137H>C | 0.999 | mis | 0.001279 | . |
| 5 | 1:201190587 | PEV | T/TC | 3 | 0 | IGFN1 | . | . | fs | . | . |
| 5 | 2:31178838 | PEV | C/T | 4 | 0 | GALNT14 | p.163D>N | 1.000 | mis | 0.001047 | rs145021697 |
| 5 | 2:141200150 | PEV | G/A | 4 | 0 | LRP1B | p.3446T>M | 0.990 | mis | . | rs748759183 |
| 5 | 2:204354779 | PEV | T/C | 3 | 1 | RAPH1 | p.87D>G | 1.000 | mis | . | rs756920840 |
| 5 | 2:206590654 | PEV | C/T | 3 | 1 | NRP2 | p.280P>S | 1.000 | mis | 0.00314 | rs79750907 |
| 5 | 2:208986546 | PEV | C/T | 3 | 1 | CRYGD | p.126V>M | 1.000 | mis | 0.00314 | rs150318966 |
| 5 | 3:38163606 | PEV | C/CCAAA | 5 | 0 | DLEC1 | . | . | fs | 0.003744 | rs577236284 |
| 5 | 3:122645347 | PEV | C/T | 4 | 0 | SEMA5B | p.343R>Q | 1.000 | mis | . | rs755257920 |
| 5 | 3:195594962 | PEV | G/C | 3 | 1 | TNK2 | p.799P>R | 1.000 | mis | . | rs557924118 |
| 5 | 4:151186882 | PEV | G/A | 3 | 1 | LRBA | p.2850R>C | 1.000 | mis | 0.001512 | rs145709687 |
| 5 | 6:42946272 | PEV | C/T | 4 | 0 | PEX6 | p.206G>E | 0.993 | mis | 0.000233 | rs139093654 |
| 5 | 6:130166999 | PEV | T/C | 5 | 0 | TMEM244 | . | . | spl | 0 | rs146932263 |
| 5 | 7:100086476 | PEV | C/T | 3 | 1 | NYAP1 | p.378R>W | 0.996 | mis | 0.000117 | rs139880279 |
| 5 | 7:138223420 | PEV | C/T | 4 | 1 | TRIM24 | p.339R>C | 0.999 | mis | . | rs868515585 |
| 5 | 7:150749110 | PEV | C/A | 3 | 1 | ASIC3 | p.449S>R | 1.000 | mis | . | rs777470508 |
| 5 | 9:14792865 | PEV | A/C | 4 | 1 | FREM1 | p.1286M>R | 0.999 | mis | 0.000246 | rs375951503 |
| 5 | 9:21077313 | PEV | G/A | 3 | 1 | IFNB1 | p.186R>* | . | stop | 0.001628 | rs148103065 |
| 5 | 9:117849382 | PEV | C/T | 3 | 1 | TNC | p.210G>S | 0.999 | mis | 0.002907 | rs144032672 |
| 5 | 9:135702392 | PEV | GT/G | 4 | 1 | AK8 | . | . | fs | 0.000363 | rs772875208 |
| 5 | 9:139731827 | PEV | G/A | 5 | 1 | RABL6 | p.281R>H | 0.999 | mis | 0.003917 | rs200704265 |
| 5 | 9:140120188 | PEV | T/A | 3 | 1 | C9orf169 | p.39S>T | 0.995 | mis | . | rs182088466 |
| 5 | 10:96748777 | PEV | C/T | 5 | 1 | CYP2C9 | p.489P>S | 0.999 | mis | 0.002442 | rs9332239 |
| 5 | 10:134017305 | PEV | G/A | 5 | 1 | DPYSL4 | p.501G>R | 1.000 | mis | . | rs61865802 |
| 5 | 11:1016037 | PEV | G/A | 3 | 0 | MUC6 | p.2255T>M | 0.994 | mis | . | rs542942510 |
| 5 | 11:18332420 | PEV | C/T | 3 | 0 | HPS5 | p.1M>I | . | start | 0.00396 | rs149229493 |
| 5 | 11:66031667 | PEV | G/T | 3 | 1 | KLC2 | p.288V>L | 0.996 | mis | . | rs759985873 |
| 5 | 11:101776527 | PEV | A/G | 3 | 1 | ANGPTL5 | p.98L>P | 0.999 | mis | 0.001047 | rs140344944 |
| 5 | 12:6660150 | PEV | G/A | 3 | 1 | IFFO1 | p.264T>M | 0.999 | mis | 0.000116 | rs150862109 |
| 5 | 12:10034237 | PEV | C/T | 3 | 1 | KLRF2 | p.15R>C | 0.990 | mis | . | rs74614163 |
| 5 | 12:53184658 | PEV | T/C | 3 | 1 | KRT3 | . | . | spl | . | rs765471128 |
| 5 | 12:107208919 | PEV | C/T | 4 | 1 | RIC8B | p.193T>M | 0.996 | mis | 0.008837 | rs144718130 |
| 5 | 12:113553461 | PEV | G/A | 4 | 1 | RASAL1 | p.328R>W | 0.997 | mis | 0.002326 | rs35505918 |
| 5 | 13:114435882 | PEV | G/A | 3 | 1 | GRK1 | p.407R>Q | 1.000 | mis | 0.001886 | rs202076986 |
| 5 | 14:104641986 | PEV | C/G | 5 | 1 | KIF26A | p.954P>R | 0.997 | mis | 0.005692 | rs199857970 |
| 5 | 16:2259410 | PEV | G/A | 5 | 1 | MLST8 | . | 1.000 | mis | 0.00314 | rs111384111 |
| 5 | 16:86602433 | PEV | T/C | 3 | 1 | FOXC2 | p.498C>R | 0.998 | mis | 0.001692 | rs61753346 |
| 5 | 17:80016270 | PEV | A/T | 4 | 1 | DUS1L | p.410L>Q | 1.000 | mis | . | . |
| 5 | 19:6753497 | PEV | A/G | 3 | 1 | SH2D3A | p.514F>L | 0.999 | mis | 0.00059 | rs373443344 |
| 5 | X:152822476 | PEV | G/A | 3 | 0 | ATP2B3 | p.810A>T | 0.998 | mis | . | rs782757603 |
| 11 | 1:204198145 | PEV | C/T | 3 | 1 | PLEKHA6 | p.891E>K | 0.991 | mis | . | rs745702745 |
| 11 | 1:227842715 | PEV | A/C | 3 | 0 | ZNF678 | p.310H>P | 1.000 | mis | . | rs752419331 |
| 11 | 1:228566079 | PEV | G/A | 3 | 1 | OBSCN | p.7896G>D | 0.998 | mis | 0.000421 | rs368640713 |
| 11 | 2:28117455 | PEV | C/G | 3 | 1 | BRE | p.11S>C | 0.999 | mis | 0.004302 | rs144572761 |
| 11 | 6:31741041 | PEV | GGC/G | 3 | 1 | VWA7 | . | . | fs | . | . |
| 11 | 6:44116546 | PEV | G/A | 3 | 1 | TMEM63B | p.426R>Q | 0.999 | mis | 0 | rs377671657 |
| 11 | 8:32621349 | PEV | A/G | 3 | 1 | NRG1 | p.406G>G | 0.999 | mis | 0.000698 | rs139436076 |
| 11 | 8:39606929 | PEV | T/C | 3 | 1 | ADAM2 | p.639Y>C | 1.000 | mis | 0.004651 | rs113117613 |
| 11 | 8:104337040 | PEV | T/C | 3 | 1 | FZD6 | p.236Y>H | 1.000 | mis | 0.000116 | rs372850452 |
| 11 | 9:114468926 | PEV | A/G | 3 | 0 | C9orf84 | p.784C>R | 1.000 | mis | . | rs763297176 |
| 11 | 10:13264107 | PEV | G/C | 3 | 1 | UCMA | p.138T>S | 0.999 | mis | 0.00814 | rs140771568 |
| 11 | 12:48597058 | PEV | G/GCTGC | 4 | 1 | OR10AD1 | . | . | fs | . | rs754837553 |
| 11 | 12:52681822 | PEV | A/T | 4 | 1 | KRT81 | p.282Y>* | . | stop | 0.001628 | rs138597671 |
| 11 | 14:88946061 | PEV | G/T | 3 | 1 | PTPN21 | p.572P>T | 0.998 | mis | . | . |
| 11 | 17:4722499 | PEV | G/A | 3 | 0 | PLD2 | p.765R>Q | 0.991 | mis | 0 | rs370990081 |
| 11 | 17:6908625 | PEV | G/A | 3 | 0 | ALOX12 | p.404R>Q | 0.993 | mis | 0.003721 | rs147158964 |
| 11 | 19:39230735 | PEV | G/A | 3 | 1 | CAPN12 | p.229R>C | 0.996 | mis | 0.001512 | rs148454997 |
| 11 | 19:39734673 | PEV | T/C | 3 | 1 | IL28B | p.128H>R | 0.995 | mis | 0.000581 | rs144005418 |
| 11 | 22:22893254 | PEV | G/C | 3 | 1 | PRAME | p.93H>Q | 0.999 | mis | . | rs762099735 |
| 11 | 22:39909520 | PEV | A/C | 3 | 1 | SMCR7L | . | . | spl | . | . |
| 11 | X:38144857 | PEV | T/A | 3 | 1 | RPGR | . | 0.999 | mis | . | . |
| 17 | 1:21009189 | PEV | T/G | 3 | 0 | KIF17 | p.807E>A | 1.000 | mis | 0.001047 | rs115257742 |
| 17 | 1:32201182 | PEV | C/T | 3 | 0 | BAI2 | . | 0.999 | mis | . | rs199560963 |
| 17 | 1:39895727 | PEV | A/T | 3 | 0 | MACF1 | p.3644Q>L | 0.995 | mis | . | rs145430083 |
| 17 | 1:173020073 | PEV | A/T | 3 | 0 | TNFSF18 | p.10C>* | . | stop | 0.004769 | rs185993550 |
| 17 | 1:201047133 | PEV | C/A | 3 | 0 | CACNA1S | p.498R>L | 1.000 | mis | 0.001279 | rs150590855 |
| 17 | 1:228559773 | PEV | G/C | 3 | 1 | OBSCN | p.7098M>I | 0.999 | mis | . | . |
| 17 | 1:248651959 | PEV | C/T | 4 | 1 | OR2T5 | p.24R>* | . | stop | . | rs76332972 |
| 17 | 1:248737512 | PEV | A/G | 3 | 0 | OR2T34 | p.183F>L | 0.994 | mis | 0.004073 | rs148006635 |
| 17 | 2:108488353 | PEV | CT/C | 3 | 1 | RGPD4 | . | . | fs | 0.001217 | rs781025131 |
| 17 | 2:158406865 | PEV | A/G | 3 | 0 | ACVR1C | p.145I>T | 1.000 | mis | 0.001395 | rs56188432 |
| 17 | 3:42906990 | PEV | ATGGCACC/A | 3 | 1 | CCBP2 | . | . | fs | 0.001939 | rs577087357 |
| 17 | 3:157920895 | PEV | C/T | 3 | 0 | RSRC1 | p.119R>C | 1.000 | mis | 0.000349 | rs146246774 |
| 17 | 4:54257221 | PEV | C/T | 3 | 0 | FIP1L1 | p.169T>I | 1.000 | mis | 0.000116 | rs372681089 |
| 17 | 5:31323288 | PEV | C/A | 3 | 1 | CDH6 | p.749S>Y | 1.000 | mis | . | . |
| 17 | 6:169620407 | PEV | C/A | 3 | 0 | THBS2 | p.1133V>F | 0.993 | mis | 0.000581 | rs112533700 |
| 17 | 7:26680344 | PEV | A/G | 3 | 1 | C7orf71 | p.77H>R | 0.999 | mis | 0.000629 | rs372022220 |
| 17 | 7:48556448 | PEV | C/T | 3 | 0 | ABCA13 | p.4590R>W | 1.000 | mis | 0.002413 | rs79049908 |
| 17 | 7:51111278 | PEV | G/A | 3 | 0 | COBL | p.403T>M | 1.000 | mis | . | rs751920839 |
| 17 | 7:151127208 | PEV | A/C | 3 | 0 | CRYGN | p.159S>A | 0.991 | mis | 0.000465 | rs138507328 |
| 17 | 8:32613998 | PEV | A/G | 3 | 0 | NRG1 | p.249Q>Q | 0.997 | mis | . | . |
| 17 | 8:110499005 | PEV | C/T | 3 | 1 | PKHD1L1 | p.3279R>C | 0.994 | mis | 0.002062 | rs189682035 |
| 17 | 9:35089133 | PEV | C/T | 3 | 0 | PIGO | p.659V>M | 0.999 | mis | . | rs753335277 |
| 17 | 10:64913602 | PEV | A/G | 5 | 1 | NRBF2 | p.163D>G | 1.000 | mis | 0.002443 | rs62623680 |
| 17 | 10:85968099 | PEV | G/A | 4 | 1 | CDHR1 | p.378R>Q | 0.999 | mis | 0.002558 | rs140621272 |
| 17 | 11:6653585 | PEV | C/G | 3 | 0 | DCHS1 | p.1053W>S | 0.995 | mis | 0.006867 | rs138340204 |
| 17 | 11:65352971 | PEV | A/C | 3 | 0 | EHBP1L1 | p.1281D>A | 0.999 | mis | 0.000266 | rs377298968 |
| 17 | 11:105950249 | PEV | A/AT | 3 | 0 | AASDHPPT | . | . | fs | 0.000121 | rs769404275 |
| 17 | 11:120996564 | PEV | G/A | 3 | 0 | TECTA | p.586R>Q | 0.990 | mis | 0.000233 | rs146621940 |
| 17 | 11:124266903 | PEV | C/G | 3 | 0 | OR8B3 | p.115L>F | 0.999 | mis | . | . |
| 17 | 11:125766022 | PEV | C/T | 3 | 0 | HYLS1 | . | 0.999 | mis | 0.005466 | rs117878071 |
| 17 | 12:81101832 | PEV | G/T | 3 | 0 | MYF6 | p.112A>S | 1.000 | mis | 0.001395 | rs28928909 |
| 17 | 12:91502161 | PEV | A/G | 3 | 0 | LUM | p.199L>P | 1.000 | mis | 0.004535 | rs147975710 |
| 17 | 14:91155912 | PEV | G/A | 4 | 1 | TTC7B | p.308R>W | 0.994 | mis | 0.000698 | rs143808424 |
| 17 | 14:94931042 | PEV | A/G | 4 | 1 | SERPINA9 | . | . | spl | 0.002454 | rs202070363 |
| 17 | 15:43544956 | PEV | C/A | 5 | 1 | TGM5 | . | . | spl | . | rs773741689 |
| 17 | 15:62360122 | PEV | G/C | 3 | 1 | C2CD4A | p.104G>R | 1.000 | mis | . | rs199710631 |
| 17 | 17:66914289 | PEV | G/C | 4 | 1 | ABCA8 | p.609P>R | 1.000 | mis | 0.001977 | rs144777539 |
| 17 | 19:5908968 | PEV | C/T | 3 | 0 | VMAC | p.109R>C | 0.990 | mis | 0.003605 | rs137868133 |
| 17 | 19:10665971 | PEV | A/C | 3 | 0 | KRI1 | p.564C>G | 1.000 | mis | 0.000116 | rs144763482 |
| 17 | 19:35175765 | PEV | C/T | 5 | 0 | ZNF302 | p.275H>Y | 1.000 | mis | . | rs751655910 |
| 17 | 19:46289934 | PEV | G/A | 3 | 1 | DMWD | p.274R>C | 0.999 | mis | . | rs770581518 |
| 17 | X:2945407 | PEV | C/T | 4 | 1 | ARSH | p.364R>W | 0.999 | mis | . | rs754643657 |
| 17 | X:138899039 | PEV | C/G | 3 | 0 | ATP11C | p.104V>L | 1.000 | mis | . | . |
| 18 | 1:3551827 | PEV | C/T | 4 | 0 | WRAP73 | p.212R>Q | 0.999 | mis | . | rs776582305 |
| 18 | 1:36935300 | PEV | G/C | 4 | 1 | CSF3R | p.476T>S | 0.999 | mis | 0.000116 | rs374610386 |
| 18 | 1:47571862 | PEV | C/G | 4 | 0 | CYP4Z1 | p.377A>G | 0.999 | mis | 0.004419 | rs138601503 |
| 18 | 1:111957178 | PEV | G/A | 3 | 0 | OVGP1 | p.649H>Y | 0.999 | mis | 0.000116 | rs368956930 |
| 18 | 1:145416614 | PEV | G/T | 3 | 0 | HFE2 | p.207G>V | 1.000 | mis | 0.000465 | rs74315323 |
| 18 | 1:183086741 | PEV | A/T | 3 | 0 | LAMC1 | p.587D>V | 0.996 | mis | . | rs776847108 |
| 18 | 2:27308854 | PEV | T/C | 4 | 1 | EMILIN1 | p.1008Y>H | 0.999 | mis | . | . |
| 18 | 2:109527451 | PEV | C/T | 3 | 0 | EDAR | p.203V>I | 0.995 | mis | 0.000349 | rs148212997 |
| 18 | 2:179398050 | PEV | G/A | 3 | 0 | TTN | p.32790T>M | 0.999 | mis | 0.001198 | rs192001910 |
| 18 | 2:219563456 | PEV | C/CT | 3 | 0 | STK36 | . | . | fs | . | . |
| 18 | 3:38052863 | PEV | C/T | 3 | 1 | PLCD1 | p.232R>Q | 1.000 | mis | . | rs765870286 |
| 18 | 5:39289043 | PEV | A/G | 3 | 0 | C9 | p.476I>T | 1.000 | mis | 0.001978 | rs141645272 |
| 18 | 5:112364656 | PEV | C/T | 3 | 1 | MCC | p.952V>M | 0.999 | mis | 0 | rs139859351 |
| 18 | 5:118970218 | PEV | G/A | 3 | 1 | FAM170A | p.212V>I | 0.996 | mis | 0.000119 | rs377559289 |
| 18 | 5:176517963 | PEV | G/A | 3 | 0 | FGFR4 | p.154R>H | 1.000 | mis | . | rs201257476 |
| 18 | 6:146127357 | PEV | T/C | 3 | 0 | FBXO30 | p.62N>S | 1.000 | mis | . | . |
| 18 | 8:37623835 | PEV | T/C | 3 | 1 | PROSC | p.94I>T | 1.000 | mis | . | rs201316839 |
| 18 | 10:24833063 | PEV | C/T | 4 | 1 | KIAA1217 | . | 1.000 | mis | . | . |
| 18 | 10:50901917 | PEV | C/A | 4 | 0 | C10orf53 | p.65C>* | . | stop | 0.002209 | rs145689841 |
| 18 | 10:52005095 | PEV | G/A | 4 | 0 | ASAH2 | p.83Q>* | . | stop | 0.005581 | rs116049719 |
| 18 | 10:55581787 | PEV | T/A | 4 | 0 | PCDH15 | p.1907Y>F | 0.999 | mis | . | . |
| 18 | 11:100912728 | PEV | G/A | 3 | 1 | PGR | p.865S>L | 0.999 | mis | 0 | rs2020880 |
| 18 | 11:101776527 | PEV | A/G | 3 | 1 | ANGPTL5 | p.98L>P | 0.999 | mis | 0.001047 | rs140344944 |
| 18 | 11:111796873 | PEV | T/C | 3 | 1 | C11orf52 | p.108F>L | 1.000 | mis | . | rs587635751 |
| 18 | 11:126326333 | PEV | G/T | 3 | 1 | KIRREL3 | p.253N>K | 0.995 | mis | . | rs763137852 |
| 18 | 13:51825903 | PEV | G/A | 4 | 1 | FAM124A | p.134E>K | 1.000 | mis | . | rs754827186 |
| 18 | 14:63860605 | PEV | T/C | 3 | 1 | PPP2R5E | p.261K>R | 0.999 | mis | . | . |
| 18 | 14:68040543 | PEV | G/T | 4 | 1 | PLEKHH1 | p.622G>V | 1.000 | mis | . | . |
| 18 | 15:52567764 | PEV | T/C | 3 | 1 | MYO5C | p.201T>A | 0.999 | mis | 0.000828 | rs200193628 |
| 18 | 16:81078202 | PEV | T/C | 3 | 1 | ATMIN | p.700F>S | 0.998 | mis | . | . |
| 18 | 17:39120005 | PEV | CA/C | 3 | 0 | KRT39 | . | . | fs | 0.000121 | rs752047814 |
| 18 | 17:56404141 | PEV | T/A | 3 | 1 | BZRAP1 | p.115N>I | 0.999 | mis | . | rs750306324 |
| 18 | 17:74623177 | PEV | C/T | 3 | 0 | ST6GALNAC1 | p.382E>K | 0.997 | mis | . | rs748404010 |
| 18 | 19:9800968 | PEV | AG/A | 4 | 1 | ZNF812 | . | . | fs | . | rs533566052 |
| 18 | 19:41932127 | PEV | G/A | 3 | 0 | B3GNT8 | p.186S>F | 0.998 | mis | 0.003372 | rs137913069 |
| 18 | 21:14982631 | PEV | T/C | 3 | 0 | POTED | p.28C>R | 0.999 | mis | . | rs201709390 |
| 18 | 21:38095423 | PEV | G/A | 3 | 0 | SIM2 | p.179G>R | 1.000 | mis | . | rs767211557 |
| 18 | 21:38132129 | PEV | C/T | 3 | 0 | HLCS | p.565R>Q | 0.991 | mis | . | rs769446135 |
| 19 | 1:13910346 | PEV | C/T | 4 | 0 | PDPN | p.16R>W | 0.999 | mis | 0.006048 | rs113350533 |
| 19 | 1:18961618 | PEV | C/T | 4 | 0 | PAX7 | p.112P>L | 1.000 | mis | 0.002209 | rs142754204 |
| 19 | 1:92649625 | PEV | C/T | 4 | 0 | KIAA1107 | p.1268S>F | 1.000 | mis | . | . |
| 19 | 1:109734349 | PEV | G/A | 4 | 0 | KIAA1324 | . | 0.997 | mis | 0.003372 | rs41279690 |
| 19 | 1:207785051 | PEV | G/A | 3 | 0 | CR1 | p.1659G>S | 0.998 | mis | 0.000242 | rs369023282 |
| 19 | 1:222832796 | PEV | C/T | 3 | 0 | MIA3 | . | 0.999 | mis | . | rs764893679 |
| 19 | 1:223984044 | PEV | T/TA | 3 | 1 | TP53BP2 | . | . | fs | . | . |
| 19 | 1:225533729 | PEV | AT/A | 3 | 0 | DNAH14 | . | . | fs | . | . |
| 19 | 1:225541532 | PEV | CGT/C | 3 | 1 | DNAH14 | . | . | fs | . | . |
| 19 | 1:244745107 | PEV | T/C | 3 | 0 | C1orf101 | p.654F>L | 0.996 | mis | 0.003417 | rs140946008 |
| 19 | 2:27600557 | PEV | T/A | 3 | 1 | ZNF513 | p.432H>L | 1.000 | mis | . | . |
| 19 | 2:170350279 | PEV | A/G | 3 | 1 | BBS5 | p.184N>S | 1.000 | mis | 0.00698 | rs137853921 |
| 19 | 2:179441038 | PEV | C/T | 3 | 1 | TTN | p.21633G>D | 1.000 | mis | 0.002301 | rs201043950 |
| 19 | 3:120315283 | PEV | C/T | 4 | 1 | NDUFB4 | p.26S>F | 1.000 | mis | 0.000466 | rs150031407 |
| 19 | 3:142285014 | PEV | G/A | 4 | 1 | ATR | p.81L>F | 0.999 | mis | 0.000233 | rs147353060 |
| 19 | 3:151171483 | PEV | T/G | 4 | 1 | IGSF10 | p.135H>P | 1.000 | mis | 0.000233 | rs143943953 |
| 19 | 3:169099213 | PEV | G/A | 4 | 1 | MECOM | . | 0.999 | mis | 0.000121 | rs376185183 |
| 19 | 3:197431479 | PEV | G/A | 3 | 0 | KIAA0226 | p.73H>Y | 1.000 | mis | 0.000953 | rs201876836 |
| 19 | 4:186545234 | PEV | G/A | 3 | 1 | SORBS2 | . | 1.000 | mis | . | rs773054747 |
| 19 | 5:54558510 | PEV | G/A | 3 | 0 | DHX29 | p.1259S>F | 1.000 | mis | 0.001047 | rs147947389 |
| 19 | 5:65317206 | PEV | C/T | 3 | 0 | ERBB2IP | p.197T>M | 0.999 | mis | 0.001512 | rs146136641 |
| 19 | 5:176072385 | PEV | G/A | 3 | 1 | EIF4E1B | p.161C>Y | 1.000 | mis | 0.000475 | rs199835250 |
| 19 | 7:36338668 | PEV | C/G | 3 | 0 | EEPD1 | p.521N>K | 0.999 | mis | 0.000116 | rs143061882 |
| 19 | 7:65444841 | PEV | C/T | 3 | 1 | GUSB | p.152D>N | 0.999 | mis | 0.001628 | rs149606212 |
| 19 | 7:99752669 | PEV | C/T | 3 | 0 | C7orf43 | p.570E>K | 0.998 | mis | . | rs546233603 |
| 19 | 7:99798530 | PEV | C/T | 3 | 0 | STAG3 | p.667R>C | 0.999 | mis | 0.001744 | rs141693812 |
| 19 | 7:103061825 | PEV | A/G | 3 | 0 | SLC26A5 | p.46L>P | 1.000 | mis | 0.005116 | rs141952919 |
| 19 | 7:128491324 | PEV | C/T | 3 | 0 | FLNC | p.1827R>C | 0.991 | mis | 0.006211 | rs181067717 |
| 19 | 7:142880936 | PEV | T/C | 3 | 0 | TAS2R39 | p.142F>S | 0.998 | mis | 0.001587 | rs200343853 |
| 19 | 8:16012590 | PEV | C/T | 4 | 0 | MSR1 | p.294G>E | 1.000 | mis | 0.000814 | rs41440349 |
| 19 | 8:109215274 | PEV | A/G | 3 | 0 | EIF3E | p.413F>L | 0.999 | mis | . | rs765274205 |
| 19 | 8:124516924 | PEV | G/A | 3 | 1 | FBXO32 | p.207L>F | 1.000 | mis | 0.000116 | rs369362393 |
| 19 | 8:125568592 | PEV | G/A | 3 | 1 | MTSS1 | p.429R>C | 1.000 | mis | 0.000233 | rs140348524 |
| 19 | 9:108337380 | PEV | T/C | 3 | 0 | FKTN | p.23F>L | 1.000 | mis | . | . |
| 19 | 9:111624712 | PEV | C/T | 3 | 0 | ACTL7A | p.37P>L | 0.999 | mis | 0.000582 | rs149755351 |
| 19 | 9:117788933 | PEV | C/T | 4 | 0 | TNC | p.2071E>K | 1.000 | mis | 0.000349 | rs140573419 |
| 19 | 9:135202325 | PEV | A/C | 4 | 1 | SETX | p.1554C>G | 1.000 | mis | 0.004419 | rs112089123 |
| 19 | 9:140322570 | PEV | C/T | 4 | 1 | NOXA1 | p.116R>W | 1.000 | mis | 0.005036 | rs146657444 |
| 19 | 10:27306678 | PEV | C/T | 3 | 0 | ANKRD26 | p.1419C>Y | 0.993 | mis | 0.002088 | rs146159734 |
| 19 | 10:61819543 | PEV | C/A | 3 | 0 | ANK3 | p.794D>Y | 1.000 | mis | 0.006977 | rs139092048 |
| 19 | 10:96110026 | PEV | CA/C | 3 | 0 | NOC3L | . | . | fs | 0.00206 | rs564137729 |
| 19 | 11:637570 | PEV | C/T | 3 | 0 | DRD4 | p.89P>L | 1.000 | mis | . | rs530031052 |
| 19 | 11:5842283 | PEV | T/A | 3 | 0 | OR52N2 | p.240F>I | 0.991 | mis | 0.005121 | rs61875922 |
| 19 | 11:6341400 | PEV | CGCGGCGC  ACCGCGCG  CTCTTGG/C | 3 | 0 | PRKCDBP | . | . | fs | . | . |
| 19 | 11:33564041 | PEV | G/T | 3 | 1 | KIAA1549L | p.14G>V | 0.992 | mis | . | rs756284803 |
| 19 | 12:68690709 | PEV | CA/C | 3 | 1 | MDM1 | . | . | fs | . | . |
| 19 | 12:124824652 | PEV | G/C | 3 | 1 | NCOR2 | p.1853R>G | 1.000 | mis | 0.000118 | rs373538771 |
| 19 | 13:46924279 | PEV | T/C | 3 | 1 | KIAA0226L | p.513Y>C | 0.999 | mis | . | rs201057946 |
| 19 | 13:99092998 | PEV | C/T | 3 | 0 | FARP1 | p.902R>C | 1.000 | mis | 0.007558 | rs61749894 |
| 19 | 14:21360059 | PEV | C/T | 4 | 1 | RNASE3 | p.72R>C | 0.993 | mis | 0.006628 | rs151169198 |
| 19 | 14:58838672 | PEV | C/G | 4 | 0 | ARID4A | p.1247P>A | 0.997 | mis | . | rs750070120 |
| 19 | 14:92470716 | PEV | T/G | 3 | 0 | TRIP11 | p.1202N>H | 1.000 | mis | 0.003721 | rs41301481 |
| 19 | 14:105353767 | PEV | A/C | 3 | 1 | KIAA0284 | p.1064N>T | 0.999 | mis | 0.002042 | rs200248340 |
| 19 | 15:66857095 | PEV | G/C | 3 | 0 | LCTL | p.67D>E | 1.000 | mis | 0.000116 | rs76150906 |
| 19 | 15:102224322 | PEV | G/A | 3 | 0 | TARSL2 | p.536R>C | 0.999 | mis | 0.000233 | rs34201431 |
| 19 | 16:88498350 | PEV | C/T | 4 | 0 | ZNF469 | p.1463T>M | 0.999 | mis | 0.004714 | rs375045076 |
| 19 | 17:4897720 | PEV | C/A | 3 | 1 | INCA1 | p.5D>Y | 0.999 | mis | 0.000116 | rs201029616 |
| 19 | 17:18862551 | PEV | A/T | 3 | 1 | SLC5A10 | . | 0.999 | mis | 0.002908 | rs148178887 |
| 19 | 17:18881091 | PEV | G/A | 3 | 1 | SLC5A10 | p.630R>W | 0.991 | mis | 0.002869 | rs201046878 |
| 19 | 17:39316732 | PEV | C/A | 4 | 1 | KRTAP4-4 | p.71C>F | 0.992 | mis | . | . |
| 19 | 17:76993649 | PEV | C/T | 3 | 1 | CANT1 | p.19R>Q | 0.999 | mis | 0.001396 | rs144060377 |
| 19 | 19:8491645 | PEV | A/G | 3 | 0 | MARCH2 | p.110H>R | 0.996 | mis | . | rs767726990 |
| 19 | 19:9800915 | PEV | A/G | 3 | 0 | ZNF812 | p.422C>R | 1.000 | mis | . | rs192466418 |
| 19 | 19:19906131 | PEV | T/G | 3 | 0 | ZNF506 | p.189T>P | 1.000 | mis | 0.007772 | rs16996376 |
| 19 | 19:45667499 | PEV | A/T | 4 | 0 | TRAPPC6A | p.107Y>N | 1.000 | mis | 0.003023 | rs142501705 |
| 19 | 20:35414944 | PEV | G/A | 4 | 0 | SOGA1 | p.1644H>Y | 0.999 | mis | . | rs199819154 |
| 19 | 20:43851792 | PEV | C/T | 3 | 0 | SEMG2 | p.507Q>* | . | stop | 0.001279 | rs139726931 |
| 19 | 22:36623523 | PEV | G/A | 3 | 1 | APOL2 | p.314A>V | 1.000 | mis | 0.000116 | rs368449159 |
| 19 | 22:38478793 | PEV | C/T | 3 | 1 | SLC16A8 | p.30G>S | 0.996 | mis | 0.000761 | rs113748161 |
| 74 | 1:2430057 | PEV | C/T | 3 | 1 | PLCH2 | p.774R>C | 0.996 | mis | . | rs755583411 |
| 74 | 1:6536057 | PEV | G/A | 3 | 1 | PLEKHG5 | p.84P>L | 1.000 | mis | 0.000116 | rs143585428 |
| 74 | 1:12423276 | PEV | A/T | 3 | 1 | VPS13D | p.3474K>M | 0.999 | mis | 0.003372 | rs145226038 |
| 74 | 1:22160001 | PEV | C/T | 3 | 0 | HSPG2 | p.3646R>H | 1.000 | mis | 0.004884 | rs112062179 |
| 74 | 1:36893930 | PEV | C/T | 3 | 1 | OSCP1 | . | . | stop | 0.007907 | rs115388124 |
| 74 | 2:31605868 | PEV | G/A | 3 | 0 | XDH | . | 1.000 | mis | 0.000698 | rs142388231 |
| 74 | 2:73195646 | PEV | G/A | 3 | 1 | SFXN5 | p.253A>V | 0.999 | mis | 0.004302 | rs149206069 |
| 74 | 2:107084720 | PEV | C/A | 4 | 1 | RGPD3 | p.9E>* | . | stop | 0.0066 | rs200527401 |
| 74 | 2:152382725 | PEV | G/A | 3 | 1 | NEB | p.7333P>S | 0.991 | mis | . | . |
| 74 | 2:179471784 | PEV | C/G | 3 | 0 | TTN | p.16208V>L | 0.999 | mis | . | rs746831431 |
| 74 | 2:216203632 | PEV | T/A | 3 | 1 | ATIC | . | . | spl | . | . |
| 74 | 2:232393323 | PEV | G/A | 3 | 1 | NMUR1 | p.137R>C | 1.000 | mis | 0.000814 | rs146282412 |
| 74 | 2:242742842 | PEV | A/G | 3 | 1 | GAL3ST2 | p.153Y>C | 1.000 | mis | 0.008025 | rs139344622 |
| 74 | 3:10401618 | PEV | C/T | 3 | 0 | ATP2B2 | p.617D>N | 0.994 | mis | 0.003721 | rs150683478 |
| 74 | 3:134251743 | PEV | G/A | 4 | 1 | CEP63 | . | . | spl | . | rs769414976 |
| 74 | 3:155547508 | PEV | T/G | 3 | 1 | SLC33A1 | p.484N>T | 0.999 | mis | 0.002907 | rs144015992 |
| 74 | 3:167083673 | PEV | C/G | 3 | 1 | ZBBX | . | . | spl | 0.003689 | rs184708397 |
| 74 | 4:15938249 | PEV | TC/T | 4 | 1 | FGFBP1 | . | . | fs | 0.003362 | rs745329431 |
| 74 | 5:110454719 | PEV | A/G | 3 | 1 | WDR36 | p.658D>G | 1.000 | mis | 0.007211 | rs34595252 |
| 74 | 5:140517069 | PEV | C/T | 3 | 1 | PCDHB5 | p.685L>F | 0.997 | mis | 0.002678 | rs139772435 |
| 74 | 5:140531193 | PEV | C/T | 3 | 1 | PCDHB6 | p.452T>I | 0.991 | mis | 0.002793 | rs149392351 |
| 74 | 5:140626618 | PEV | T/C | 3 | 1 | PCDHB15 | p.491L>P | 1.000 | mis | 0.004073 | rs141098531 |
| 74 | 6:155618095 | PEV | C/T | 4 | 1 | TFB1M | p.180V>M | 1.000 | mis | 0.000814 | rs199912560 |
| 74 | 7:2584654 | PEV | C/T | 3 | 0 | BRAT1 | p.107G>R | 1.000 | mis | . | rs749992319 |
| 74 | 10:124249026 | PEV | G/T | 3 | 0 | HTRA1 | p.221V>L | 1.000 | mis | . | . |
| 74 | 11:1258216 | PEV | G/A | 3 | 1 | MUC5B | p.1040R>H | 1.000 | mis | 0 | rs377460516 |
| 74 | 11:107403074 | PEV | C/T | 3 | 1 | ALKBH8 | p.277R>Q | 1.000 | mis | 0.000233 | rs201497980 |
| 74 | 11:114577559 | PEV | G/A | 3 | 1 | NXPE2 | p.529W>* | . | stop | 0.001257 | rs201200145 |
| 74 | 11:123900332 | PEV | G/A | 3 | 0 | OR10G8 | p.1M>I | . | start | . | rs763415946 |
| 74 | 12:22025626 | PEV | T/G | 3 | 0 | ABCC9 | p.711K>Q | 1.000 | mis | . | . |
| 74 | 12:55846851 | PEV | C/T | 4 | 0 | OR6C2 | p.285P>L | 0.999 | mis | . | rs766129652 |
| 74 | 12:121448918 | PEV | T/A | 5 | 0 | C12orf43 | p.59Q>H | 1.000 | mis | 0.004186 | rs112728225 |
| 74 | 13:99109438 | PEV | C/T | 3 | 1 | STK24 | p.415V>M | 0.999 | mis | 0.000233 | rs202009189 |
| 74 | 14:61190027 | PEV | G/T | 3 | 1 | SIX4 | p.256L>M | 1.000 | mis | . | rs746244840 |
| 74 | 16:70954909 | PEV | A/G | 3 | 0 | HYDIN | p.2456F>S | 0.999 | mis | 0.001114 | rs200385024 |
| 74 | 16:71101211 | PEV | G/A | 3 | 1 | HYDIN | p.713A>V | 1.000 | mis | 0.000116 | rs199673743 |
| 74 | 17:7496090 | PEV | G/A | 3 | 1 | FXR2 | p.551R>C | 1.000 | mis | 0.000122 | rs372729860 |
| 74 | 17:33446607 | PEV | C/G | 3 | 1 | RAD51D | p.9C>S | 0.996 | mis | 0.001047 | rs140825795 |
| 74 | 17:72858956 | PEV | G/A | 3 | 1 | FDXR | p.493R>C | 0.993 | mis | 0.000465 | rs143458938 |
| 74 | 19:21991986 | PEV | ATT/A | 5 | 0 | ZNF43 | . | . | fs | 0.000121 | rs761612770 |
| 74 | 19:23542632 | PEV | T/C | 5 | 0 | ZNF91 | p.1050Y>C | 1.000 | mis | 0.000116 | rs202182741 |
| 74 | 19:42265201 | PEV | G/A | 3 | 0 | CEACAM6 | p.157V>M | 0.994 | mis | 0.000116 | rs143159805 |
| 74 | 19:55494283 | PEV | C/G | 3 | 1 | NLRP2 | p.406T>R | 0.999 | mis | 0.009302 | rs139903547 |
| 84 | 1:107867534 | PEV | G/T | 3 | 1 | NTNG1 | p.293V>L | 0.999 | mis | . | . |
| 84 | 1:149902342 | PEV | C/T | 3 | 1 | MTMR11 | p.521R>H | 1.000 | mis | 0.006977 | rs145659444 |
| 84 | 1:179989362 | PEV | A/G | 3 | 1 | CEP350 | p.818Y>C | 0.991 | mis | 0.000349 | rs202204068 |
| 84 | 2:44028807 | PEV | C/T | 3 | 1 | DYNC2LI1 | p.255P>L | 1.000 | mis | . | rs370772363 |
| 84 | 2:113999205 | PEV | C/T | 3 | 1 | PAX8 | p.234E>K | 0.999 | mis | 0.000119 | rs201093713 |
| 84 | 10:5791299 | PEV | A/G | 3 | 0 | FAM208B | p.1972Q>R | 0.998 | mis | 0.000595 | rs200658639 |
| 84 | 17:73089805 | PEV | G/A | 3 | 0 | SLC16A5 | p.25G>D | 0.991 | mis | . | . |
| 113 | 1:39384638 | PEV | C/T | 3 | 1 | RHBDL2 | . | . | spl | 0.001279 | rs150433226 |
| 113 | 1:89414776 | PEV | A/T | 4 | 1 | CCBL2 | . | 0.999 | mis | 0.003256 | rs144984854 |
| 113 | 1:160143962 | PEV | G/C | 3 | 1 | ATP1A4 | p.685D>H | 1.000 | mis | 0.002558 | rs144428770 |
| 113 | 2:53955867 | PEV | T/C | 3 | 1 | ASB3 | p.234S>G | 0.999 | mis | 0 | rs75729621 |
| 113 | 2:54093975 | PEV | C/T | 4 | 1 | PSME4 | p.1769C>Y | 0.998 | mis | . | rs760653754 |
| 113 | 2:58388714 | PEV | A/T | 3 | 1 | FANCL | p.326D>E | 1.000 | mis | 0 | rs140088149 |
| 113 | 3:38739511 | PEV | C/T | 3 | 1 | SCN10A | p.1734E>K | 1.000 | mis | 0.000116 | rs200645452 |
| 113 | 3:38743419 | PEV | C/T | 3 | 1 | SCN10A | p.1523C>Y | 1.000 | mis | 0.002674 | rs142217269 |
| 113 | 3:123337509 | PEV | G/A | 3 | 1 | MYLK | p.1826A>V | 0.999 | mis | 0.000698 | rs147187907 |
| 113 | 4:42127637 | PEV | T/C | 3 | 1 | BEND4 | p.370Q>R | 1.000 | mis | 0.000242 | rs371169633 |
| 113 | 4:62936092 | PEV | C/A | 3 | 1 | LPHN3 | p.1292N>K | 0.999 | mis | . | rs200367437 |
| 113 | 4:71255425 | PEV | C/G | 3 | 1 | SMR3B | p.34P>A | 0.999 | mis | . | rs201478250 |
| 113 | 5:637730 | PEV | C/T | 4 | 1 | CEP72 | p.335R>* | . | stop | . | rs779335926 |
| 113 | 5:71591395 | PEV | G/A | 3 | 0 | MRPS27 | p.82R>W | 0.998 | mis | 0.000233 | rs138419432 |
| 113 | 5:156899406 | PEV | G/A | 3 | 0 | NIPAL4 | p.280R>H | 1.000 | mis | 0.000235 | rs200033975 |
| 113 | 6:148869462 | PEV | G/A | 3 | 1 | SASH1 | p.1171R>Q | 0.999 | mis | 0.002442 | rs143577116 |
| 113 | 8:18432747 | PEV | C/T | 3 | 0 | PSD3 | p.844A>T | 1.000 | mis | . | rs758249050 |
| 113 | 8:48792167 | PEV | A/T | 3 | 0 | PRKDC | p.1707W>R | 0.999 | mis | 0.003484 | rs202110076 |
| 113 | 10:38345409 | PEV | A/G | 3 | 1 | ZNF33A | p.786Q>R | 0.999 | mis | 0.002907 | rs71491230 |
| 113 | 10:50681043 | PEV | G/A | 4 | 1 | ERCC6 | p.914T>M | 1.000 | mis | 0.001395 | rs142580756 |
| 113 | 11:93463070 | PEV | A/T | 4 | 0 | KIAA1731 | p.2534K>N | 0.995 | mis | 0.001257 | rs183120281 |
| 113 | 12:104387185 | PEV | G/A | 3 | 1 | GLT8D2 | p.289H>Y | 1.000 | mis | . | rs764737275 |
| 113 | 12:110350855 | PEV | C/T | 3 | 0 | TCHP | p.371A>V | 0.999 | mis | . | . |
| 113 | 12:133295382 | PEV | C/G | 3 | 1 | PGAM5 | . | 0.999 | mis | 0.001279 | rs17855393 |
| 113 | 14:74522351 | PEV | C/T | 4 | 0 | C14orf45 | p.451R>* | . | stop | 0.000116 | rs372260972 |
| 113 | 14:96730863 | PEV | C/T | 3 | 1 | BDKRB1 | p.282R>* | . | stop | 0.003488 | rs145322761 |
| 113 | 17:641237 | PEV | A/G | 3 | 1 | FAM57A | p.120M>V | 0.996 | mis | . | rs770162346 |
| 113 | 17:4607312 | PEV | C/G | 3 | 0 | PELP1 | p.32R>P | 0.998 | mis | 0.000246 | rs376449838 |
| 113 | 17:10295960 | PEV | G/A | 3 | 1 | MYH8 | . | 1.000 | mis | . | rs530187127 |
| 113 | 17:18395802 | PEV | G/T | 3 | 1 | LGALS9C | p.235G>V | 1.000 | mis | . | . |
| 113 | 17:37900378 | PEV | G/T | 3 | 1 | GRB7 | p.240G>V | 0.990 | mis | 0.00314 | rs145986585 |
| 113 | 17:39037017 | PEV | T/C | 4 | 1 | KRT20 | p.160E>G | 0.999 | mis | 0.005465 | rs148091209 |
| 113 | 19:467664 | PEV | G/A | 3 | 1 | ODF3L2 | p.112R>W | 1.000 | mis | 0.003723 | rs79399119 |
| 113 | 19:5908968 | PEV | C/T | 4 | 1 | VMAC | p.109R>C | 0.990 | mis | 0.003605 | rs137868133 |
| 113 | 19:10748382 | PEV | G/C | 4 | 1 | SLC44A2 | p.550E>Q | 1.000 | mis | 0.00814 | rs142741358 |
| 113 | 19:19905599 | PEV | TC/T | 3 | 1 | ZNF506 | . | . | fs | 0.004874 | rs567754234 |
| 113 | 19:54403579 | PEV | G/A | 3 | 0 | PRKCG | . | . | spl | . | . |
| 113 | 20:2597716 | PEV | A/T | 4 | 1 | TMC2 | . | . | spl | 0.007442 | rs140053393 |
| 113 | 20:51870971 | PEV | T/C | 3 | 1 | TSHZ2 | p.322F>S | 0.998 | mis | . | . |
| 113 | 22:42523558 | PEV | T/C | 3 | 1 | CYP2D6 | p.355Y>C | 1.000 | mis | . | rs202102799 |
| 113 | 22:42968537 | PEV | T/A | 3 | 1 | SERHL2 | . | . | spl | 0.001163 | rs145830635 |
| 113 | 22:50886974 | PEV | G/A | 3 | 1 | SBF1 | . | 0.999 | mis | . | rs750453086 |
| 121 | 2:54036382 | PEV | A/C | 3 | 0 | ERLEC1 | p.358Y>S | 1.000 | mis | . | rs201722051 |
| 121 | 3:10183605 | PEV | C/T | 3 | 1 | VHL | p.25P>L | 0.999 | mis | 0.003655 | rs35460768 |
| 121 | 4:77057438 | PEV | C/G | 3 | 1 | NUP54 | p.141W>C | 0.998 | mis | . | rs200614065 |
| 121 | 5:65105886 | PEV | T/G | 4 | 0 | NLN | p.579I>M | 1.000 | mis | 0.001163 | rs145543140 |
| 121 | 5:150889652 | PEV | C/T | 3 | 1 | FAT2 | p.3997E>K | 0.999 | mis | 0.007093 | rs146458760 |
| 121 | 10:134726261 | PEV | A/C | 3 | 0 | TTC40 | p.799I>M | 1.000 | mis | . | rs200571172 |
| 121 | 11:117105034 | PEV | C/T | 4 | 0 | RNF214 | p.16P>S | 0.999 | mis | 0.004092 | rs139503238 |
| 121 | 13:33703450 | PEV | C/T | 3 | 1 | STARD13 | p.447R>Q | 1.000 | mis | 0.002209 | rs141332232 |
| 121 | 18:47810431 | PEV | G/A | 3 | 1 | CXXC1 | p.420Q>* | . | stop | . | . |
| 121 | 19:6418853 | PEV | G/A | 4 | 1 | KHSRP | p.214R>W | 1.000 | mis | . | . |
| 121 | 19:10088141 | PEV | C/T | 4 | 1 | COL5A3 | p.1045R>H | 0.994 | mis | 0.000233 | rs150379898 |
| 129 | 1:222800921 | PEV | C/T | 3 | 0 | MIA3 | . | 0.999 | mis | 0.003911 | rs193055682 |
| 129 | 3:48666132 | PEV | C/G | 3 | 0 | SLC26A6 | p.518G>R | 1.000 | mis | 0.002245 | rs184187143 |
| 129 | 5:140772573 | PEV | C/T | 3 | 1 | PCDHGA8 | . | 1.000 | mis | 0.002793 | rs182052960 |
| 129 | 7:43547658 | PEV | A/G | 3 | 1 | HECW1 | p.1265N>S | 0.998 | mis | 0.001323 | rs200912368 |
| 129 | 7:99161496 | PEV | CCGCCTTC  CCA/C | 3 | 0 | ZNF655 | . | . | fs | 0.004967 | rs558237705 |
| 129 | 9:127230383 | PEV | C/T | 3 | 1 | GPR144 | p.690R>* | . | stop | . | rs545086542 |
| 129 | 10:70332294 | PEV | C/T | 3 | 1 | TET1 | p.67P>S | 0.999 | mis | . | rs761167266 |
| 129 | 10:78651443 | PEV | C/T | 3 | 1 | KCNMA1 | p.1007R>Q | 1.000 | mis | . | rs764468883 |
| 129 | 11:102033237 | PEV | C/T | 3 | 1 | YAP1 | p.208S>F | 0.996 | mis | . | . |
| 129 | 11:121414325 | PEV | G/A | 3 | 1 | SORL1 | p.585G>D | 0.995 | mis | . | rs563637479 |
| 129 | 12:109889453 | PEV | C/T | 3 | 1 | KCTD10 | p.297V>M | 1.000 | mis | 0.000116 | rs369957720 |
| 129 | 16:21726475 | PEV | T/A | 3 | 0 | OTOA | . | . | spl | . | rs754520650 |
| 129 | 16:58011929 | PEV | C/A | 3 | 1 | TEPP | p.125T>N | 1.000 | mis | . | rs754363887 |
| 129 | 17:7850976 | PEV | A/G | 3 | 0 | CNTROB | p.694E>G | 0.999 | mis | 0.00093 | rs139292572 |
| 129 | 17:46804309 | PEV | C/A | 3 | 0 | HOXB13 | p.233R>L | 0.999 | mis | . | . |
| 129 | 18:47796406 | PEV | T/G | 3 | 1 | MBD1 | . | 0.999 | mis | . | . |
| 129 | 19:3807211 | PEV | T/C | 3 | 1 | ZFR2 | p.868M>V | 0.995 | mis | . | rs764240875 |
| 129 | 19:7706729 | PEV | C/T | 3 | 0 | STXBP2 | p.187R>C | 1.000 | mis | 0.000366 | rs370053399 |
| 129 | 19:15996828 | PEV | G/C | 3 | 0 | CYP4F2 | p.341L>V | 1.000 | mis | 0.007791 | rs145174239 |
| 129 | 19:58232781 | PEV | C/G | 3 | 0 | ZNF671 | p.225G>R | 0.999 | mis | 0.001047 | rs149668420 |
| 129 | 20:48156236 | PEV | C/T | 3 | 0 | PTGIS | p.182G>R | 1.000 | mis | 0.00186 | rs148768155 |
| 129 | X:26157136 | PEV | C/G | 3 | 1 | MAGEB18 | p.12R>G | 0.999 | mis | . | rs760985609 |
| 131 | 5:10227711 | PEV | G/A | 3 | 1 | FAM173B | p.182R>* | . | stop | 0.005053 | rs200658616 |
| 131 | 5:41909884 | PEV | C/A | 3 | 0 | C5orf51 | p.82L>I | 1.000 | mis | 0.005135 | rs142045369 |
| 131 | 5:176861035 | PEV | C/T | 3 | 0 | GRK6 | p.287R>W | 1.000 | mis | . | rs777094286 |
| 131 | 7:92161828 | PEV | G/A | 3 | 1 | RBM48 | p.138R>Q | 1.000 | mis | 0.000122 | rs372855385 |
| 131 | 8:41550195 | PEV | C/T | 3 | 0 | ANK1 | p.1277V>M | 0.999 | mis | 0.001163 | rs148942046 |
| 131 | 8:144999263 | PEV | G/A | 3 | 1 | PLEC | p.1639R>W | 0.997 | mis | . | rs782551115 |
| 131 | 9:37746714 | PEV | C/T | 3 | 0 | FRMPD1 | p.1562T>M | 1.000 | mis | 0.000814 | rs138292555 |
| 131 | 10:67829108 | PEV | T/C | 3 | 0 | CTNNA3 | p.706K>R | 0.995 | mis | . | rs758519231 |
| 131 | 11:1267115 | PEV | C/A | 3 | 0 | MUC5B | p.3002T>K | 1.000 | mis | 0.00414 | rs200554635 |
| 131 | 11:2909582 | PEV | G/T | 3 | 0 | SLC22A18AS | p.197A>E | 0.999 | mis | 0.0044 | rs187086795 |
| 131 | 11:5699642 | PEV | G/C | 3 | 0 | TRIM5 | p.179T>S | 0.999 | mis | . | . |
| 131 | 12:53509433 | PEV | G/C | 3 | 0 | SOAT2 | p.235E>Q | 0.999 | mis | 0.00152 | rs138678118 |
| 131 | 13:96579544 | PEV | C/G | 3 | 0 | UGGT2 | p.675R>T | 0.999 | mis | 0.000815 | rs142975988 |
| 131 | 14:55864124 | PEV | G/A | 3 | 0 | ATG14 | p.84R>* | . | stop | . | . |
| 131 | 14:105959012 | PEV | C/T | 3 | 0 | C14orf80 | p.176R>W | 1.000 | mis | . | rs782457605 |
| 131 | 19:37677213 | PEV | G/C | 3 | 0 | ZNF585B | p.409S>W | 1.000 | mis | 0.000116 | rs368332359 |
| 131 | 20:25277094 | PEV | G/C | 3 | 0 | PYGB | p.823R>P | 0.999 | mis | . | rs758339184 |
| 131 | 20:31606585 | PEV | T/C | 3 | 0 | BPIFB2 | p.271L>P | 1.000 | mis | 0.000116 | rs367964762 |
| 131 | 20:35695185 | PEV | G/C | 3 | 0 | RBL1 | p.263A>G | 1.000 | mis | . | . |
| 131 | 22:32894481 | PEV | GGGTC/G | 3 | 1 | FBXO7 | . | . | fs | 0.000363 | rs772175765 |
| 131 | X:3241246 | PEV | G/C | 3 | 0 | MXRA5 | p.827P>R | 0.999 | mis | 0.004608 | rs41304689 |
| 138 | 2:152483662 | PEV | A/T | 3 | 1 | NEB | p.3401S>T | 0.993 | mis | 0.000719 | rs199847072 |
| 138 | 2:152496910 | PEV | C/T | 3 | 1 | NEB | p.2882D>N | 1.000 | mis | 0.000711 | rs200729207 |
| 138 | 2:168104587 | PEV | G/GA | 3 | 1 | XIRP2 | . | . | fs | . | . |
| 138 | 2:179647785 | PEV | T/C | 3 | 1 | TTN | p.950K>E | 1.000 | mis | 0.000116 | rs367559160 |
| 138 | 2:219735846 | PEV | G/C | 3 | 1 | WNT6 | p.60E>Q | 0.995 | mis | . | . |
| 138 | 3:38753882 | PEV | C/T | 3 | 0 | SCN10A | p.1287V>I | 1.000 | mis | 0.001279 | rs145032037 |
| 138 | 3:47894404 | PEV | G/A | 3 | 0 | MAP4 | p.1096R>W | 0.999 | mis | . | rs770436168 |
| 138 | 3:148711989 | PEV | G/A | 3 | 1 | GYG1 | p.23G>E | 1.000 | mis | 0.000349 | rs112622137 |
| 138 | 4:1066940 | PEV | T/C | 3 | 1 | RNF212 | p.206T>A | 0.990 | mis | 0.000116 | rs371212537 |
| 138 | 5:1219713 | PEV | C/T | 3 | 0 | SLC6A19 | p.491S>F | 1.000 | mis | . | rs763527431 |
| 138 | 5:147499677 | PEV | T/G | 3 | 0 | SPINK5 | p.807C>G | 1.000 | mis | 0.000731 | rs201815688 |
| 138 | 6:12124136 | PEV | C/G | 3 | 0 | HIVEP1 | p.1370Q>E | 0.994 | mis | . | rs751637189 |
| 138 | 6:31122368 | PEV | G/A | 3 | 1 | CCHCR1 | p.200R>C | 1.000 | mis | 0.000185 | rs375819943 |
| 138 | 6:31601179 | PEV | C/T | 4 | 1 | PRRC2A | p.1448P>L | 0.999 | mis | 0.000369 | rs151200532 |
| 138 | 6:31922334 | PEV | C/T | 3 | 1 | RDBP | . | 0.999 | mis | 0.000116 | rs373302915 |
| 138 | 6:144205443 | PEV | G/A | 3 | 1 | ZC2HC1B | . | 1.000 | mis | . | . |
| 138 | 6:146870690 | PEV | T/G | 3 | 1 | RAB32 | p.114F>C | 1.000 | mis | . | . |
| 138 | 6:147527173 | PEV | G/A | 3 | 1 | STXBP5 | p.73A>T | 1.000 | mis | . | rs769656684 |
| 138 | 6:161152819 | PEV | C/T | 3 | 1 | PLG | p.494A>V | 0.997 | mis | 0.00593 | rs4252128 |
| 138 | 9:33466115 | PEV | T/G | 3 | 1 | NOL6 | p.773H>P | 0.993 | mis | . | . |
| 138 | 9:79320991 | PEV | C/T | 4 | 0 | PRUNE2 | p.2067A>T | 0.999 | mis | 0.002931 | rs114221706 |
| 138 | 9:97844938 | PEV | C/T | 3 | 1 | C9orf3 | p.801R>W | 0.999 | mis | 0.001047 | rs145969368 |
| 138 | 10:33196047 | PEV | G/C | 4 | 1 | ITGB1 | . | 0.990 | mis | 0.000233 | rs112423286 |
| 138 | 10:55566719 | PEV | G/A | 4 | 1 | PCDH15 | p.1557R>C | 1.000 | mis | . | rs570828018 |
| 138 | 11:1096498 | PEV | C/T | 3 | 1 | MUC2 | p.2171R>C | 1.000 | mis | 0.002696 | rs185444867 |
| 138 | 11:58919922 | PEV | G/GGCAGA  TACTT | 3 | 1 | FAM111A | p.261*>* | . | fs | 0.006186 | rs533676902 |
| 138 | 11:67186995 | PEV | G/A | 3 | 1 | CARNS1 | p.297R>Q | 0.999 | mis | 0.005148 | rs41302427 |
| 138 | 11:68358142 | PEV | G/A | 3 | 1 | PPP6R3 | p.582R>Q | 0.999 | mis | 0.000699 | rs150095519 |
| 138 | 11:73074409 | PEV | G/A | 3 | 1 | ARHGEF17 | p.1719G>S | 1.000 | mis | 0.001165 | rs143486822 |
| 138 | 11:125867261 | PEV | T/C | 3 | 0 | CDON | p.735T>A | 0.990 | mis | . | rs746853510 |
| 138 | 12:54902264 | PEV | G/A | 4 | 1 | NCKAP1L | p.102R>Q | 0.999 | mis | 0.001395 | rs149360088 |
| 138 | 13:114150005 | PEV | C/T | 3 | 1 | TMCO3 | p.37R>C | 0.999 | mis | 0.000233 | rs372103864 |
| 138 | 16:4935307 | PEV | C/T | 3 | 1 | PPL | p.1117V>M | 1.000 | mis | . | . |
| 138 | 16:68398959 | PEV | C/T | 3 | 1 | SMPD3 | p.454V>I | 1.000 | mis | 0.000349 | rs200582075 |
| 138 | 17:2200602 | PEV | A/G | 3 | 1 | SMG6 | p.665Y>H | 1.000 | mis | . | . |
| 138 | 17:3195207 | PEV | C/T | 4 | 1 | OR3A1 | p.224V>M | 0.999 | mis | 0.003953 | rs142082644 |
| 138 | 17:48774378 | PEV | C/T | 4 | 1 | ANKRD40 | p.295V>M | 0.999 | mis | 0 | rs144124178 |
| 138 | 17:74300534 | PEV | C/T | 3 | 0 | QRICH2 | p.57E>K | 0.999 | mis | 0.000116 | rs72868914 |
| 138 | 17:74681190 | PEV | T/A | 3 | 0 | MXRA7 | p.155K>M | 1.000 | mis | 0.000814 | rs144263669 |
| 138 | 19:9324678 | PEV | G/T | 3 | 1 | OR7D4 | p.279A>D | 0.999 | mis | 0.004767 | rs138510982 |
| 138 | 19:39001390 | PEV | G/A | 4 | 1 | RYR1 | p.3031A>T | 0.999 | mis | . | rs756311466 |
| 138 | 19:51207055 | PEV | G/A | 3 | 0 | SHANK1 | p.419R>W | 0.999 | mis | . | rs755286064 |
| 138 | 19:55685027 | PEV | A/C | 4 | 0 | SYT5 | p.329L>R | 0.995 | mis | . | rs754474231 |
| 138 | 20:34135165 | PEV | C/G | 3 | 1 | ERGIC3 | . | 0.999 | mis | 0.000116 | rs200450712 |
| 138 | 20:35444587 | PEV | G/A | 3 | 1 | SOGA1 | p.420R>C | 1.000 | mis | 0.000466 | rs201715529 |
| 138 | 22:18310511 | PEV | G/A | 3 | 1 | MICAL3 | p.1031A>V | 0.999 | mis | . | rs566915282 |
| 138 | X:77913141 | PEV | A/T | 3 | 0 | ZCCHC5 | p.259Y>* | . | stop | 0.000297 | rs201174792 |
| 138 | X:107976904 | PEV | G/A | 4 | 0 | IRS4 | p.891R>* | . | stop | . | . |
| 198 | 1:1216667 | PEV | G/A | 4 | 1 | SCNN1D | p.23G>S | 0.999 | mis | . | rs751882083 |
| 198 | 1:248813732 | PEV | C/A | 4 | 0 | OR2T27 | p.152G>W | 1.000 | mis | . | . |
| 198 | 3:42956732 | PEV | AT/A | 3 | 0 | ZNF662 | . | . | fs | 0.001212 | rs76978847 |
| 198 | 3:75787546 | PEV | G/C | 3 | 1 | ZNF717 | p.410L>V | 0.999 | mis | . | rs193921040 |
| 198 | 3:170716918 | PEV | AAG/A | 3 | 1 | SLC2A2 | . | . | fs | . | . |
| 198 | 4:37857332 | PEV | A/G | 3 | 1 | PGM2 | p.569Y>C | 1.000 | mis | 0.003023 | rs139469063 |
| 198 | 4:90856063 | PEV | A/G | 3 | 1 | MMRN1 | p.411K>R | 0.997 | mis | 0.000698 | rs141038125 |
| 198 | 6:10874568 | PEV | T/G | 3 | 1 | GCM2 | p.394Y>S | 0.993 | mis | 0.000698 | rs142287570 |
| 198 | 7:81358973 | PEV | C/A | 3 | 1 | HGF | p.330D>Y | 1.000 | mis | 0.000116 | rs5745688 |
| 198 | 7:102246311 | PEV | T/C | 3 | 1 | RASA4 | p.141E>G | 0.998 | mis | . | rs11547189 |
| 198 | 10:29788192 | PEV | G/A | 3 | 1 | SVIL | . | 0.999 | mis | 0.000116 | rs142607117 |
| 198 | 12:112699118 | PEV | C/T | 3 | 0 | HECTD4 | p.811R>H | 0.999 | mis | 0.001395 | rs141477684 |
| 198 | 16:2988334 | PEV | G/A | 3 | 1 | FLYWCH1 | p.642A>T | 1.000 | mis | 0.00108 | rs201029011 |
| 198 | 17:39884458 | PEV | G/A | 4 | 1 | HAP1 | p.407R>W | 1.000 | mis | . | rs573288812 |
| 198 | 19:3743948 | PEV | C/T | 3 | 0 | TJP3 | p.652R>C | 1.000 | mis | 0.003721 | rs150181963 |
| 198 | 19:53643901 | PEV | T/C | 3 | 1 | ZNF347 | p.728H>R | 1.000 | mis | . | . |
| 198 | 19:56369683 | PEV | AGT/A | 4 | 0 | NLRP4 | . | . | fs | . | rs773160721 |
| 198 | 20:36150826 | PEV | A/T | 3 | 1 | BLCAP | . | 0.999 | mis | . | . |
| 1 | 2:163134090 | LNV | C/A | 1 | 2 | IFIH1 | p.627E>* | . | stop | 0.005581 | rs35744605 |
| 1 | 2:179411207 | LNV | A/T | 1 | 2 | TTN | p.29976D>E | 1.000 | mis | 0.003499 | rs72648256 |
| 1 | 2:179454460 | LNV | G/C | 0 | 2 | TTN | p.19023N>K | 0.999 | mis | 0.000244 | rs376455983 |
| 1 | 2:179579256 | LNV | C/T | 1 | 2 | TTN | p.8432V>I | 0.999 | mis | 0.001339 | rs16866457 |
| 1 | 2:179600303 | LNV | G/C | 1 | 2 | TTN | p.4640T>S | 0.999 | mis | 0.003536 | rs72648925 |
| 1 | 2:198498440 | LNV | A/G | 1 | 2 | RFTN2 | . | . | spl | . | . |
| 1 | 2:204037504 | LNV | C/T | 0 | 2 | NBEAL1 | p.2055P>L | 1.000 | mis | . | rs201170321 |
| 1 | 4:110758746 | LNV | G/C | 1 | 2 | RRH | p.235Q>H | 1.000 | mis | . | rs766125744 |
| 1 | 5:89949605 | LNV | C/T | 1 | 2 | GPR98 | p.1405S>F | 1.000 | mis | 0.001583 | rs41305898 |
| 1 | 7:99917237 | LNV | A/T | 0 | 3 | SPDYE3 | p.466I>F | 0.999 | mis | 0.006744 | rs200892093 |
| 1 | 7:103175928 | LNV | A/G | 0 | 2 | RELN | . | 1.000 | mis | . | . |
| 1 | 8:95448769 | LNV | G/A | 0 | 2 | RAD54B | . | . | stop | . | rs779188605 |
| 1 | 9:117849382 | LNV | C/T | 1 | 2 | TNC | p.210G>S | 0.999 | mis | 0.002907 | rs144032672 |
| 1 | 9:131196825 | LNV | TCA/T | 1 | 2 | CERCAM | . | . | fs | . | rs761057114 |
| 1 | 9:134353984 | LNV | G/A | 0 | 2 | PRRC2B | p.1579R>K | 0.998 | mis | 0.000732 | rs199819277 |
| 1 | 10:18789874 | LNV | C/T | 0 | 2 | CACNB2 | . | 1.000 | mis | 0.001395 | rs150528041 |
| 1 | 11:1275472 | LNV | C/T | 1 | 2 | MUC5B | p.5123A>V | 0.999 | mis | 0.001637 | rs201591679 |
| 1 | 11:6640043 | LNV | C/T | 1 | 2 | TPP1 | p.65V>M | 0.996 | mis | . | . |
| 1 | 14:91779704 | LNV | T/C | 1 | 2 | CCDC88C | p.819D>G | 0.999 | mis | 0.00295 | rs61743881 |
| 1 | 15:50264839 | LNV | C/T | 1 | 2 | ATP8B4 | p.395G>S | 1.000 | mis | 0.0078 | rs138799625 |
| 1 | 15:86205618 | LNV | G/A | 1 | 2 | AKAP13 | . | . | spl | 0.000233 | rs146891785 |
| 1 | 19:1057343 | LNV | G/A | 1 | 3 | ABCA7 | p.1599V>M | 1.000 | mis | 0.003605 | rs117187003 |
| 1 | 19:6183265 | LNV | AT/A | 1 | 2 | ACSBG2 | . | . | fs | 0.000121 | rs769118076 |
| 1 | 19:22575784 | LNV | C/A | 1 | 2 | ZNF98 | . | . | spl | . | rs753118485 |
| 1 | 21:30699652 | LNV | G/A | 1 | 2 | BACH1 | p.503D>N | 0.999 | mis | . | . |
| 2 | 2:179444915 | LNV | A/G | 0 | 2 | TTN | p.20726S>P | 0.999 | mis | 0.001833 | rs72646873 |
| 2 | 2:220418334 | LNV | C/A | 0 | 2 | OBSL1 | p.1651E>* | . | stop | 0.001307 | rs140825693 |
| 2 | 4:124177315 | LNV | G/T | 1 | 2 | SPATA5 | p.829D>Y | 0.999 | mis | 0.005465 | rs35206443 |
| 2 | 6:35088096 | LNV | G/A | 1 | 2 | TCP11 | p.332R>W | 0.999 | mis | 0.000116 | rs199729611 |
| 2 | 6:66005773 | LNV | C/A | 1 | 2 | EYS | p.669C>F | 0.999 | mis | . | rs780193024 |
| 2 | 7:47408825 | LNV | C/T | 1 | 1 | TNS3 | p.473R>Q | 0.999 | mis | . | rs770107569 |
| 2 | 8:21978273 | LNV | G/A | 1 | 1 | HR | p.856R>W | 0.999 | mis | 0.001429 | rs377059643 |
| 2 | 8:100147957 | LNV | A/G | 1 | 1 | VPS13B | . | 1.000 | mis | 0.00093 | rs143205296 |
| 2 | 10:115528650 | LNV | G/T | 0 | 2 | PLEKHS1 | . | . | spl | . | rs770866572 |
| 2 | 11:224194 | LNV | C/T | 1 | 2 | SIRT3 | p.143G>S | 0.999 | mis | 0.002209 | rs147722093 |
| 2 | 15:62211598 | LNV | G/A | 1 | 1 | VPS13C | p.2510R>W | 1.000 | mis | . | . |
| 2 | 16:89881011 | LNV | G/T | 1 | 1 | FANCA | p.67P>Q | 0.991 | mis | 0.000233 | rs200698961 |
| 2 | 17:26691490 | LNV | ACACTGA  GGTG/A | 1 | 1 | SEBOX | . | . | fs | 0.001122 | rs782103897 |
| 2 | 19:35505271 | LNV | C/G | 1 | 2 | GRAMD1A | p.343S>C | 1.000 | mis | . | . |
| 2 | 19:52394614 | LNV | C/G | 1 | 2 | ZNF649 | p.259E>Q | 0.999 | mis | 0.002558 | rs149982418 |
| 2 | 20:40033870 | LNV | G/A | 1 | 1 | CHD6 | p.2504T>M | 0.999 | mis | 0.000116 | rs150243429 |
| 2 | 22:24919586 | LNV | G/A | 1 | 1 | UPB1 | . | . | spl | 0.002907 | rs143493067 |
| 2 | X:47774873 | LNV | T/G | 1 | 1 | ZNF81 | p.276C>W | 0.999 | mis | 0.000623 | rs202107980 |
| 2 | X:130678865 | LNV | T/A | 1 | 1 | OR13H1 | p.273I>N | 1.000 | mis | 0.000149 | rs149043680 |
| 5 | 1:227843254 | LNV | T/G | 0 | 2 | ZNF678 | p.490C>G | 1.000 | mis | 0 | rs142439512 |
| 5 | 2:86257417 | LNV | G/A | 1 | 1 | POLR1A | p.1561R>W | 1.000 | mis | 0.000121 | rs185258869 |
| 5 | 2:179569009 | LNV | G/A | 1 | 1 | TTN | p.9713P>S | 0.999 | mis | 0.000122 | rs368531555 |
| 5 | 2:186670068 | LNV | A/C | 1 | 1 | FSIP2 | p.5434K>N | 0.999 | mis | . | . |
| 5 | 2:186673618 | LNV | C/A | 1 | 1 | FSIP2 | p.6618P>T | 0.990 | mis | 0.002711 | rs148575803 |
| 5 | 2:197594112 | LNV | G/A | 1 | 1 | CCDC150 | . | . | spl | . | rs774382254 |
| 5 | 3:32587449 | LNV | C/T | 1 | 2 | DYNC1LI1 | p.77G>R | 0.995 | mis | 0.002791 | rs143775988 |
| 5 | 3:98217461 | LNV | A/T | 1 | 1 | OR5K2 | p.313S>C | 0.990 | mis | . | . |
| 5 | 4:110932390 | LNV | C/T | 1 | 2 | EGF | p.1094P>S | 0.991 | mis | . | rs780451968 |
| 5 | 5:124036811 | LNV | T/C | 1 | 1 | ZNF608 | p.353N>S | 0.998 | mis | . | rs201318688 |
| 5 | 5:140626707 | LNV | T/C | 1 | 1 | PCDHB15 | p.521Y>H | 1.000 | mis | 0.004535 | rs148237600 |
| 5 | 6:7584060 | LNV | C/T | 1 | 2 | DSP | p.1590R>W | 0.999 | mis | . | rs767661570 |
| 5 | 6:30153820 | LNV | G/A | 1 | 2 | TRIM26 | p.485R>W | 0.998 | mis | . | . |
| 5 | 6:31595926 | LNV | C/T | 1 | 2 | PRRC2A | p.559P>S | 0.999 | mis | 0.004061 | rs45544132 |
| 5 | 6:32974914 | LNV | C/A | 1 | 2 | HLA-DOA | p.231G>V | 0.999 | mis | 0.00186 | rs141887419 |
| 5 | 6:33405643 | LNV | C/T | 1 | 2 | SYNGAP1 | p.321R>C | 1.000 | mis | . | . |
| 5 | 6:109830382 | LNV | C/A | 0 | 2 | AKD1 | . | 0.999 | mis | . | rs567363215 |
| 5 | 6:119332590 | LNV | C/T | 0 | 2 | FAM184A | p.393E>K | 0.999 | mis | . | rs760594623 |
| 5 | 6:129950497 | LNV | C/A | 0 | 2 | ARHGAP18 | . | . | spl | 0.000116 | rs138161171 |
| 5 | 7:1131378 | LNV | C/T | 1 | 1 | C7orf50 | p.5S>F | 0.999 | mis | 0.004767 | rs117290655 |
| 5 | 7:4824629 | LNV | G/A | 1 | 1 | AP5Z1 | p.294R>Q | 0.996 | mis | 0.003172 | rs200957609 |
| 5 | 7:34125622 | LNV | C/T | 1 | 1 | BMPER | p.555R>W | 0.992 | mis | 0.009535 | rs10249320 |
| 5 | 7:55902239 | LNV | G/A | 1 | 2 | SEPT14 | p.200S>F | 1.000 | mis | 0.005233 | rs190406908 |
| 5 | 7:122027130 | LNV | C/T | 1 | 2 | CADPS2 | p.1042D>N | 1.000 | mis | 0.003378 | rs76528953 |
| 5 | 7:140058450 | LNV | C/A | 1 | 1 | SLC37A3 | p.171G>W | 1.000 | mis | 0.000116 | rs368497699 |
| 5 | 8:8749944 | LNV | G/T | 1 | 1 | MFHAS1 | p.209L>M | 1.000 | mis | 0.002327 | rs147391131 |
| 5 | 8:23167180 | LNV | C/T | 0 | 2 | LOXL2 | . | . | spl | . | rs144719588 |
| 5 | 9:138415731 | LNV | G/C | 1 | 1 | LCN1 | p.100G>R | 0.999 | mis | 0.004419 | rs117638349 |
| 5 | 9:140110748 | LNV | C/T | 1 | 1 | NDOR1 | p.567D>D | 0.999 | mis | 0.001395 | rs145104982 |
| 5 | 11:34194814 | LNV | C/T | 1 | 1 | ABTB2 | p.429V>M | 1.000 | mis | . | rs565986248 |
| 5 | 11:62292156 | LNV | C/T | 1 | 2 | AHNAK | p.3245E>K | 0.992 | mis | 0.000349 | rs142811323 |
| 5 | 11:64820738 | LNV | C/T | 1 | 1 | NAALADL1 | p.384A>T | 0.999 | mis | 0.000353 | rs148772939 |
| 5 | 11:65650731 | LNV | C/T | 1 | 1 | CTSW | p.286P>S | 0.996 | mis | 0.000233 | rs202134820 |
| 5 | 11:76885871 | LNV | C/T | 1 | 2 | MYO7A | p.669R>* | . | stop | 0.00012 | rs111033201 |
| 5 | 11:108160410 | LNV | A/T | 1 | 1 | ATM | p.1440K>* | . | stop | . | . |
| 5 | 12:52822067 | LNV | C/T | 1 | 1 | KRT75 | p.452R>H | 1.000 | mis | 0 | rs141902697 |
| 5 | 12:100926308 | LNV | T/C | 1 | 1 | NR1H4 | p.173M>T | 0.995 | mis | 0.00686 | rs61755050 |
| 5 | 14:20837854 | LNV | T/C | 1 | 1 | TEP1 | p.2468T>A | 1.000 | mis | 0.002326 | rs79167882 |
| 5 | 14:23235822 | LNV | G/C | 1 | 1 | OXA1L | p.31W>S | 0.999 | mis | 0.003256 | rs143108324 |
| 5 | 14:68260427 | LNV | CA/C | 1 | 2 | ZFYVE26 | . | . | fs | . | rs768176054 |
| 5 | 15:42175195 | LNV | G/C | 1 | 1 | SPTBN5 | . | 1.000 | mis | . | rs774744727 |
| 5 | 15:42178008 | LNV | C/T | 1 | 1 | SPTBN5 | p.447R>H | 1.000 | mis | 0.000714 | rs199807959 |
| 5 | 15:75014049 | LNV | G/A | 1 | 1 | CYP1A1 | p.279R>W | 1.000 | mis | 0 | rs34260157 |
| 5 | 16:138725 | LNV | T/A | 1 | 1 | NPRL3 | p.326R>S | 0.996 | mis | 0.001995 | rs202015937 |
| 5 | 16:422261 | LNV | C/T | 1 | 2 | TMEM8A | p.681R>H | 0.999 | mis | 0 | rs148676257 |
| 5 | 16:775236 | LNV | C/T | 1 | 2 | CCDC78 | . | . | spl | 0.001513 | rs138669350 |
| 5 | 16:71101211 | LNV | G/A | 1 | 1 | HYDIN | p.713A>V | 1.000 | mis | 0.000116 | rs199673743 |
| 5 | 17:36963120 | LNV | G/A | 1 | 2 | CWC25 | p.267P>L | 0.997 | mis | . | rs562372884 |
| 5 | 19:6375607 | LNV | G/A | 1 | 2 | PSPN | p.57R>C | 0.999 | mis | . | rs761239429 |
| 5 | 19:7964482 | LNV | G/A | 1 | 2 | LRRC8E | p.359V>I | 0.999 | mis | 0.002442 | rs45584934 |
| 5 | 19:14070018 | LNV | G/A | 1 | 2 | DCAF15 | p.316A>T | 1.000 | mis | . | rs748698814 |
| 5 | 19:41086245 | LNV | G/A | 1 | 2 | SHKBP1 | p.139R>Q | 0.991 | mis | 0.001512 | rs146113659 |
| 5 | 20:2781100 | LNV | G/A | 0 | 2 | CPXM1 | p.40S>L | 0.992 | mis | 0.001371 | rs17855829 |
| 5 | 20:33588634 | LNV | G/A | 1 | 1 | MYH7B | p.1790V>M | 0.999 | mis | 0.000118 | rs375532662 |
| 5 | 21:47850147 | LNV | G/T | 1 | 1 | PCNT | . | . | spl | . | . |
| 5 | 22:32000307 | LNV | C/T | 1 | 1 | SFI1 | p.632R>W | 0.999 | mis | . | rs117137547 |
| 5 | X:15607508 | LNV | G/A | 1 | 1 | ACE2 | p.219R>C | 0.998 | mis | 0.000446 | rs372272603 |
| 5 | X:37027007 | LNV | C/A | 1 | 1 | FAM47C | p.175P>H | 1.000 | mis | 0.005202 | rs28706806 |
| 5 | X:49114806 | LNV | C/T | 1 | 2 | FOXP3 | p.53G>R | 1.000 | mis | . | rs587780340 |
| 5 | X:151128431 | LNV | C/T | 1 | 2 | GABRE | p.222E>K | 0.998 | mis | 0 | rs374796522 |
| 11 | 1:1354865 | LNV | G/A | 1 | 1 | ANKRD65 | p.272A>V | 0.999 | mis | . | . |
| 11 | 1:15642964 | LNV | G/A | 1 | 1 | FHAD1 | . | . | spl | 0.0022 | rs201462752 |
| 11 | 1:89414776 | LNV | A/T | 1 | 1 | CCBL2 | . | 0.999 | mis | 0.003256 | rs144984854 |
| 11 | 1:145304544 | LNV | C/A | 1 | 1 | NBPF10 | p.493Q>K | 1.000 | mis | . | rs781964907 |
| 11 | 1:155290261 | LNV | T/A | 1 | 1 | FDPS | . | 1.000 | mis | 0.002674 | rs61740152 |
| 11 | 2:136627912 | LNV | G/A | 1 | 1 | MCM6 | p.92R>W | 0.993 | mis | 0.001395 | rs61752701 |
| 11 | 2:241534088 | LNV | C/T | 1 | 1 | CAPN10 | p.320P>L | 0.999 | mis | 0.00186 | rs140681762 |
| 11 | 2:242289584 | LNV | G/A | 1 | 1 | SEPT2 | p.361V>M | 0.999 | mis | 0.002209 | rs149773653 |
| 11 | 3:72866431 | LNV | TATCA/T | 1 | 1 | SHQ1 | . | . | fs | . | rs571329853 |
| 11 | 3:75787546 | LNV | G/C | 1 | 1 | ZNF717 | p.410L>V | 0.999 | mis | . | rs193921040 |
| 11 | 3:100057941 | LNV | G/C | 1 | 1 | NIT2 | p.6L>F | 0.999 | mis | 0 | rs369021422 |
| 11 | 4:952009 | LNV | C/T | 1 | 1 | TMEM175 | p.414R>W | 0.999 | mis | 0.005814 | rs140597786 |
| 11 | 4:5527185 | LNV | T/C | 1 | 1 | C4orf6 | p.43I>T | 0.999 | mis | 0.006047 | rs147966579 |
| 11 | 4:47647212 | LNV | C/G | 1 | 1 | CORIN | . | . | spl | . | . |
| 11 | 4:113359739 | LNV | C/G | 1 | 1 | ALPK1 | p.1096N>K | 1.000 | mis | 0.000116 | rs368356690 |
| 11 | 4:154625118 | LNV | T/A | 1 | 1 | TLR2 | p.353C>* | . | stop | . | rs200805994 |
| 11 | 5:837553 | LNV | C/G | 1 | 1 | ZDHHC11 | p.276R>P | 0.999 | mis | . | rs528116435 |
| 11 | 5:1244729 | LNV | C/A | 1 | 1 | SLC6A18 | p.501C>* | . | stop | 0 | rs147945040 |
| 11 | 6:151266675 | LNV | AAAGT/A | 1 | 1 | MTHFD1L | . | . | fs | . | . |
| 11 | 7:47870811 | LNV | TCA/T | 1 | 1 | PKD1L1 | . | . | spl | 0.000848 | rs528302390 |
| 11 | 8:2024286 | LNV | C/T | 1 | 2 | MYOM2 | p.396R>W | 1.000 | mis | 0 | rs370886250 |
| 11 | 9:125642086 | LNV | C/T | 1 | 1 | RC3H2 | p.387G>D | 1.000 | mis | 0.000122 | rs374882710 |
| 11 | 10:51769514 | LNV | C/G | 1 | 1 | AGAP6 | p.543I>M | 0.999 | mis | . | rs782029003 |
| 11 | 10:106675638 | LNV | G/A | 1 | 1 | SORCS3 | p.248G>D | 0.997 | mis | . | . |
| 11 | 11:1857434 | LNV | A/G | 1 | 1 | SYT8 | p.160T>A | 0.999 | mis | 0.000815 | rs148291586 |
| 11 | 12:124359953 | LNV | C/T | 1 | 1 | DNAH10 | p.2587S>L | 1.000 | mis | . | rs751315749 |
| 11 | 13:47262065 | LNV | T/C | 1 | 1 | LRCH1 | p.301Y>H | 0.999 | mis | 0 | rs201406758 |
| 11 | 13:61989290 | LNV | A/G | 1 | 1 | PCDH20 | p.1M>T | . | start | . | rs182750134 |
| 11 | 14:23312489 | LNV | G/T | 1 | 1 | MMP14 | p.238V>L | 0.998 | mis | . | rs200519097 |
| 11 | 14:31858118 | LNV | C/T | 1 | 1 | HEATR5A | p.289R>Q | 1.000 | mis | 0.002063 | rs374697149 |
| 11 | 16:705854 | LNV | G/A | 1 | 1 | WDR90 | p.644W>* | . | stop | 0.000823 | rs201829989 |
| 11 | 16:2523111 | LNV | C/T | 1 | 1 | NTN3 | p.415R>C | 0.999 | mis | 0.002675 | rs141616597 |
| 11 | 16:68855966 | LNV | G/A | 1 | 1 | CDH1 | p.592A>T | 0.999 | mis | 0.006279 | rs35187787 |
| 11 | 16:88695167 | LNV | C/T | 1 | 1 | ZC3H18 | . | 0.998 | mis | 0.000814 | rs144953625 |
| 11 | 17:3594990 | LNV | G/A | 1 | 1 | P2RX5 | p.79S>L | 0.995 | mis | 0.001395 | rs142264131 |
| 11 | 17:10222371 | LNV | A/T | 1 | 1 | MYH13 | p.1158S>R | 0.999 | mis | . | rs781663301 |
| 11 | 17:39506824 | LNV | G/A | 1 | 1 | KRT33A | p.66R>C | 1.000 | mis | 0.004884 | rs140696036 |
| 11 | 17:48704028 | LNV | C/G | 1 | 1 | CACNA1G | p.2287D>E | 0.992 | mis | 0.000485 | rs199695347 |
| 11 | 17:67081218 | LNV | C/T | 1 | 1 | ABCA6 | p.1379A>T | 1.000 | mis | 0.004419 | rs149025377 |
| 11 | 17:79418882 | LNV | C/T | 1 | 1 | BAHCC1 | p.1334P>S | 0.993 | mis | 0.000966 | rs202119784 |
| 11 | 17:79867425 | LNV | A/G | 1 | 1 | PCYT2 | p.48M>T | 0.999 | mis | 0.007909 | rs150714189 |
| 11 | 17:80443425 | LNV | C/G | 1 | 1 | NARF | p.283R>G | 0.990 | mis | 0.000116 | rs368049140 |
| 11 | 18:3879402 | LNV | T/A | 1 | 1 | DLGAP1 | p.223T>S | 0.996 | mis | . | . |
| 11 | 18:5397364 | LNV | G/A | 1 | 1 | EPB41L3 | p.845P>L | 0.999 | mis | 0.00186 | rs140230336 |
| 11 | 18:29049106 | LNV | T/A | 1 | 1 | DSG3 | p.564I>N | 1.000 | mis | . | rs763048097 |
| 11 | 18:56204943 | LNV | G/T | 1 | 1 | ALPK2 | p.826P>T | 0.990 | mis | 0.000698 | rs139158112 |
| 11 | 19:8661989 | LNV | C/T | 1 | 1 | ADAMTS10 | p.308G>R | 0.999 | mis | 0.000233 | rs372881047 |
| 11 | 19:9069568 | LNV | G/A | 1 | 1 | MUC16 | p.5960L>F | 0.999 | mis | 0.000241 | rs200231859 |
| 11 | 19:17397353 | LNV | C/G | 1 | 1 | ANKLE1 | p.614R>G | 0.999 | mis | 0.004193 | rs137870697 |
| 11 | 19:19030147 | LNV | G/A | 1 | 1 | COPE | p.4P>L | 0.999 | mis | . | rs777733336 |
| 11 | 19:56011423 | LNV | C/T | 1 | 1 | SSC5D | p.649T>M | 0.992 | mis | . | . |
| 11 | 21:34877982 | LNV | G/A | 1 | 1 | GART | p.871R>C | 1.000 | mis | . | rs746209819 |
| 11 | 21:42770896 | LNV | G/A | 1 | 1 | MX2 | p.408G>R | 1.000 | mis | 0.006977 | rs141083003 |
| 17 | 2:10904471 | LNV | T/G | 1 | 1 | ATP6V1C2 | p.100F>V | 0.999 | mis | . | rs780967250 |
| 17 | 2:153486180 | LNV | C/T | 1 | 1 | FMNL2 | p.804A>V | 0.997 | mis | 0.000123 | rs201793844 |
| 17 | 3:49321424 | LNV | T/C | 1 | 1 | USP4 | . | 0.999 | mis | 0.00686 | rs41290700 |
| 17 | 3:50402159 | LNV | G/A | 1 | 1 | CACNA2D2 | p.1126L>F | 0.999 | mis | 0.000715 | rs147278705 |
| 17 | 3:197427741 | LNV | C/T | 1 | 1 | KIAA0226 | p.275R>Q | 0.999 | mis | . | rs774252330 |
| 17 | 4:71701950 | LNV | C/T | 1 | 1 | GRSF1 | . | 0.999 | mis | 0.001097 | rs201196367 |
| 17 | 4:123185562 | LNV | G/T | 1 | 1 | KIAA1109 | p.2433V>F | 1.000 | mis | . | . |
| 17 | 6:31948505 | LNV | C/T | 1 | 1 | STK19 | p.326R>W | 1.000 | mis | . | . |
| 17 | 6:33052871 | LNV | T/G | 1 | 1 | HLA-DPB1 | p.170V>G | 0.999 | mis | . | rs751221886 |
| 17 | 6:35922961 | LNV | C/T | 1 | 1 | SLC26A8 | p.734V>M | 1.000 | mis | 0.000116 | rs138344479 |
| 17 | 6:39865007 | LNV | T/C | 1 | 1 | DAAM2 | p.856I>T | 0.999 | mis | 0.00614 | rs61748650 |
| 17 | 6:117113761 | LNV | A/AGG | 1 | 1 | GPRC6A | . | . | fs | . | rs550458778 |
| 17 | 7:101256959 | LNV | G/A | 1 | 1 | MYL10 | p.189R>C | 1.000 | mis | 0.000349 | rs141873317 |
| 17 | 7:102246311 | LNV | T/C | 1 | 1 | RASA4 | p.141E>G | 0.998 | mis | . | rs11547189 |
| 17 | 7:150171599 | LNV | CAGATATAG  TGCCTTCAAC  TACCGGGCAA  CAGGAGA/C | 1 | 1 | GIMAP8 | . | . | fs | . | rs536291210 |
| 17 | 8:20008178 | LNV | G/A | 1 | 1 | SLC18A1 | . | 1.000 | mis | 0.00093 | rs138845247 |
| 17 | 8:69017509 | LNV | T/TAGGA | 1 | 1 | PREX2 | . | . | fs | . | rs774880361 |
| 17 | 8:75757618 | LNV | T/C | 1 | 1 | PI15 | . | 0.998 | mis | 0.000698 | rs144157830 |
| 17 | 8:81399152 | LNV | C/T | 1 | 1 | ZBTB10 | p.36S>L | 0.999 | mis | . | rs201355421 |
| 17 | 8:98991158 | LNV | T/C | 1 | 1 | MATN2 | p.335C>R | 1.000 | mis | 0.001195 | rs184482356 |
| 17 | 8:125078752 | LNV | G/T | 1 | 1 | FER1L6 | p.1213W>C | 1.000 | mis | . | . |
| 17 | 8:145577161 | LNV | G/C | 1 | 1 | TMEM249 | p.185I>M | 0.999 | mis | . | rs782550880 |
| 17 | 9:6250546 | LNV | A/G | 1 | 1 | IL33 | p.55K>R | 0.999 | mis | 0.000349 | rs138513976 |
| 17 | 10:23257281 | LNV | G/A | 1 | 1 | ARMC3 | p.260C>Y | 1.000 | mis | . | . |
| 17 | 10:55581785 | LNV | T/C | 1 | 1 | PCDH15 | p.1908S>G | 0.999 | mis | . | rs763251067 |
| 17 | 11:65351863 | LNV | A/G | 1 | 1 | EHBP1L1 | p.1082D>G | 1.000 | mis | 0.000706 | rs185664667 |
| 17 | 11:65810834 | LNV | T/C | 1 | 1 | GAL3ST3 | p.147Y>C | 0.999 | mis | . | rs200319398 |
| 17 | 12:21445230 | LNV | G/T | 1 | 1 | SLCO1A2 | p.493S>* | . | stop | . | rs746068777 |
| 17 | 12:114390349 | LNV | C/T | 1 | 1 | RBM19 | p.323R>Q | 0.998 | mis | . | rs765861178 |
| 17 | 12:124415916 | LNV | C/G | 1 | 1 | DNAH10 | p.4153N>K | 0.997 | mis | 0.001893 | rs200478023 |
| 17 | 13:20244421 | LNV | A/G | 1 | 1 | MPHOSPH8 | p.792K>R | 1.000 | mis | 0.000116 | rs138439899 |
| 17 | 14:24041061 | LNV | C/T | 1 | 1 | JPH4 | p.407R>Q | 0.990 | mis | . | rs201428906 |
| 17 | 14:64691206 | LNV | G/A | 1 | 1 | SYNE2 | p.6781D>N | 0.996 | mis | 0.004884 | rs150644129 |
| 17 | 14:73727963 | LNV | G/A | 1 | 1 | PAPLN | p.709G>S | 1.000 | mis | 0.003488 | rs145250622 |
| 17 | 14:75515277 | LNV | T/C | 1 | 1 | MLH3 | p.361D>G | 1.000 | mis | . | rs749840648 |
| 17 | 15:42740560 | LNV | T/C | 1 | 1 | ZFP106 | p.926R>G | 0.999 | mis | 0.002326 | rs142908361 |
| 17 | 15:78882694 | LNV | A/G | 1 | 1 | CHRNA5 | p.321M>V | 1.000 | mis | . | rs74865777 |
| 17 | 16:16256866 | LNV | G/A | 1 | 1 | ABCC6 | p.1164R>* | . | stop | 0.000116 | rs72653744 |
| 17 | 16:50746199 | LNV | G/A | 1 | 1 | NOD2 | p.793V>M | 0.999 | mis | 0.001628 | rs104895444 |
| 17 | 16:89629429 | LNV | G/C | 1 | 1 | RPL13 | p.205Q>H | 0.991 | mis | 0.001867 | rs146062904 |
| 17 | 19:4409184 | LNV | C/T | 1 | 1 | CHAF1A | p.130L>F | 0.999 | mis | 0.002326 | rs141725942 |
| 17 | 19:35832438 | LNV | G/A | 1 | 1 | CD22 | p.479G>E | 1.000 | mis | . | rs753860332 |
| 17 | 19:54802611 | LNV | A/G | 1 | 1 | LILRA3 | p.213L>P | 1.000 | mis | 0.004076 | rs144505065 |
| 17 | 19:54803979 | LNV | C/G | 1 | 1 | LILRA3 | . | . | spl | 0.004326 | rs11574607 |
| 17 | 19:55325146 | LNV | T/G | 1 | 1 | KIR2DL4 | . | 0.999 | mis | 0.001812 | rs200479581 |
| 17 | 21:46086605 | LNV | GCA/G | 1 | 1 | TSPEAR | . | . | fs | . | . |
| 17 | X:130419322 | LNV | C/G | 1 | 1 | IGSF1 | p.166E>D | 0.998 | mis | 0.000892 | rs201255931 |
| 18 | 1:55077278 | LNV | C/T | 0 | 2 | ACOT11 | . | . | spl | 0.000465 | rs141735196 |
| 18 | 1:71530780 | LNV | G/A | 1 | 1 | ZRANB2 | . | 1.000 | mis | 0.001396 | rs148862349 |
| 18 | 1:202092320 | LNV | C/T | 1 | 2 | GPR37L1 | p.77Q>* | . | stop | . | rs756909328 |
| 18 | 1:247615148 | LNV | T/C | 1 | 2 | OR2B11 | p.46N>S | 0.999 | mis | 0.001628 | rs112807572 |
| 18 | 2:1687882 | LNV | G/A | 1 | 1 | PXDN | p.153S>L | 0.999 | mis | . | rs765145239 |
| 18 | 2:48026630 | LNV | C/G | 1 | 2 | MSH6 | p.503S>C | 0.999 | mis | 0.00186 | rs63750897 |
| 18 | 3:38354498 | LNV | C/T | 1 | 1 | SLC22A14 | p.318P>L | 0.998 | mis | 0.0007 | rs116032919 |
| 18 | 3:46414696 | LNV | T/A | 1 | 1 | CCR5 | p.101C>* | . | stop | 0.000931 | rs1800560 |
| 18 | 3:47894287 | LNV | G/A | 1 | 1 | MAP4 | p.1135H>Y | 0.999 | mis | . | . |
| 18 | 3:49719237 | LNV | C/A | 1 | 1 | APEH | . | 1.000 | mis | . | rs549920856 |
| 18 | 4:983538 | LNV | C/T | 1 | 1 | SLC26A1 | . | 0.999 | mis | . | rs572968482 |
| 18 | 4:995638 | LNV | T/G | 1 | 1 | IDUA | p.254V>G | 1.000 | mis | . | rs764882035 |
| 18 | 4:87666225 | LNV | A/G | 1 | 1 | PTPN13 | p.865H>R | 1.000 | mis | 0.005528 | rs61750815 |
| 18 | 4:155226252 | LNV | G/A | 1 | 1 | DCHS2 | . | . | stop | 0.00221 | rs150179829 |
| 18 | 5:140683301 | LNV | G/GT | 1 | 1 | SLC25A2 | . | . | fs | 0.009813 | rs562886845 |
| 18 | 6:129636693 | LNV | AT/A | 1 | 1 | LAMA2 | . | . | fs | 0.000465 | rs751049992 |
| 18 | 6:137018413 | LNV | C/T | 1 | 1 | MAP3K5 | p.307E>K | 0.999 | mis | 0.000116 | rs368038602 |
| 18 | 7:128492713 | LNV | G/A | 1 | 1 | FLNC | p.1938E>K | 1.000 | mis | . | . |
| 18 | 8:42036464 | LNV | C/G | 1 | 1 | PLAT | p.494G>A | 0.999 | mis | 0.000698 | rs61755432 |
| 18 | 8:53030923 | LNV | G/T | 1 | 1 | ST18 | p.945A>E | 1.000 | mis | 0.004654 | rs117471862 |
| 18 | 8:82606468 | LNV | A/G | 1 | 1 | SLC10A5 | p.247L>P | 0.992 | mis | 0.000581 | rs149116678 |
| 18 | 9:134308126 | LNV | G/A | 1 | 1 | PRRC2B | p.80V>I | 0.999 | mis | 0.002959 | rs41302675 |
| 18 | 10:72468473 | LNV | C/T | 0 | 2 | ADAMTS14 | p.270S>L | 0.998 | mis | 0.009186 | rs61749230 |
| 18 | 10:74700957 | LNV | G/A | 0 | 2 | PLA2G12B | p.146R>W | 0.997 | mis | 0.000233 | rs375272873 |
| 18 | 11:1276432 | LNV | G/T | 1 | 1 | MUC5B | p.5276V>L | 0.999 | mis | 0.007093 | rs55893724 |
| 18 | 12:58016602 | LNV | AGCTGCCCA  GGATTCTG/A | 1 | 1 | SLC26A10 | . | . | fs | 0.001575 | rs546541926 |
| 18 | 13:28844967 | LNV | T/C | 1 | 1 | PAN3 | p.641M>T | 0.999 | mis | 0.001512 | rs141264044 |
| 18 | 13:76335139 | LNV | AGATCTACA  GGATT/A | 1 | 2 | LMO7 | . | . | fs | 0.002302 | rs559563276 |
| 18 | 14:56108429 | LNV | AA/CT | 1 | 1 | KTN1 | p.708K>N | 0.998 | mis | 0.001164 | . |
| 18 | 15:49048567 | LNV | A/G | 1 | 1 | CEP152 | p.960W>R | 1.000 | mis | 0.004275 | rs201342438 |
| 18 | 15:90226796 | LNV | G/A | 1 | 1 | PEX11A | p.186R>* | . | stop | . | rs540530166 |
| 18 | 16:28488944 | LNV | G/T | 1 | 1 | CLN3 | p.404H>N | 0.999 | mis | 0.000349 | rs146610181 |
| 18 | 16:31102589 | LNV | G/A | 1 | 1 | VKORC1 | p.120L>L | 0.999 | mis | 0.001628 | rs7200749 |
| 18 | 16:47536996 | LNV | G/A | 1 | 1 | PHKB | p.134D>N | 0.997 | mis | 0.001047 | rs144486825 |
| 18 | 16:88800139 | LNV | C/T | 1 | 2 | PIEZO1 | p.782G>S | 1.000 | mis | . | rs200970763 |
| 18 | 17:4926868 | LNV | C/T | 1 | 1 | KIF1C | p.912R>W | 0.999 | mis | 0.00128 | rs202232792 |
| 18 | 17:71231644 | LNV | T/C | 1 | 1 | C17orf80 | p.8M>T | 0.992 | mis | . | . |
| 18 | 17:73499120 | LNV | C/T | 1 | 2 | CASKIN2 | p.597A>T | 1.000 | mis | . | . |
| 18 | 18:72345527 | LNV | C/T | 1 | 2 | ZNF407 | p.851T>M | 0.995 | mis | 0.000121 | rs377128271 |
| 18 | 19:3770923 | LNV | C/T | 1 | 1 | RAX2 | p.84R>H | 1.000 | mis | . | rs573043030 |
| 18 | 19:5244476 | LNV | C/T | 1 | 1 | PTPRS | p.336G>R | 0.997 | mis | . | rs560188490 |
| 19 | 2:239234520 | LNV | T/A | 1 | 1 | TRAF3IP1 | p.88L>Q | 1.000 | mis | 0.000233 | rs137999499 |
| 19 | 4:69342109 | LNV | CTTAA/C | 1 | 1 | TMPRSS11E | . | . | fs | 0.000242 | rs768475173 |
| 19 | 4:70346510 | LNV | G/A | 1 | 1 | UGT2B4 | p.477R>W | 1.000 | mis | . | rs778378649 |
| 19 | 6:108843569 | LNV | C/T | 1 | 1 | LACE1 | p.463R>* | . | stop | 0 | rs374172411 |
| 19 | 7:150554592 | LNV | G/C | 1 | 1 | ABP1 | p.345R>P | 0.999 | mis | . | rs766806992 |
| 19 | 8:2965294 | LNV | G/C | 1 | 1 | CSMD1 | p.2261P>A | 1.000 | mis | 0.00527 | rs190894161 |
| 19 | 9:114873997 | LNV | C/T | 1 | 1 | SUSD1 | p.370V>M | 0.999 | mis | 0.003372 | rs146611081 |
| 19 | 12:40734194 | LNV | C/T | 1 | 1 | LRRK2 | p.2016A>V | 0.994 | mis | . | . |
| 19 | 12:50043083 | LNV | G/A | 1 | 1 | FMNL3 | p.782R>W | 1.000 | mis | . | rs756053023 |
| 19 | 14:75330164 | LNV | T/C | 1 | 1 | PROX2 | p.125N>S | 0.999 | mis | . | . |
| 19 | 14:92258883 | LNV | G/A | 1 | 1 | TC2N | p.292T>M | 1.000 | mis | . | rs756200562 |
| 19 | 15:40707653 | LNV | C/T | 1 | 1 | IVD | p.284A>V | 0.999 | mis | 0.000698 | rs28940889 |
| 19 | 15:42379598 | LNV | G/C | 1 | 1 | PLA2G4D | p.52S>W | 1.000 | mis | 0.000465 | rs74977904 |
| 19 | 15:43022889 | LNV | G/A | 1 | 1 | CDAN1 | p.694P>L | 1.000 | mis | . | rs779881606 |
| 19 | 16:74338238 | LNV | C/A | 1 | 1 | PSMD7 | p.159T>N | 0.999 | mis | 0.000116 | rs139257842 |
| 19 | 18:56892836 | LNV | G/T | 1 | 1 | GRP | p.84L>F | 1.000 | mis | . | . |
| 74 | 1:43672605 | LNV | T/G | 1 | 1 | WDR65 | . | . | spl | 0.000116 | rs146369173 |
| 74 | 1:47399894 | LNV | G/A | 1 | 1 | CYP4A11 | p.348R>W | 1.000 | mis | 0.000465 | rs148022251 |
| 74 | 1:108185260 | LNV | G/A | 1 | 1 | VAV3 | p.72A>V | 0.999 | mis | 0.000116 | rs146124612 |
| 74 | 2:44055163 | LNV | C/T | 1 | 1 | ABCG5 | p.198R>Q | 1.000 | mis | 0.001628 | rs141828689 |
| 74 | 2:47639634 | LNV | C/T | 0 | 2 | MSH2 | p.243R>W | 1.000 | mis | 0 | rs138857091 |
| 74 | 2:47709924 | LNV | G/A | 0 | 2 | MSH2 | p.881E>K | 0.998 | mis | . | . |
| 74 | 2:71187158 | LNV | C/T | 1 | 2 | ATP6V1B1 | p.179R>C | 1.000 | mis | . | . |
| 74 | 2:113820124 | LNV | C/T | 1 | 2 | IL36RN | p.113S>L | 0.999 | mis | 0.00314 | rs144478519 |
| 74 | 2:228175508 | LNV | C/T | 0 | 2 | COL4A3 | p.1591S>F | 0.996 | mis | . | rs558813497 |
| 74 | 3:12857896 | LNV | C/T | 1 | 1 | CAND2 | p.489R>C | 0.999 | mis | 0.003064 | rs200149446 |
| 74 | 5:38923273 | LNV | G/A | 1 | 1 | OSMR | p.596R>Q | 0.999 | mis | 0.000349 | rs142163960 |
| 74 | 5:41934110 | LNV | T/C | 1 | 1 | FBXO4 | p.237Y>H | 1.000 | mis | 0.003837 | rs141675593 |
| 74 | 6:33247377 | LNV | G/A | 1 | 1 | WDR46 | p.490P>L | 1.000 | mis | 0.000116 | rs372414839 |
| 74 | 6:80720614 | LNV | C/T | 1 | 1 | TTK | p.185R>W | 0.999 | mis | 0.001628 | rs142356650 |
| 74 | 7:134743936 | LNV | AG/A | 1 | 2 | AGBL3 | . | . | fs | . | rs779491777 |
| 74 | 7:150706295 | LNV | G/A | 1 | 2 | NOS3 | p.755R>K | 0.999 | mis | . | rs762779648 |
| 74 | 8:19263328 | LNV | C/G | 1 | 2 | CSGALNACT1 | p.521R>P | 1.000 | mis | 0.005698 | rs61910741 |
| 74 | 8:22972271 | LNV | GT/G | 1 | 1 | TNFRSF10C | . | . | fs | 0.005211 | rs537005361 |
| 74 | 9:137801759 | LNV | T/C | 1 | 1 | FCN1 | p.289N>S | 1.000 | mis | 0.001628 | rs138055828 |
| 74 | 9:140357186 | LNV | C/T | 1 | 1 | PNPLA7 | p.1148G>E | 1.000 | mis | 0.001047 | rs141473646 |
| 74 | 10:44871479 | LNV | C/T | 0 | 2 | CXCL12 | . | 0.999 | mis | 0.000116 | rs200785547 |
| 74 | 10:75029417 | LNV | G/C | 1 | 1 | TTC18 | p.1064N>K | 0.998 | mis | . | . |
| 74 | 11:47008768 | LNV | A/G | 1 | 1 | C11orf49 | p.19H>R | 0.998 | mis | 0.004303 | rs150919488 |
| 74 | 11:64056551 | LNV | C/T | 1 | 1 | GPR137 | . | 0.999 | mis | . | rs201737201 |
| 74 | 12:27523100 | LNV | C/T | 1 | 2 | ARNTL2 | p.74S>F | 0.999 | mis | 0.001628 | rs71541524 |
| 74 | 12:55368313 | LNV | C/T | 1 | 2 | TESPA1 | p.12E>K | 0.996 | mis | . | rs200900573 |
| 74 | 12:57389007 | LNV | C/T | 1 | 2 | GPR182 | p.5P>L | 0.990 | mis | . | . |
| 74 | 12:117448195 | LNV | CGT/C | 1 | 2 | FBXW8 | . | . | fs | 0.00424 | rs371138207 |
| 74 | 14:24675755 | LNV | A/G | 1 | 2 | TSSK4 | p.89Y>C | 1.000 | mis | 0.003488 | rs34083933 |
| 74 | 15:45713359 | LNV | A/G | 1 | 1 | SPATA5L1 | p.738Y>C | 1.000 | mis | 0.000931 | rs144102603 |
| 74 | 16:30566693 | LNV | C/T | 1 | 2 | ZNF764 | p.349R>H | 1.000 | mis | 0.003295 | rs201099236 |
| 74 | 16:88782483 | LNV | C/T | 1 | 1 | PIEZO1 | p.2392E>K | 0.999 | mis | . | rs528448732 |
| 74 | 17:3660373 | LNV | G/C | 1 | 2 | ITGAE | p.359S>* | . | stop | . | rs767193179 |
| 74 | 17:8135414 | LNV | C/T | 1 | 1 | CTC1 | p.731R>Q | 0.999 | mis | 0.000728 | rs201891953 |
| 74 | 17:17075132 | LNV | A/G | 1 | 1 | MPRIP | p.755E>G | 1.000 | mis | . | . |
| 74 | 17:26943984 | LNV | C/T | 1 | 1 | KIAA0100 | p.1982G>R | 0.999 | mis | . | . |
| 74 | 17:39768783 | LNV | GAGAC/G | 1 | 1 | KRT16 | . | . | fs | . | rs764850876 |
| 74 | 17:42336674 | LNV | C/T | 1 | 1 | SLC4A1 | p.245V>M | 1.000 | mis | 0.000698 | rs148170067 |
| 74 | 19:8140008 | LNV | C/T | 1 | 1 | FBN3 | p.2550R>H | 0.999 | mis | 0.000349 | rs142975316 |
| 74 | 20:33588598 | LNV | A/C | 1 | 1 | MYH7B | p.1778K>Q | 1.000 | mis | 0.000596 | rs201800075 |
| 74 | 20:37255691 | LNV | G/C | 1 | 1 | ARHGAP40 | p.130G>R | 0.999 | mis | 0.002828 | rs200985539 |
| 74 | 21:47635168 | LNV | G/A | 1 | 2 | LSS | p.313Q>* | . | stop | . | rs776090400 |
| 84 | 1:16915344 | LNV | C/A | 1 | 1 | NBPF1 | . | . | stop | . | rs570287171 |
| 84 | 1:144866600 | LNV | C/T | 1 | 1 | PDE4DIP | p.1775R>H | 0.999 | mis | 0.000931 | rs144059949 |
| 84 | 1:153921085 | LNV | G/C | 1 | 1 | CRTC2 | p.570S>R | 1.000 | mis | 0.000116 | rs201098465 |
| 84 | 1:156617399 | LNV | C/T | 1 | 1 | BCAN | p.189P>L | 1.000 | mis | . | rs780124787 |
| 84 | 2:220404974 | LNV | G/C | 1 | 1 | CHPF | p.325L>V | 0.995 | mis | 0.000814 | rs200369127 |
| 84 | 3:130114278 | LNV | C/T | 1 | 1 | COL6A5 | p.1180R>C | 0.998 | mis | . | rs543746446 |
| 84 | 3:130150407 | LNV | T/G | 1 | 1 | COL6A5 | p.1783S>A | 0.999 | mis | . | rs545379693 |
| 84 | 3:167254681 | LNV | A/G | 1 | 1 | WDR49 | p.292L>S | 0.999 | mis | 0.003837 | rs150216870 |
| 84 | 4:6596373 | LNV | C/T | 1 | 1 | MAN2B2 | p.324T>M | 1.000 | mis | . | rs148186055 |
| 84 | 4:71063801 | LNV | A/G | 1 | 1 | ODAM | p.101Q>R | 0.999 | mis | 0.001332 | rs201514554 |
| 84 | 4:75091105 | LNV | G/C | 1 | 1 | MTHFD2L | p.267A>P | 1.000 | mis | . | . |
| 84 | 4:108552883 | LNV | G/A | 1 | 1 | PAPSS1 | p.547T>M | 1.000 | mis | 0.001279 | rs145511221 |
| 84 | 5:140531409 | LNV | C/A | 1 | 1 | PCDHB6 | p.524S>Y | 0.999 | mis | . | rs782177492 |
| 84 | 6:36887436 | LNV | A/G | 1 | 1 | C6orf89 | p.310H>R | 1.000 | mis | 0.002442 | rs144031629 |
| 84 | 6:44241130 | LNV | C/A | 1 | 1 | TMEM151B | p.155R>S | 0.995 | mis | . | . |
| 84 | 6:109774985 | LNV | C/T | 1 | 1 | MICAL1 | p.108A>T | 1.000 | mis | 0.00093 | rs150984265 |
| 84 | 8:72981374 | LNV | G/A | 1 | 1 | TRPA1 | p.110Q>* | . | stop | . | . |
| 84 | 9:84608198 | LNV | C/A | 1 | 1 | FAM75D1 | p.938S>Y | 0.995 | mis | 0.005354 | rs145142439 |
| 84 | 9:95776262 | LNV | G/T | 1 | 1 | FGD3 | p.388Q>H | 1.000 | mis | 0.000482 | rs202214207 |
| 84 | 9:128678097 | LNV | C/T | 1 | 1 | PBX3 | p.61A>V | 1.000 | mis | 0.00816 | rs145687528 |
| 84 | 9:130160281 | LNV | C/A | 1 | 1 | SLC2A8 | p.106P>H | 1.000 | mis | 0.00047 | rs368823866 |
| 84 | 9:131418941 | LNV | G/A | 1 | 1 | WDR34 | p.22A>V | 0.999 | mis | 0.005504 | rs201715229 |
| 84 | 10:120931928 | LNV | C/A | 1 | 1 | PRDX3 | p.173G>C | 1.000 | mis | 0.009419 | rs11554923 |
| 84 | 10:135209255 | LNV | T/G | 1 | 1 | MTG1 | p.51C>G | 1.000 | mis | 0.003953 | rs141260910 |
| 84 | 11:118849721 | LNV | C/G | 1 | 1 | FOXR1 | p.64P>R | 1.000 | mis | . | rs782103158 |
| 84 | 12:109186501 | LNV | T/A | 1 | 1 | SSH1 | p.485D>V | 0.999 | mis | 0.002674 | rs147936147 |
| 84 | 12:118533464 | LNV | G/A | 1 | 1 | VSIG10 | p.79R>C | 1.000 | mis | 0.002699 | rs142979297 |
| 84 | 12:120214570 | LNV | C/T | 1 | 1 | CIT | p.581R>Q | 1.000 | mis | 0.000349 | rs201303983 |
| 84 | 13:103394544 | LNV | G/A | 1 | 1 | CCDC168 | p.2835Q>* | . | stop | 0.000629 | rs201710661 |
| 84 | 14:45644816 | LNV | A/C | 1 | 1 | FANCM | p.953K>N | 0.992 | mis | 0.002331 | rs142864437 |
| 84 | 19:2210489 | LNV | G/T | 1 | 1 | DOT1L | p.366G>C | 0.999 | mis | . | . |
| 84 | 19:36277614 | LNV | A/G | 1 | 1 | ARHGAP33 | p.612S>G | 0.999 | mis | . | rs568719142 |
| 84 | 19:38985219 | LNV | G/C | 1 | 1 | RYR1 | p.2168V>L | 1.000 | mis | . | rs118192176 |
| 84 | 19:39398201 | LNV | C/T | 1 | 1 | NFKBIB | p.205R>C | 0.999 | mis | 0.000818 | rs187346322 |
| 84 | 19:56001772 | LNV | C/T | 1 | 1 | SSC5D | . | . | stop | . | . |
| 84 | 21:34003392 | LNV | CAG/C | 1 | 1 | SYNJ1 | . | . | fs | . | . |
| 84 | 21:44480591 | LNV | G/A | 1 | 1 | CBS | p.369R>C | 1.000 | mis | 0.003953 | rs117687681 |
| 84 | 22:30775793 | LNV | C/A | 1 | 1 | RNF215 | p.340V>L | 1.000 | mis | 0.000466 | rs143483218 |
| 84 | 22:42967207 | LNV | CAAGT/C | 1 | 1 | SERHL2 | . | . | fs | 0.002181 | rs566977033 |
| 84 | 22:50654298 | LNV | T/G | 1 | 1 | SELO | . | . | spl | . | rs200107024 |
| 84 | 22:51220776 | LNV | C/T | 1 | 1 | RABL2B | . | . | spl | . | . |
| 113 | 1:16464416 | LNV | C/T | 1 | 2 | EPHA2 | p.415R>H | 0.999 | mis | . | . |
| 113 | 1:144931218 | LNV | G/C | 1 | 1 | PDE4DIP | p.164P>R | 1.000 | mis | 0.003372 | rs144628175 |
| 113 | 1:156846300 | LNV | G/A | 1 | 1 | NTRK1 | p.545E>K | 1.000 | mis | 0.000698 | rs150579345 |
| 113 | 1:178435202 | LNV | C/T | 1 | 1 | RASAL2 | p.1038R>W | 1.000 | mis | . | rs776658051 |
| 113 | 2:165551734 | LNV | C/T | 1 | 1 | COBLL1 | p.761R>Q | 1.000 | mis | . | . |
| 113 | 3:101080620 | LNV | T/C | 1 | 1 | SENP7 | p.456D>G | 0.999 | mis | . | . |
| 113 | 5:79054599 | LNV | C/T | 0 | 1 | CMYA5 | p.3712P>S | 1.000 | mis | 0.002554 | rs61744879 |
| 113 | 6:31105900 | LNV | C/A | 1 | 1 | PSORS1C1 | . | 1.000 | mis | 0.000374 | rs139472873 |
| 113 | 6:47252045 | LNV | G/A | 1 | 1 | TNFRSF21 | p.291T>I | 0.999 | mis | 0.001395 | rs146578369 |
| 113 | 7:48547481 | LNV | C/T | 1 | 2 | ABCA13 | p.4454R>C | 1.000 | mis | 0.007871 | rs76060602 |
| 113 | 10:112559642 | LNV | G/A | 1 | 1 | RBM20 | p.589R>Q | 0.999 | mis | 0.000314 | rs368716639 |
| 113 | 11:74053959 | LNV | C/G | 1 | 2 | PGM2L1 | p.471L>F | 0.999 | mis | . | rs769563330 |
| 113 | 11:74547656 | LNV | C/T | 0 | 2 | RNF169 | p.670R>* | . | stop | . | rs748210539 |
| 113 | 11:95519380 | LNV | C/T | 0 | 2 | FAM76B | p.89R>H | 1.000 | mis | 0.002331 | rs200608990 |
| 113 | 12:27641445 | LNV | C/T | 0 | 2 | C12orf70 | p.189R>W | 0.999 | mis | 0.002828 | rs148846851 |
| 113 | 12:39997728 | LNV | TG/T | 1 | 2 | ABCD2 | . | . | fs | . | . |
| 113 | 14:62541943 | LNV | A/G | 1 | 1 | SYT16 | p.276E>G | 0.995 | mis | 0.000358 | rs200718503 |
| 113 | 16:16215877 | LNV | G/A | 1 | 1 | ABCC1 | p.1146V>I | 0.999 | mis | 0.000117 | rs28706727 |
| 113 | 17:39643658 | LNV | TC/AA | 0 | 1 | KRT36 | p.311E>L | . | mis | 0.000581 | . |
| 113 | 19:1057343 | LNV | G/A | 1 | 1 | ABCA7 | p.1599V>M | 1.000 | mis | 0.003605 | rs117187003 |
| 113 | 21:45815425 | LNV | C/G | 1 | 2 | TRPM2 | p.641I>M | 0.995 | mis | 0.002326 | rs150484698 |
| 113 | 21:45845699 | LNV | G/A | 1 | 2 | TRPM2 | p.1262V>M | 1.000 | mis | 0.002327 | rs150071509 |
| 113 | X:9716621 | LNV | C/T | 1 | 2 | GPR143 | p.181V>M | 0.999 | mis | 0.000149 | rs200870584 |
| 121 | 1:62733957 | LNV | AC/A | 1 | 1 | KANK4 | . | . | spl | 0.008117 | rs568600301 |
| 121 | 1:74621445 | LNV | G/A | 1 | 1 | LRRIQ3 | p.227R>C | 1.000 | mis | 0.00087 | rs199536965 |
| 121 | 1:150974740 | LNV | C/T | 1 | 1 | FAM63A | . | . | stop | 0.000698 | rs142215084 |
| 121 | 2:28117455 | LNV | C/G | 1 | 1 | BRE | p.11S>C | 0.999 | mis | 0.004302 | rs144572761 |
| 121 | 2:135744418 | LNV | C/T | 1 | 1 | YSK4 | . | 1.000 | mis | 0.001628 | rs141304858 |
| 121 | 2:171055731 | LNV | GGATT/G | 1 | 1 | MYO3B | . | . | fs | 0.000128 | rs747841852 |
| 121 | 3:52848037 | LNV | C/T | 1 | 2 | ITIH4 | p.863G>S | 0.999 | mis | 0.005581 | rs151083454 |
| 121 | 3:130293046 | LNV | G/C | 1 | 1 | COL6A6 | p.1075G>A | 0.990 | mis | 0.004082 | rs149093613 |
| 121 | 4:1374746 | LNV | C/T | 1 | 1 | UVSSA | p.611R>W | 1.000 | mis | 0.000582 | rs140176848 |
| 121 | 4:3430361 | LNV | G/A | 1 | 1 | RGS12 | p.1163R>Q | 0.997 | mis | . | rs774321500 |
| 121 | 4:169060675 | LNV | C/T | 1 | 1 | ANXA10 | p.47R>C | 0.997 | mis | 0.006279 | rs111362741 |
| 121 | 5:63991426 | LNV | C/T | 0 | 2 | FAM159B | p.95T>M | 0.990 | mis | 0.000943 | rs201615430 |
| 121 | 5:110454719 | LNV | A/G | 1 | 1 | WDR36 | p.658D>G | 1.000 | mis | 0.007211 | rs34595252 |
| 121 | 5:174948943 | LNV | C/T | 1 | 1 | SFXN1 | p.266P>S | 0.995 | mis | 0.005814 | rs34907038 |
| 121 | 6:10796256 | LNV | G/A | 1 | 1 | MAK | p.373P>L | 0.993 | mis | . | rs771764863 |
| 121 | 6:36887436 | LNV | A/G | 1 | 1 | C6orf89 | p.310H>R | 1.000 | mis | 0.002442 | rs144031629 |
| 121 | 6:46657127 | LNV | T/C | 1 | 1 | TDRD6 | p.421V>A | 1.000 | mis | 0.000233 | rs375684816 |
| 121 | 7:2578341 | LNV | G/A | 0 | 2 | BRAT1 | p.610R>W | 1.000 | mis | 0.001661 | rs61753094 |
| 121 | 7:150935000 | LNV | C/T | 1 | 2 | CHPF2 | p.518R>* | . | stop | 0.000116 | rs201697000 |
| 121 | 8:71050492 | LNV | G/A | 1 | 2 | NCOA2 | p.1035P>L | 1.000 | mis | 0.000244 | rs369246166 |
| 121 | 8:82373736 | LNV | G/A | 1 | 2 | FABP9 | p.8T>I | 0.999 | mis | . | rs747760713 |
| 121 | 8:134472099 | LNV | G/A | 1 | 1 | ST3GAL1 | p.311R>C | 1.000 | mis | . | rs757635387 |
| 121 | 8:145639654 | LNV | T/C | 1 | 2 | SLC39A4 | p.356T>A | 0.999 | mis | 0.00547 | rs75920625 |
| 121 | 8:145639681 | LNV | G/C | 1 | 2 | SLC39A4 | p.347L>V | 1.000 | mis | 0.006167 | rs1871534 |
| 121 | 9:4663124 | LNV | G/C | 1 | 2 | PPAPDC2 | . | 1.000 | mis | 0.001744 | rs41279553 |
| 121 | 9:121930001 | LNV | G/C | 1 | 1 | DBC1 | p.549C>W | 1.000 | mis | . | rs758518892 |
| 121 | 11:1254287 | LNV | G/A | 1 | 2 | MUC5B | p.704V>M | 0.999 | mis | 0.000708 | rs200294410 |
| 121 | 11:1258377 | LNV | G/A | 1 | 2 | MUC5B | p.1094A>T | 1.000 | mis | 0.000726 | rs199977970 |
| 121 | 11:65061679 | LNV | G/C | 1 | 2 | POLA2 | p.434V>L | 0.993 | mis | 0.000582 | rs142790860 |
| 121 | 11:119045723 | LNV | C/T | 0 | 2 | NLRX1 | p.471R>W | 0.992 | mis | 0.000349 | rs145673513 |
| 121 | 11:123887107 | LNV | A/G | 0 | 2 | OR10G4 | p.276T>A | 0.996 | mis | 0.001047 | rs112431396 |
| 121 | 11:125503234 | LNV | A/G | 0 | 2 | CHEK1 | p.201M>V | 0.999 | mis | . | rs771316687 |
| 121 | 12:55250658 | LNV | G/T | 1 | 1 | MUCL1 | p.69A>S | 0.999 | mis | . | rs143525110 |
| 121 | 12:96407586 | LNV | T/C | 1 | 1 | LTA4H | p.417Y>C | 0.998 | mis | . | . |
| 121 | 13:37576626 | LNV | AAG/A | 1 | 1 | EXOSC8 | . | . | fs | . | rs749835724 |
| 121 | 13:113771856 | LNV | G/A | 1 | 1 | F7 | p.251A>T | 1.000 | mis | . | . |
| 121 | 14:71197102 | LNV | A/G | 1 | 1 | MAP3K9 | p.1118S>P | 1.000 | mis | . | rs757301027 |
| 121 | 14:102675693 | LNV | A/G | 1 | 1 | WDR20 | . | 1.000 | mis | . | . |
| 121 | 15:62359813 | LNV | A/G | 1 | 2 | C2CD4A | p.1M>V | . | start | 0.000116 | rs372637667 |
| 121 | 15:78841207 | LNV | T/G | 1 | 1 | PSMA4 | p.236L>W | 1.000 | mis | 0.000233 | rs147521961 |
| 121 | 16:815549 | LNV | T/C | 1 | 1 | MSLN | p.243S>P | 1.000 | mis | 0.003733 | rs75279195 |
| 121 | 16:815565 | LNV | T/C | 1 | 1 | MSLN | p.248L>P | 1.000 | mis | 0.003736 | rs77260498 |
| 121 | 16:2816519 | LNV | G/A | 1 | 1 | SRRM2 | p.1997R>H | 0.996 | mis | 0.005814 | rs138447860 |
| 121 | 16:3599162 | LNV | G/C | 1 | 1 | NLRC3 | p.861N>K | 1.000 | mis | . | rs199475943 |
| 121 | 16:16244086 | LNV | C/T | 1 | 2 | ABCC6 | p.1472M>I | 0.999 | mis | . | . |
| 121 | 16:19020738 | LNV | G/T | 1 | 2 | TMC7 | . | . | spl | 0.000581 | rs145110118 |
| 121 | 16:75269697 | LNV | G/A | 1 | 2 | BCAR1 | p.413P>L | 0.999 | mis | 0.005474 | rs200836207 |
| 121 | 16:85682290 | LNV | C/A | 1 | 2 | KIAA0182 | p.16T>N | 1.000 | mis | . | rs754199513 |
| 121 | 16:88798310 | LNV | G/T | 1 | 1 | PIEZO1 | p.1000F>L | 1.000 | mis | . | rs568280615 |
| 121 | 16:89620204 | LNV | G/T | 1 | 1 | SPG7 | . | 1.000 | mis | 0.000465 | rs199689138 |
| 121 | 17:3716416 | LNV | C/G | 1 | 1 | C17orf85 | p.595K>N | 1.000 | mis | 0.001047 | rs138378952 |
| 121 | 17:8157385 | LNV | G/A | 1 | 1 | PFAS | p.44V>M | 1.000 | mis | . | rs748475399 |
| 121 | 17:11459147 | LNV | C/T | 1 | 1 | SHISA6 | p.297P>L | 0.999 | mis | 0.0044 | rs185956842 |
| 121 | 17:36492976 | LNV | G/A | 1 | 1 | GPR179 | p.371P>L | 1.000 | mis | 0.000118 | rs371812444 |
| 121 | 17:74462267 | LNV | T/C | 1 | 1 | AANAT | p.5S>P | 0.999 | mis | . | . |
| 121 | 18:10757983 | LNV | C/G | 1 | 1 | PIEZO2 | p.1278D>H | 1.000 | mis | 0.000314 | rs371703803 |
| 121 | 18:43796123 | LNV | T/C | 1 | 1 | C18orf25 | p.93C>R | 1.000 | mis | 0.004318 | rs201155841 |
| 121 | 19:40993655 | LNV | A/G | 1 | 1 | SPTBN4 | p.74H>R | 0.999 | mis | . | . |
| 121 | 20:18507167 | LNV | G/C | 1 | 1 | SEC23B | p.329A>P | 1.000 | mis | 0.000349 | rs143417821 |
| 121 | 20:19981477 | LNV | G/A | 1 | 1 | RIN2 | p.911S>N | 0.996 | mis | . | rs772364038 |
| 121 | 20:23433446 | LNV | C/T | 1 | 1 | CST11 | p.1M>I | . | start | . | . |
| 121 | 20:25656688 | LNV | G/T | 1 | 1 | ZNF337 | p.412S>R | 0.999 | mis | . | rs557218253 |
| 121 | 20:30785367 | LNV | G/C | 1 | 1 | PLAGL2 | p.127L>V | 0.999 | mis | . | rs774355442 |
| 121 | 20:31021464 | LNV | C/T | 1 | 1 | ASXL1 | p.488S>F | 0.998 | mis | . | rs781545960 |
| 121 | 20:33442648 | LNV | T/C | 1 | 1 | GGT7 | p.394N>S | 0.999 | mis | 0.001395 | rs141899095 |
| 121 | 20:34065726 | LNV | A/G | 1 | 1 | CEP250 | p.632R>G | 0.999 | mis | . | rs754556013 |
| 121 | 22:24236972 | LNV | A/C | 1 | 1 | MIF | p.41H>P | 1.000 | mis | 0.000248 | rs200005486 |
| 121 | 22:24717334 | LNV | G/C | 1 | 1 | SPECC1L | p.129R>P | 0.990 | mis | . | rs199596465 |
| 121 | 22:29885935 | LNV | C/T | 1 | 1 | NEFH | p.769P>L | 0.999 | mis | . | . |
| 121 | 22:30421668 | LNV | G/A | 1 | 1 | MTMR3 | p.1159V>I | 0.994 | mis | . | . |
| 129 | 2:174128561 | LNV | A/T | 1 | 1 | ZAK | p.547Q>L | 0.999 | mis | 0.001935 | rs188158548 |
| 129 | 3:57107810 | LNV | G/A | 1 | 1 | SPATA12 | . | 0.999 | mis | . | rs762158806 |
| 129 | 4:79343124 | LNV | C/T | 1 | 1 | FRAS1 | p.1550L>F | 1.000 | mis | 0.00419 | rs148663672 |
| 129 | 4:187509931 | LNV | C/T | 1 | 1 | FAT1 | p.4528E>K | 0.999 | mis | 0.007942 | rs192609167 |
| 129 | 5:1243683 | LNV | C/T | 1 | 1 | SLC6A18 | p.382P>L | 0.999 | mis | 0.000121 | rs199865996 |
| 129 | 6:31612835 | LNV | C/G | 1 | 1 | BAG6 | p.419E>D | 0.999 | mis | . | . |
| 129 | 6:35426147 | LNV | T/C | 1 | 1 | FANCE | p.348L>P | 1.000 | mis | . | . |
| 129 | 6:117050784 | LNV | T/A | 1 | 1 | KPNA5 | p.438S>T | 0.999 | mis | . | . |
| 129 | 7:5417170 | LNV | G/A | 1 | 1 | TNRC18 | p.765P>S | 0.999 | mis | 0.007265 | rs200992316 |
| 129 | 7:34125622 | LNV | C/T | 1 | 1 | BMPER | p.555R>W | 0.992 | mis | 0.009535 | rs10249320 |
| 129 | 7:112424213 | LNV | G/A | 1 | 1 | TMEM168 | p.223P>L | 1.000 | mis | . | rs759125359 |
| 129 | 7:127999537 | LNV | C/A | 1 | 1 | PRRT4 | p.170R>M | 0.999 | mis | . | . |
| 129 | 10:101514289 | LNV | G/A | 1 | 1 | CUTC | . | 1.000 | mis | . | . |
| 129 | 11:19955270 | LNV | C/T | 1 | 1 | NAV2 | p.430R>W | 0.994 | mis | . | rs754191884 |
| 129 | 12:22623772 | LNV | T/C | 1 | 1 | KIAA0528 | p.811K>R | 1.000 | mis | 0.001744 | rs138291438 |
| 129 | 14:24676570 | LNV | C/CT | 1 | 1 | TSSK4 | . | . | fs | 0.000121 | . |
| 129 | 14:76525674 | LNV | C/T | 1 | 1 | IFT43 | . | 1.000 | mis | . | rs547721035 |
| 129 | 16:2367764 | LNV | T/A | 1 | 1 | ABCA3 | . | 1.000 | mis | 0.004535 | rs149989682 |
| 129 | 17:10543680 | LNV | C/T | 1 | 1 | MYH3 | p.799R>H | 1.000 | mis | . | rs745696071 |
| 129 | 17:39623520 | LNV | G/A | 1 | 1 | KRT32 | p.20R>W | 0.999 | mis | 0.000116 | rs144589679 |
| 131 | 3:50597135 | LNV | G/T | 0 | 1 | C3orf18 | p.148P>T | 1.000 | mis | . | . |
| 131 | 3:196388568 | LNV | G/A | 0 | 1 | LRRC33 | p.685R>H | 0.998 | mis | 0 | rs140011218 |
| 131 | 5:110434448 | LNV | C/T | 1 | 1 | WDR36 | p.163A>V | 0.999 | mis | 0.0057 | rs62376783 |
| 131 | 5:112177739 | LNV | C/G | 1 | 1 | APC | p.2150L>V | 0.991 | mis | . | . |
| 131 | 5:115822497 | LNV | G/A | 1 | 1 | SEMA6A | p.304R>C | 0.999 | mis | 0.000119 | rs369698820 |
| 131 | 6:152129063 | LNV | C/T | 0 | 1 | ESR1 | p.6H>Y | 0.995 | mis | 0.005116 | rs139960913 |
| 131 | 9:97365799 | LNV | C/A | 0 | 1 | FBP1 | p.294G>V | 1.000 | mis | . | rs780528686 |
| 131 | 9:100232952 | LNV | G/A | 0 | 1 | TDRD7 | . | . | spl | . | . |
| 131 | 9:140218293 | LNV | C/T | 0 | 1 | EXD3 | p.690G>R | 0.999 | mis | 0.00012 | rs374698744 |
| 131 | 12:56030812 | LNV | T/C | 0 | 1 | OR10P1 | p.46I>T | 0.999 | mis | 0.000233 | rs200645789 |
| 131 | 12:122710554 | LNV | G/C | 1 | 1 | DIABLO | p.3A>G | 0.999 | mis | 0.000582 | rs202028496 |
| 131 | 13:52952024 | LNV | C/A | 0 | 1 | THSD1 | p.694R>I | 0.999 | mis | 0.002674 | rs73484102 |
| 131 | 13:52952634 | LNV | C/G | 0 | 1 | THSD1 | p.491D>H | 0.993 | mis | 0.002791 | rs56013270 |
| 131 | 13:53216701 | LNV | CA/C | 0 | 1 | HNRNPA1L2 | . | . | fs | 0.006545 | rs374312718 |
| 131 | 13:76381617 | LNV | C/A | 0 | 1 | LMO7 | . | 0.999 | mis | 0.00014 | rs368045003 |
| 131 | 13:103338721 | LNV | T/C | 0 | 1 | METTL21C | p.152N>S | 0.999 | mis | 0.002907 | rs138041652 |
| 131 | 22:51018173 | LNV | C/G | 0 | 1 | CHKB | p.338L>F | 0.994 | mis | . | . |
| 138 | 1:18023808 | LNV | G/A | 0 | 2 | ARHGEF10L | p.1219G>E | 1.000 | mis | . | rs747231907 |
| 138 | 1:161163480 | LNV | C/T | 1 | 1 | ADAMTS4 | p.562R>H | 0.996 | mis | . | rs753448015 |
| 138 | 1:227261566 | LNV | A/T | 0 | 2 | CDC42BPA | . | 1.000 | mis | . | rs199653014 |
| 138 | 2:163167419 | LNV | T/C | 1 | 1 | IFIH1 | p.160N>D | 0.998 | mis | 0.000931 | rs74162075 |
| 138 | 2:177033955 | LNV | C/T | 1 | 1 | HOXD3 | p.38T>M | 0.999 | mis | 0 | rs75250034 |
| 138 | 2:179424880 | LNV | A/G | 1 | 1 | TTN | p.27019I>T | 0.999 | mis | . | rs397517733 |
| 138 | 2:179440876 | LNV | G/A | 1 | 1 | TTN | p.21687A>V | 1.000 | mis | . | . |
| 138 | 2:186661666 | LNV | G/C | 1 | 1 | FSIP2 | p.3357S>T | 0.990 | mis | . | . |
| 138 | 2:215279132 | LNV | C/A | 1 | 2 | VWC2L | p.72S>Y | 0.999 | mis | . | . |
| 138 | 3:38739931 | LNV | G/A | 1 | 2 | SCN10A | p.1594R>C | 1.000 | mis | 0 | rs373347787 |
| 138 | 3:48451889 | LNV | C/T | 1 | 2 | PLXNB1 | p.1832R>H | 1.000 | mis | 0.001047 | rs140052429 |
| 138 | 3:49698316 | LNV | C/T | 1 | 2 | BSN | p.3013S>L | 1.000 | mis | 0.001628 | rs140696444 |
| 138 | 3:179483498 | LNV | A/G | 1 | 2 | USP13 | p.759R>G | 0.997 | mis | 0.000116 | rs199954201 |
| 138 | 4:87643478 | LNV | G/C | 1 | 1 | PTPN13 | p.500R>P | 0.998 | mis | 0.003265 | rs61757789 |
| 138 | 4:119948029 | LNV | C/G | 1 | 2 | SYNPO2 | p.169Q>E | 0.999 | mis | . | rs780728054 |
| 138 | 4:187629727 | LNV | G/C | 1 | 2 | FAT1 | p.419L>V | 0.996 | mis | 0.000122 | rs375942121 |
| 138 | 5:16481181 | LNV | C/T | 1 | 1 | FAM134B | p.203V>M | 0.993 | mis | 0.004375 | rs143878016 |
| 138 | 6:153043077 | LNV | C/T | 1 | 1 | MYCT1 | p.133R>C | 0.990 | mis | 0.002558 | rs41292880 |
| 138 | 8:17867236 | LNV | C/A | 0 | 2 | PCM1 | p.1715Q>K | 1.000 | mis | . | rs755922663 |
| 138 | 8:30705918 | LNV | A/T | 0 | 2 | TEX15 | p.206L>I | 0.999 | mis | 0.000233 | rs368353494 |
| 138 | 10:90351202 | LNV | T/G | 1 | 1 | LIPJ | p.26I>S | 1.000 | mis | 0.003954 | rs147165540 |
| 138 | 11:3846253 | LNV | C/T | 1 | 1 | PGAP2 | . | 0.999 | mis | 0.001163 | rs117338939 |
| 138 | 11:64136019 | LNV | G/A | 1 | 1 | RPS6KA4 | p.421R>H | 0.997 | mis | 0.001407 | rs199674030 |
| 138 | 11:117054496 | LNV | G/A | 0 | 2 | SIDT2 | . | 0.991 | mis | 0.003142 | rs149441737 |
| 138 | 12:124416338 | LNV | G/T | 1 | 1 | DNAH10 | p.4240E>* | . | stop | 0.00024 | rs199945399 |
| 138 | 12:129189821 | LNV | C/G | 1 | 1 | TMEM132C | p.770R>G | 0.991 | mis | . | rs776145107 |
| 138 | 16:2098713 | LNV | G/A | 1 | 1 | TSC2 | p.33G>S | 0.999 | mis | . | rs370230541 |
| 138 | 16:15788064 | LNV | C/T | 1 | 1 | NDE1 | p.249A>V | 0.990 | mis | 0.000233 | rs139150117 |
| 138 | 16:28837588 | LNV | C/T | 1 | 1 | ATXN2L | p.165R>W | 1.000 | mis | 0.000349 | rs142953622 |
| 138 | 16:70316576 | LNV | T/C | 1 | 1 | AARS | p.31T>A | 0.994 | mis | . | . |
| 138 | 20:2636036 | LNV | G/A | 1 | 2 | NOP56 | p.212R>H | 0.999 | mis | 0.00093 | rs145743946 |
| 138 | 20:9364916 | LNV | C/T | 1 | 2 | PLCB4 | p.308R>C | 1.000 | mis | 0.000349 | rs78074693 |
| 138 | 20:33575434 | LNV | C/T | 1 | 2 | MYH7B | p.450R>C | 0.999 | mis | 0.006201 | rs45522831 |
| 138 | 22:19496066 | LNV | C/T | 1 | 1 | CDC45 | p.389R>C | 0.992 | mis | . | rs776018185 |
| 138 | X:114426292 | LNV | G/A | 1 | 1 | RBMXL3 | p.763G>D | 1.000 | mis | 0.003764 | rs199838194 |
| 198 | 1:3669131 | LNV | C/G | 1 | 1 | CCDC27 | p.29S>C | 0.999 | mis | . | rs758723696 |
| 198 | 2:131487437 | LNV | G/C | 1 | 1 | GPR148 | p.238C>S | 0.991 | mis | 0.000116 | rs138212672 |
| 198 | 2:220342661 | LNV | C/T | 1 | 1 | SPEG | p.1621R>C | 1.000 | mis | 0.000359 | rs55646900 |
| 198 | 3:52255841 | LNV | C/A | 1 | 1 | TLR9 | p.831G>C | 0.999 | mis | . | . |
| 198 | 3:148714249 | LNV | G/C | 1 | 1 | GYG1 | p.102D>H | 1.000 | mis | 0.00186 | rs143137713 |
| 198 | 3:158520026 | LNV | GCCCTGCC  GGC/G | 1 | 2 | MFSD1 | . | . | fs | . | . |
| 198 | 5:140730832 | LNV | C/G | 1 | 1 | PCDHGB1 | . | 1.000 | mis | . | rs748596533 |
| 198 | 5:140755929 | LNV | C/G | 1 | 1 | PCDHGA6 | . | 0.991 | mis | 0.005465 | rs186469397 |
| 198 | 5:147494001 | LNV | G/A | 1 | 1 | SPINK5 | p.655G>D | 1.000 | mis | 0.003865 | rs142227576 |
| 198 | 7:101713691 | LNV | G/A | 1 | 1 | CUX1 | p.99V>I | 0.992 | mis | 0.005 | rs148322402 |
| 198 | 9:841848 | LNV | G/C | 1 | 1 | DMRT1 | p.4D>H | 0.999 | mis | . | rs764351242 |
| 198 | 9:35870070 | LNV | G/A | 1 | 1 | OR13J1 | p.110T>M | 1.000 | mis | . | rs759818424 |
| 198 | 10:102738916 | LNV | C/T | 1 | 1 | SEMA4G | p.291R>C | 1.000 | mis | . | . |
| 198 | 10:103588870 | LNV | G/A | 1 | 1 | KCNIP2 | p.119R>C | 0.999 | mis | 0.000349 | rs72546683 |
| 198 | 11:6425014 | LNV | C/T | 1 | 1 | APBB1 | p.254D>N | 0.999 | mis | 0.000349 | rs139147263 |
| 198 | 11:49974770 | LNV | G/T | 1 | 1 | OR4C13 | p.266D>Y | 0.998 | mis | 0.003957 | rs140572207 |
| 198 | 11:104762062 | LNV | C/T | 1 | 1 | CASP12 | p.168V>M | 1.000 | mis | . | rs200223916 |
| 198 | 12:7310162 | LNV | C/T | 1 | 2 | CLSTN3 | p.869R>C | 1.000 | mis | . | rs750797901 |
| 198 | 14:92439151 | LNV | G/T | 1 | 1 | TRIP11 | p.1877P>T | 1.000 | mis | 0.004187 | rs148261539 |
| 198 | 15:66624309 | LNV | G/A | 1 | 1 | DIS3L | p.878G>R | 1.000 | mis | . | rs536121897 |
| 198 | 16:24816350 | LNV | A/G | 1 | 1 | TNRC6A | p.1334M>V | 0.999 | mis | 0.001047 | rs141294822 |
| 198 | 16:30594147 | LNV | C/T | 1 | 1 | ZNF785 | p.318G>S | 0.999 | mis | . | . |
| 198 | 16:50746086 | LNV | C/T | 1 | 2 | NOD2 | p.755A>V | 0.999 | mis | 0.004651 | rs61747625 |
| 198 | 17:10348369 | LNV | A/C | 1 | 1 | MYH4 | p.1797L>R | 1.000 | mis | . | . |
| 198 | 17:18051447 | LNV | C/T | 1 | 2 | MYO15A | p.2205T>I | 0.997 | mis | 0.003953 | rs121908970 |
| 198 | 18:28908281 | LNV | G/A | 1 | 1 | DSG1 | p.116D>N | 1.000 | mis | 0.000116 | rs150168711 |
| 198 | 18:30950117 | LNV | C/T | 1 | 1 | C18orf34 | p.82R>Q | 1.000 | mis | 0 | rs61745348 |
| 198 | 18:32443935 | LNV | G/A | 1 | 1 | DTNA | p.464R>H | 1.000 | mis | 0.000233 | rs142108185 |
| 198 | 19:55494283 | LNV | C/G | 1 | 1 | NLRP2 | p.406T>R | 0.999 | mis | 0.009302 | rs139903547 |
| 198 | 20:40050639 | LNV | G/A | 1 | 1 | CHD6 | p.1546R>W | 0.999 | mis | . | rs563059701 |
| 198 | 22:50943907 | LNV | G/A | 1 | 1 | LMF2 | p.326L>F | 0.999 | mis | 0.00314 | rs145130809 |
| 198 | X:18942637 | LNV | C/T | 1 | 2 | PHKA2 | p.526D>N | 0.997 | mis | . | rs778051353 |

**Supplementary Table S2.** Rare CNVs identified and validated in 15 Australian BD families.

| **PED** | | **Chr:Start-End (hg19)** | **LOD** | **Detection (V)** | **N Aff** | **N Unaff** | **Type (N)** | **Size kb (M)** | **Genes** |
| --- | --- | --- | --- | --- | --- | --- | --- | --- | --- |
| 17 | 3:173240621-173300050 | | 0.027 (NPL) | CS (qPCR) | 2/5 | 0/2 | Gain (3) | 59 (49) | *NLGN1* (intr) |
| 198 | 4:129770774-129936204 | | 0.045 (NPL) | CS (qPCR, PennCNV, ESC) | 3/4 | 2/2 | Gain (3) | 165 (176) | *PHF17, SCLT1* |
| 5 | 5:32109541-32170613 | | 1.3769 (PD) | CS (qPCR, PennCNV) | **5/5** | **0/2** | **Gain (4)** | **61 (60)** | ***PDZD2, GOLPH3*** |
| 1 | 5:140221139-140241040 | | -0.001 (NPE) | MendErr (qPCR, ESC) | 5/8 | 0/4 | Loss (1) | 20 (4) | *PCDHA8-9-10*  (PCDHA cluster) |
| 84 | 5:140221098-140249884 | | -0.10 (NPL) | MendErr (qPCR, ESC) | 2/3 | 1/1 | Loss (1) | 29 (4) | *PCDHA8-9-10*  (PCDHA cluster) |
| 129 | 5:140214381-140229086 | | -0.02 (NPE) | MendErr (qPCR, ESC) | 3/4 | 0/1 | Loss (1) | 14 (4) | *PCDHA7-8-9*  (PCDHA cluster) |
| 84 | 7:146203548-146334635 | | -0.10 (NPL) | CS (qPCR) | 1/3 | 0/1 | Loss (1) | 131 (340) | *CNTNAP2* (intr) |
| 18 | 8:73598461-73640595 | | -0.06 (NPL) | CS (qPCR) | 2/4 | 0/2 | Gain (3) | 42 (80) | *KCNB2* (intr) |
| 198 | 9:78342248-78390398 | | 0.852 (PD) | CS (qPCR, PennCNV) | 3/4 | 0/2 | Gain (3) | 48 (31) | close to *MIR548H3* |
| 2 | 11:99062606-99101692 | | -0.006 (NPE) | CS (qPCR, PennCNV) | 2/5 | 1/2 | Loss (1) | 39 (50) | *CNTN5* (intr) |
| 121 | 11:118081344-118195313 | | 1.194 (NPL) | CS (qPCR) | 4/4 | 0/2 | Gain (3) | 113 (132) | *AMICA1, MPZL3, MPZL2, CD3E* |
| 2 | 12:112181076-112304742 | | -0.074 (NPL) | CS (qPCR) | 2/5 | 0/2 | Gain (3) | 123 (116) | *ACAD10, ALDH2, MAPKAPK5-AS1, MAPKAPK5* |
| 18 | 16:75537695-75575410 | | 0.507 (PP) | CS (qPCR, ESC) | 3/4 | 1/2 | Gain (4) | 37 (40) | *CHST5, TMEM231* |
| 1 | 19:481923-516624 | | 0.1 (NPL) | CS (qPCR) | 7/8 | 1/4 | Gain (3) | 34 (40) | *MADCAM1, TPGS1* |
| 84 | 20:5694975-5730835 | | 0.01 (NPL) | CS (qPCR) | 2/3 | 0/1 | Loss (1) | 39 (25) | *close to C20orf196* |
| 2 | 22:18645073-19004731 | | 0.12 (NPL) | CS (qPCR) | 5/6 | 0/2 | Gain (3) | 359 (65) | *USP18, GGT3P, DGCR6, PRODH, DGCR5* |
| 1 | 22:18916842-19004772 | | 0.10 (NPL) | CS (qPCR) | 3/8 | 2/4 | Loss (1) | 88 (50) | *PRODH, DGCR5* |

N Aff, number of affected out of the total; N Unaff, number of unaffected out of the total; V, validation; N, number of copy; M, total markers/probes altered; NPL, non-parametric linear; NPE, non-parametric exponential; PD, parametric dominant; PP, parametric penetrance 0.6; CS, CytoScanHD array; qPCR, quantitative PCR; MendErr, Mendelian errors; ESC, CNV inferring using ESCAVATOR; PennCNV, CNV call from 660 SNPchip; intr, intronic CNV.

**Supplementary Table S3.** Validated *de novo* coding variants (n=31), found in 32 individuals from 9 BD families.

| **Chr:position** | **PED-IID** | **Ref/Alt** | **Affection Status** | **Gene** | **dbSNP ID** | **Change** |
| --- | --- | --- | --- | --- | --- | --- |
| 16:72094660-2 | 1-64 | GGA/- | BPI | *HP* ^NB^ | rs762487399 | inframe_indel |
| 19:57953362 | 1-64 | C/T | BPI | *ZNF749* | rs199969699 | p.42A>V |
| 1:150240440 | 1-65 | G/C | Unaffected | *APH1A* | - | p.67L>L |
| 10:124187853 | 2-221 | A/G | BPII | *PLEKHA1* | - | p.321E>G |
| 2:219294199 | 2-221 | C/T | BPII | *VIL1* | - | p.253L>L |
| 3:98536707 | 5-521 | T/A | BPI | *DCBLD2* | - | p.373E>V^c^ |
| 1:114253066 | 5-522 | C/G | BPI | *PHTF1* | - | p.360R>P^c^ |
| 5:79809539 | 5-522 | C/T | BPI | *FAM151B*^NB^ | - | p.90L>L |
| 16:30975973 | 5-523 | A/G | Unaffected | *SETD1A* | - | p.304S>G |
| 19:45851292 | 5-523 | C/T | Unaffected | *KLC3* | rs368152525 | p.218A>V |
| 19:13885321 | 5-524 | G/A | SZMA | *C19orf53* | - | p.10A>A |
| 8:144877669 | 11-1115 | G/A | Unaffected | *SCRIB* | rs782460123 | p.1212A>V |
| 1:158592802 | 11-1115 | A/G | Unaffected | *SPTA1* | - | p.2031L>L |
| 2:25461999 | 11-1115 | C/T | Unaffected | *DNMT3A* | rs764146514 | p.803R>K^S^ |
| 3:53852070 | 11-1115 | C/T | Unaffected | *CHDH* | - | p.507D>N^c^ |
| 8:48743230 | 11-1115 | C/G | Unaffected | *PRKDC* | - | p.2778E>Q |
| 1:231337214 | 11-1118 | G/A | Unaffected | *TRIM67* | - | p.495P>P |
| 1:20980731 | 18-1800 | G/A**^M^** | SZMA | *DDOST* | - | p.277A>V |
| 12:109876342 | 18-1804 | C/T | Unaffected | *MYO1H* ^NB^ | - | p.721A>A^S^ |
| 9:130206567 | 11-1804 | T/C | Unaffected | *ZNF79* | rs377682538 | p.196N>N |
| 8:22064885 | 18-1806 | C/T | BPI | *BMP1* | - | p.811R> X |
| 11:66633693 | 18-1806 | C/T | BPI | *PC* | - | p.384A>T |
| 16:2350069 | 19-1920 | G/A | SZMA | *ABCA3* | rs562947317 | p.516N>N |
| 3:47958042 | 19-1920 | T/C | SZMA | *MAP4* | - | p.425I>M |
| 12:64377852 | 113-11300 | C/T | BPI | *SRGAP1* | - | p.65R>W^c^ |
| 11:3838696 | 113-11302 | C/T | BPI | *PGAP2* | rs75812077 | p.150F>F |
| 15:83447636 | 113-11302 | G/A**^P^** | BPI | *FSD2* ^NB^ | - | p.331L>L^S^ |
| 17:74395538 | 113-11302 | T/C | BPI | *UBE2O* | - | p.540P>P |
| 10:5498065 | 113-11303 | C/G | SZMA | *NET1* | - | p.405L>V^c^ |
| 11:6789509 | 113-11306 | C/T | BPII | *OR2AG2* ^NB^ | rs569276635 | p.227R>H |
| 5:140870484 | 121-12113 | T/C | BPII | *PCDHGC5* | - | p.559D>D |

^M^ *De novo* variant of maternal origin; ^P^ *De novo* variant of paternal origin; ^c^, CAROL score>0.99. ^S^, Splice region variant (defined as within 1-3 bases of the exon or 3-8 bases of the intron); Ref = reference allele; Alt = alternative allele; PED-IID = unique subject identifier, incorporating pedigree ID followed by individual ID. ^NB^, These genes have negligible brain expression, with average RKPM<1 in Brainspan RNA-seq data.

**7. SUPPLEMENTARY FIGURES**


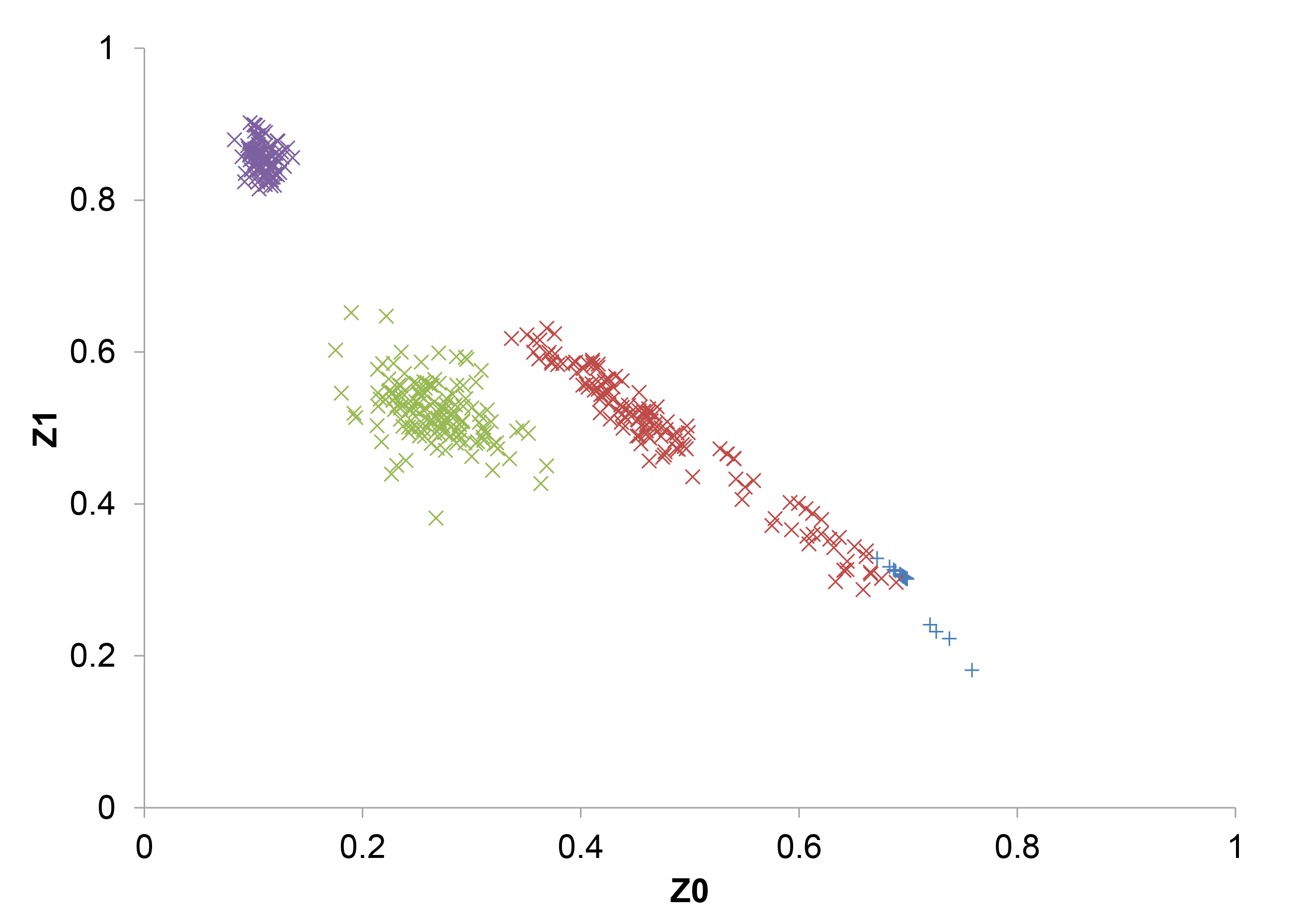


**Supplementary Figure S1. Genome-wide IBD confirmation of familial relationships from WES-derived genotype data.** Within families, parent-child pairs are indicated in purple (Pi hat range=0.434-0.496), sibling pairs in green (Pi hat=0.40-0.554), and more distant relatives in red crosses (Pi hat=0.162-0.355). Across families, 21 distant 3^rd^ degree relationships were identified (blue crosses; Pi hat=0.150-0.164), mainly involving subjects in pedigrees 18, 113 and 138.

**
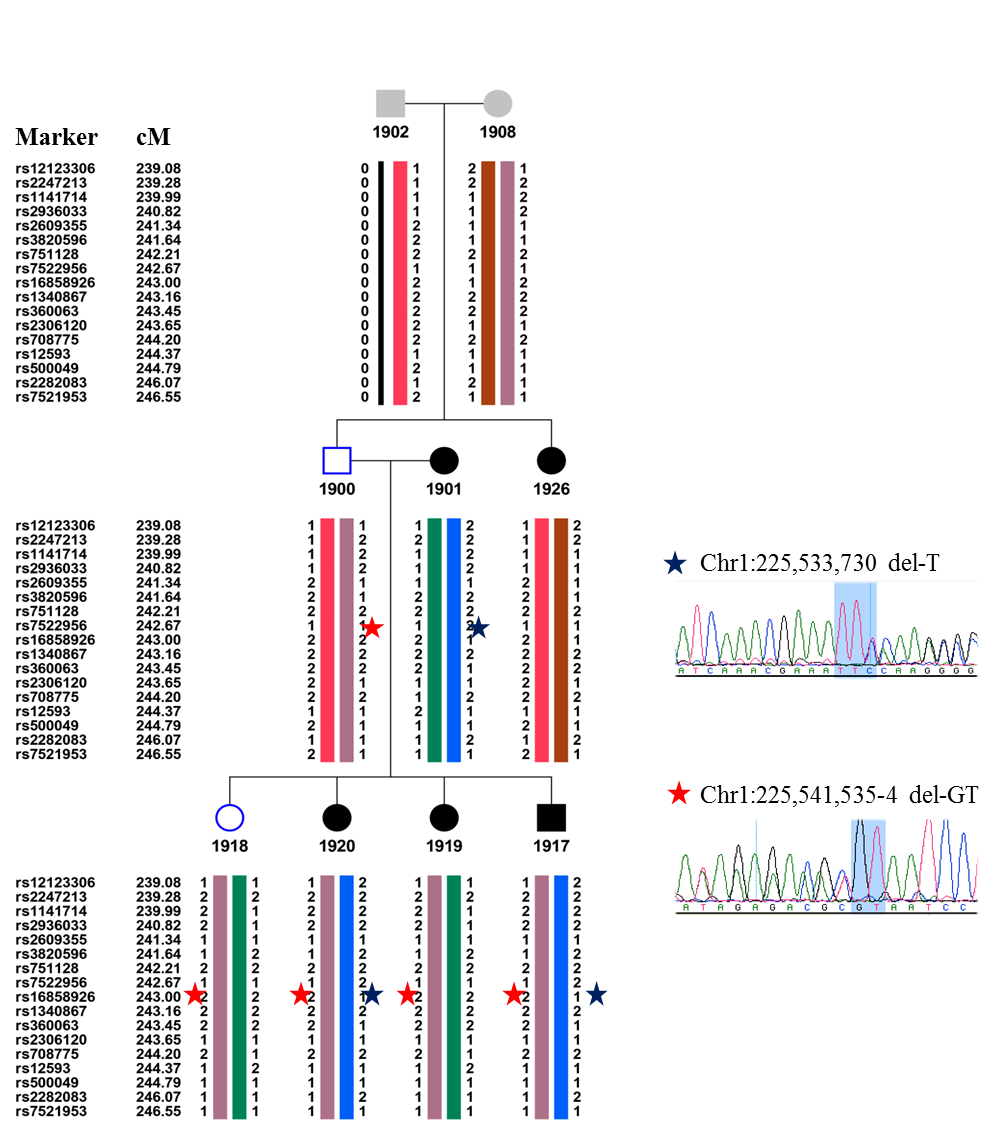
**

**Supplementary Figure S2. A complete knock-out of autosomal *DNAH14* gene by compound heterozygous mutations in pedigree 19.** The two indels causing protein translation frameshift of DNAH14 were transmitted from a different parent, resulting in a compound heterozygote in 2 affected individuals (1920 and 1917). Both indels were confirmed with Sanger sequencing, as shown.

**
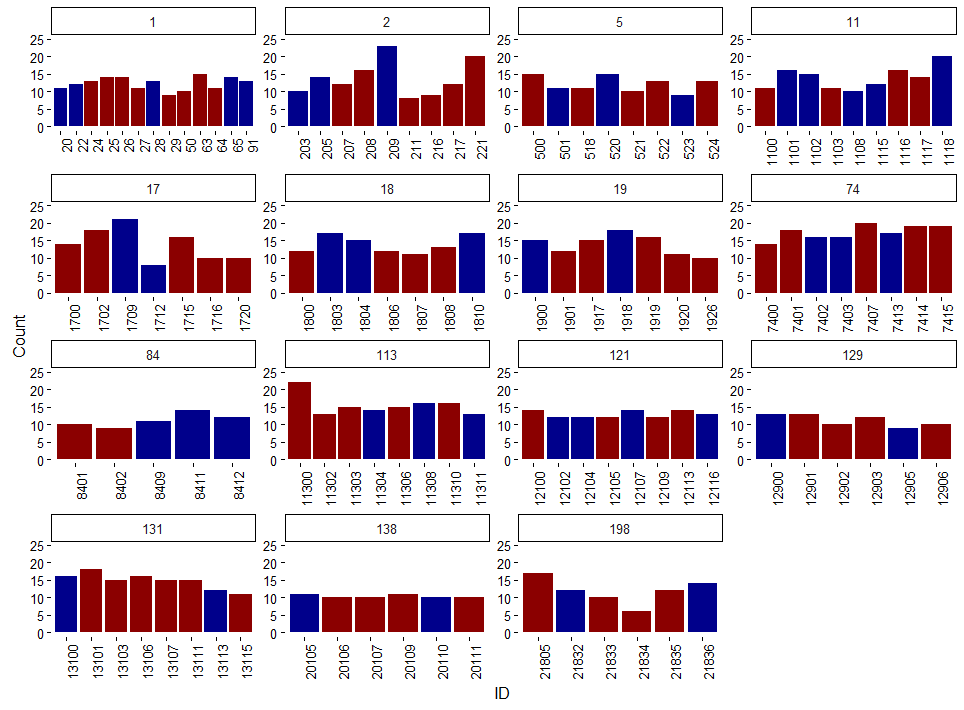
**

**Supplementary Figure S3. Total number of likely-gene-disruptive (LGD) variants per individual.** Subjects affected are represented in red and unaffected/unknown relatives are represented in blue. The family ID is indicated above, and subject ID is given along X-axis, with count of LGD variants per subject on Y-axis.

**
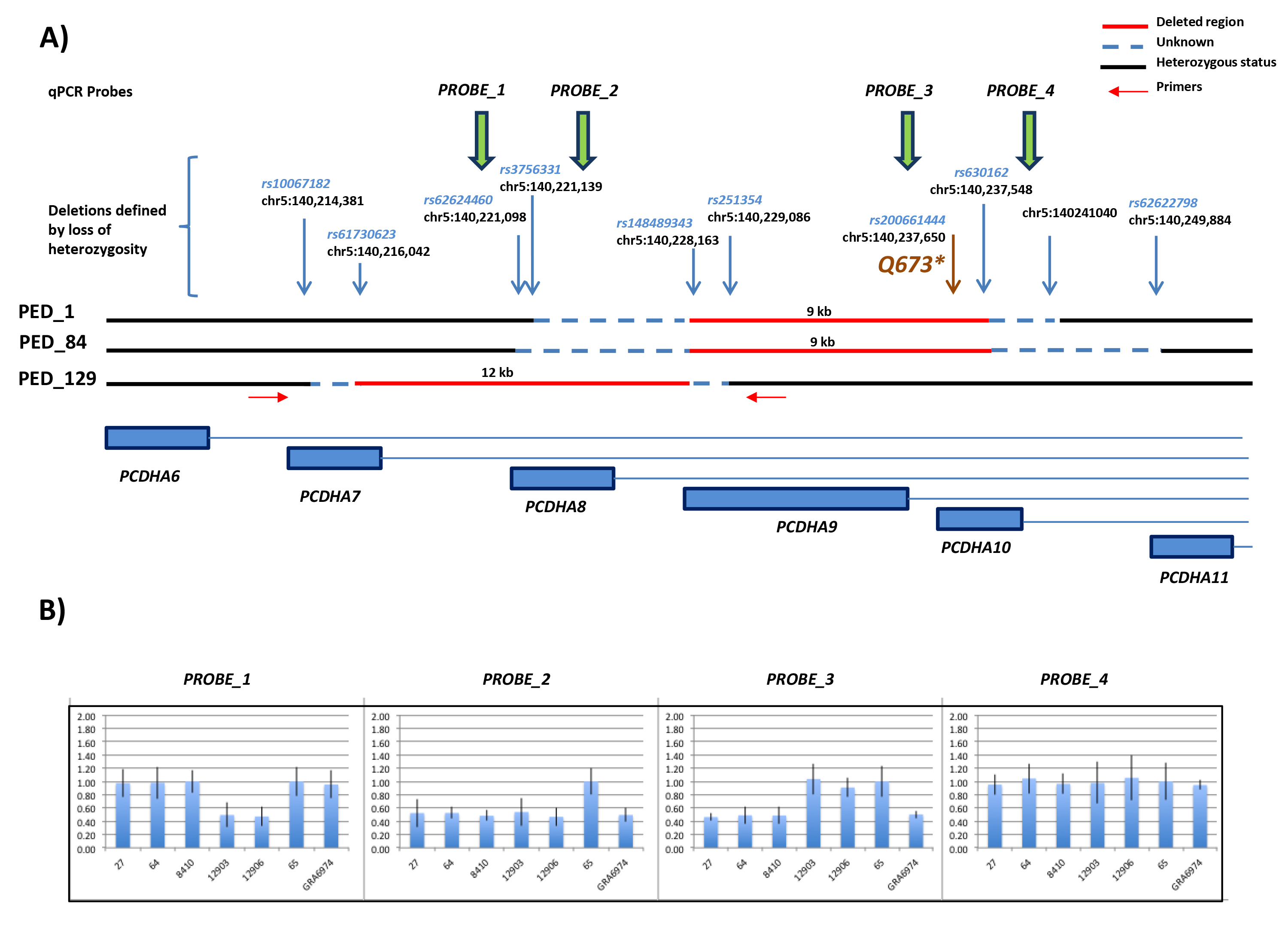
**

**Supplementary Figure S4**. **CNV deletions on chromosome 5q31.3 spanning the protocadherin alpha (PCDHA) gene cluster.** A) Deletions involving PCDHA7 to PCDHA10 were identified through Mendelian inconsistencies in WES data, where SNPs used to map the deletion boundaries across the 3 families, and their genomic coordinates, are shown. Along with a deletion segregating in family 84, a stop mutation was also found in PCDHA10 (rs200661444, Q673*). Dashed lines represent regions where deletion status, inferred from SNP information, is unknown. Four qPCR amplicon probes (green arrows) were designed to validate the CNV deletions by SYBR Green-based qPCR assay, and one breakpoint PCR (red arrows) designed to determine exact CNV. B) Example of CNV validation results, using the four qPCR amplicon probes in individuals from pedigree 1 (individuals 27, 64), pedigree 84 (individual 8410), pedigree 129 (individuals 12903 and 12906), plus a positive control for the deletion (GRA6974) and a wild-type DNA (individual 65 from pedigree 1). All available individuals from these three families were included in the validation.

**
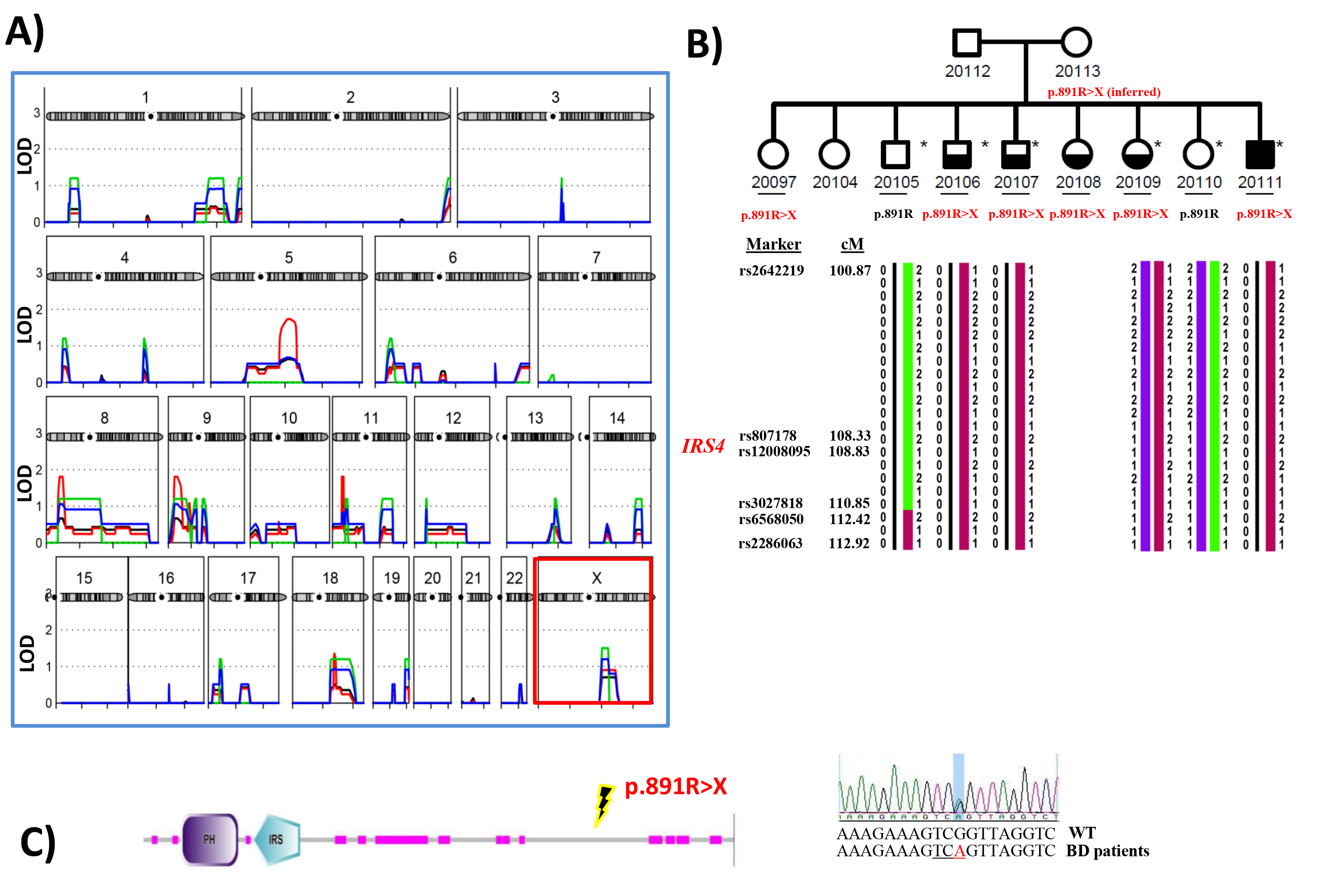
**

**Supplementary Figure S5. Nonsense variant in the X-linked gene *IRS4*, coinciding with a linkage peak in pedigree 138.** A) Linkage plots for pedigree 138 under parametric (dominant model: 100% penetrance in green, 60% penetrance in blue) and nonparametric models (linear in black, exponential in red). The red box around chromosome X highlights the maximum parametric LOD score (dominant model, LOD=1.5). B) Structure of pedigree 138. Females and males are represented by circles and squares respectively, with diagnosis indicated by sharing: BD-I (fully shaded) and schizoaffective disorder (half shaded). Subjects who underwent WES (asterisk), and those with DNA available (underlined) are indicated. The *IRS4* protein-truncating mutation (chrX:107976904G>A; p.891R>X) was transmitted from the mother (inferred) to 4/4 affected siblings and 0/2 unaffected siblings with WES. The mutant *IRS4* allele lies between markers rs807178 and rs12008095 on the maternal haplotype shown in burgundy. The p.891R>X variant was validated by Sanger sequencing, and genotyped in all relatives with DNA available, and was found in 5/5 affected siblings and 1/3 unaffected siblings. The unaffected mutation carrier (20097) was the youngest daughter (aged 22 at diagnostic interview). The current diagnostic status of subject 20097 remains unknown. C) Representation of IRS4 protein and functional domains, with the position of the stop mutation shown, relative to the Plekstrin (PH) and insulin receptor substrate (IRS) protein domains.

**
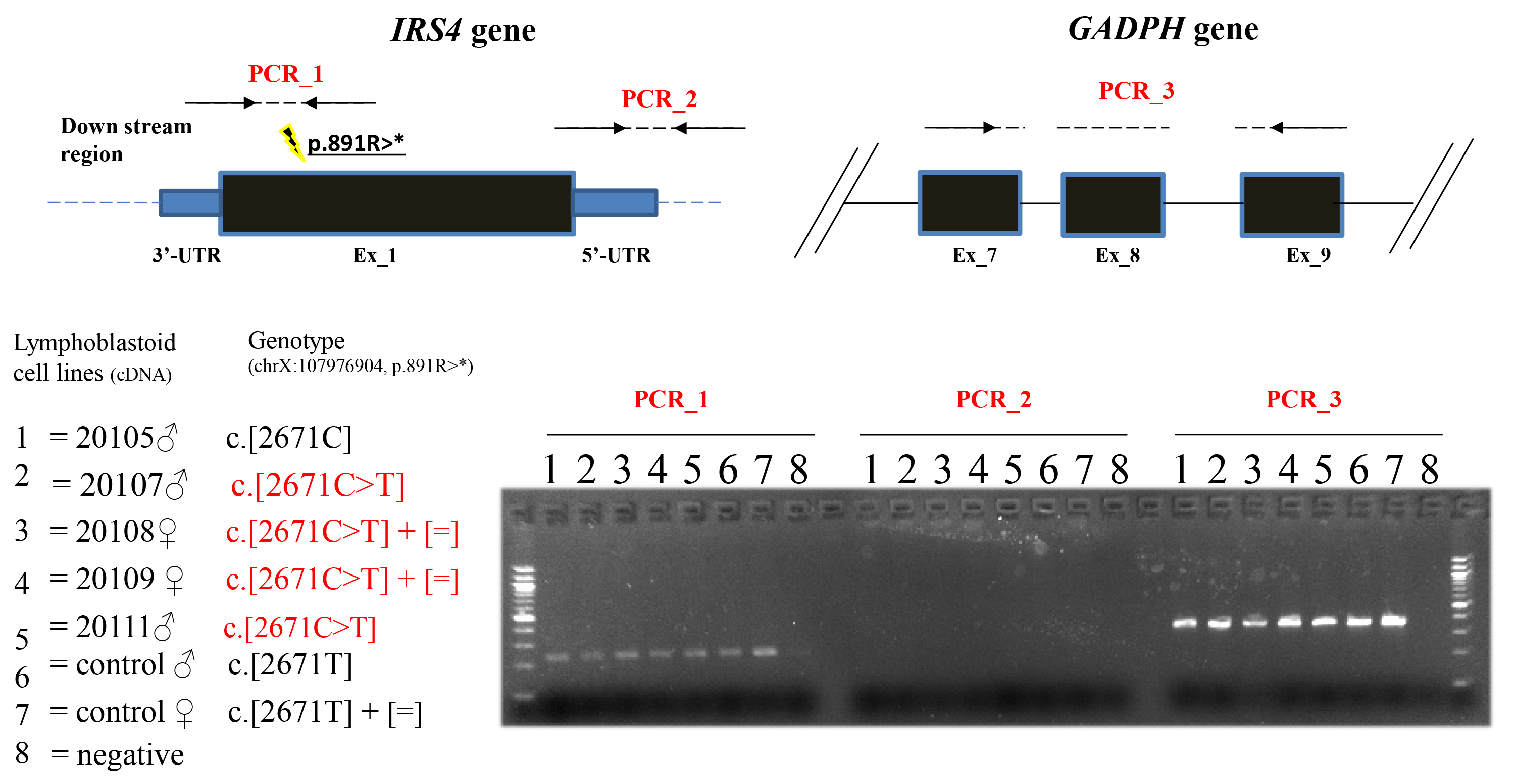
**

**Supplementary Figure S6**. **Lack of evidence of nonsense-mediated mRNA decay (NMD) for IRS4 c.[2671C>T] (p.891R>X) variant.** RNA was extracted from EBV immortilised lymphocyte cell lines from each member of family 138, and reverse transcribed to cDNA, which was PCR amplified using three amplicons: PCR_1 includes the site of nonsense mutation, showing transcript expression in mutation carriers as well as non-carriers; PCR_2 was a genomic DNA amplification control, showing no gDNA contamination of cDNA; PCR_3 was a positive control for *GADPH* housekeeping gene (cDNA amplification across exons 7-9). All family members with available lymphoblastoid cell lines were included in the study (lanes 1-5), plus two controls (one male, one female; lanes 6&7) and an RT- negative control (lane 8).





**Supplementary Figure S7. Nine nuclear BD families under examination for *de novo* variants.** A total of 32 individuals (22 affected and 10 unaffected; marked with an asterisk) for whom both parents WES were available were included in the study.

**
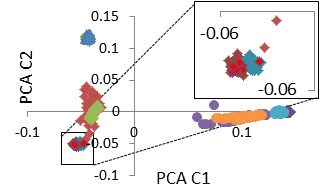
**

**Supplementary Figure S8. Estimation of family ancestry by Multidimensional Scaling Analysis.** European, Asian and African origin samples are shown in diamond, triangle and circle shapes respectively. One member from each of 15 WES families are shown in red diamonds, and overlap completely with CEU (brown), TSI (cyan). See inset for zoom in of European cluster. The Asian cluster (top left) contains subjects of CHB (sage), JPT (orange) and CHD (blue) origin. The African cluster (bottom right) contains subjects of LWK (light blue), MKK (light orange) and YRI (purple) origin.
